# Identification and characterization of BAHD hydroxycinnamoyltransferases in the fern *Neoblechnum brasiliense*

**DOI:** 10.1111/tpj.70837

**Published:** 2026-03-29

**Authors:** Maximilian Ufland, Maike Petersen

**Affiliations:** ^1^ Institut für Pharmazeutische Biologie und Biotechnologie Philipps‐Universität Marburg Robert‐Koch‐Str. 4 35037 Marburg Germany

**Keywords:** hydroxycinnamoyltransferases (HCT), BAHD, *Neoblechnum brasiliense* (Blechnaceae), phenolic metabolism, phenylpropanoid derivatives, chlorogenic acid, caffeoylshikimic acid, rosmarinic acid

## Abstract

*Neoblechnum brasiliense*, a leptosporangiate fern of the family Blechnaceae, is known to have a rich phenolic metabolism. Rosmarinic acid and blechnic acid B have been detected beside chlorogenic acid. As BAHD acyltransferases often are responsible for the formation of this kind of esters, for example, rosmarinic acid synthases or 4‐coumaroyl‐CoA:shikimic or quinic acid hydroxycinnamoyltransferases, nine coding sequences for BAHD acyltransferases were amplified from *N. brasiliense* and heterologously expressed. All enzymes proved to be active with *p*‐coumaroyl‐CoA as acyl donor as well as other hydroxycinnamoyl‐CoA derivatives. Four enzymes (NbHCT4, 6, 7, 9) accepted various alcohols and amines but only to a very low extent. High catalytic efficiency was observed for NbHCT1 and 2 that preferred shikimic acid as acceptor substrate, while NbHCT3 was most active with quinic acid and is part of a lineage‐specific duplication subclade of clade V with sequences of Cyatheales and Polypodiales. Tryptamine amides were formed by NbHCT5. NbHCT8 not only preferred 3‐hydroxyanthranilate but also transformed shikimic and quinic acids. NbHCTs 1, 3, and 8 were fully kinetically characterized. In a phylogenetic tree comprising members of all known BAHD clades, NbHCT1, 2, 3, and 8 were grouped into clade V harboring most known shikimate and quinate hydroxycinnamoyltransferases, while NbHCT6 and 7 were in or near clade VI. NbHCT4 was placed in clade IV, NbHCT5 most probably in clade IV and NbHCT9 in clade I. Since no hydroxycinnamoyltransferase forming rosmarinic acid could be identified, further hydroxycinnamoyltransferases must be expected in *N. brasiliense*.

## Significance Statement

Nine BAHD acyltransferases have been identified and characterized in the fern *Neoblechnum brasiliense* transferring hydroxycinnamoyl units onto various alcohol and amine acceptor substrates. Kinetic characterization showed that shikimic and quinic acids for ester formation as well as 3‐hydroxyanthranilic acid and tryptamine for amide formation are the main substrates.

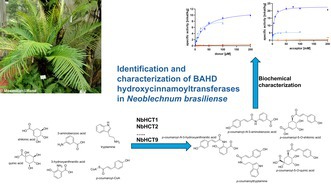

## INTRODUCTION

Hydroxycinnamic acid esters, such as chlorogenic acid and rosmarinic acid, occur widely throughout the plant kingdom presumably as UV screen or feeding and pathogen deterrent (Petersen, 2013). Caffeoylshikimic acid, on the other hand, is essential in the formation of caffeoyl, feruloyl, and sinapoyl moieties and monolignols as building blocks for the cuticle and for lignification (Boerjan et al., 2003; Renault et al., 2017). The formation of these esters has generally been associated with the activity of hydroxycinnamoyltransferases (HCTs) from the BAHD superfamily (Moghe et al., 2023). During the last three decades, ample knowledge has been accumulated regarding the properties of BAHD HCTs mainly from gymno‐ and angiosperms, while enzymes from non‐seed plants are less well investigated. BAHDs from the moss *Physcomitrium patens* (Kriegshauser et al., 2021), the hornwort *Anthoceros agrestis* (Ernst et al., 2022), or the liverwort *Marchantia polymopha* (Kruse et al., 2022) have been characterized before and this shows that hydroxycinnamoyl‐CoA:shikimic acid or quinic acid hydroxycinnamoyltransferases (HST, HQT) are conserved in bryophytes. HCTs in Monilophyta were biochemically investigated in a eusporangiate fern (*Equisetum arvense*, Hohlfeld et al., 1996), but HCTs from leptosporangiate ferns have not yet been characterized in more detail. Therefore, our interest was raised for the leptosporangiate fern species *Neoblechnum brasiliense* (Desv.) Gasper & V.A.O. Dittrich in the family Blechnaceae, which has been found to contain rosmarinic acid, isorinic acid, and blechnic acid B besides chlorogenic acid (Harborne, 1966; Ufland & Petersen, 2026). (–)‐*cis*‐Brainic and (–)‐*cis*‐ and (–)‐*trans*‐blechnic acids have been found in a related species, *Blechnum spicant* (syn. *Struthiopteris spicant*) (Wang et al., 2001). The family Blechnaceae is part of the suborder Aspleniieae (Eupolypods II) of the order Polypodiales. *N. brasiliense* is the only species in the genus *Neoblechnum* and native to the Neotropics (de Gasper et al., 2016). Generally, ferns show a broad range of specialized metabolites like flavonoids, terpenoids, steroids, and polyphenols (Cao et al., 2017; Waswa et al., 2022). This led to the use of ferns in Traditional Medicines for a broad range of diseases: for example, skin disorders or pulmonary diseases (Waswa et al., 2022). An anti‐inflammatory effect of rosmarinic acid isolated from *N. brasiliense* was observed in adult zebrafish brains by a reduced LPS induction of TNF‐α and IL‐1β (Fasolo et al., 2021). Moreover, the neuroprotective potential of *N. brasiliense* extracts by selective MAO‐A inhibition, putatively by rosmarinic or chlorogenic acid, was discussed (Andrade et al., 2016).

Besides *N. brasiliense*, also some other ferns are known to contain RA: *Doodia maxima*, *Lomariocycas tabularis*, *Oceaniopteris cilitata* (all Blechnaceae, Polypodiales) (Ufland & Petersen, 2026), *Azolla filiculoides* (Salviniaceae, Salviniales) (Carballo‐Sanchez et al., 2022), and *Diplazium esculentum* (Athyriaceae, Polypodiales) (Kongsung et al., 2024). Generally, RA is mainly found in Boraginaceae and the subfamily Nepetoideae of the Lamiaceae but was also described in hornworts (Takeda et al., 1990), Chloranthaceae (Bömeke & Petersen, 2025; Petersen et al., 2009; Zhu et al., 2008), and various monocots and other dicots (Petersen et al., 2009).

The biosynthetic pathway for RA was described in 1993 for *Coleus blumei* (syn. *Coleus scutellarioides*) (Petersen et al., 1993). The enzyme responsible for the transacylation of *p*‐coumaroyl‐/caffeoyl‐CoA with (*R*)‐4‐hydroxyphenyllactic acid/(*R*)‐3,4‐dihydroxyphenyllactic acid is “rosmarinic acid synthase” (RAS; hydroxycinnamoyl‐CoA:hydroxyphenyllactic acid hydroxycinnamoyltransferase). The *C. blumei* RAS activity was characterized in plant extracts (Petersen & Alfermann, 1988) and the encoding nucleotide sequence later heterologously expressed and kinetically analyzed (Berger et al., 2006). The enzyme is a member of the BAHD acyltransferase superfamily and has additionally been investigated in various other plants, for example, *Melissa officinalis* (Weitzel & Petersen, 2011), *Lavandula angustifolia* (Landmann et al., 2011), *Sarcandra glabra* (Bömeke & Petersen, 2025), or *Lithospermum erythrorhizon* (Ogata et al., 2004).

BAHD acyltransferases catalyze the formation of an ester or amide using CoA‐activated acids (acyl donors) and an alcohol or amine (acyl acceptors). The enzyme superfamily is widespread in the plant kingdom and named after the first four identified enzymes of this superfamily (St. Pierre & de Luca, 2000). It is characterized by two conserved sequence motifs. The motif HxxxDG is located in the *N*‐terminal half and harbors the catalytically active histidine residue. The motif DFGWG is localized more to the *C* terminus and plays a role in substrate binding and positioning (D'Auria, 2006; St. Pierre & de Luca, 2000). A wide range of compounds is formed by BAHD acyltransferases from relatively simple molecules such as benzyl benzoate or hexyl acetate to complex natural products such as paclitaxel or vindoline (D'Auria, 2006; Moghe et al., 2023). BAHD acyltransferases in pteridophytes are of particular interest as the number of BAHDs during plant evolution increased from one to five BAHD copies in algae to 50–200 BAHD copies in angiosperms; ferns (*Salvinia cucullata*, *A. filiculoides*) contain less than 20 BAHD copies (Moghe et al., 2023). From these, clade V BAHD acyltransferases are responsible for the formation of RA, chlorogenic acid (caffeoyl‐5‐*O*‐quinic acid) or caffeoyl‐5‐*O*‐shikimic acid (Moghe et al., 2023). The latter is especially important since it is an intermediate in the formation of caffeyl‐ (C), guajacyl‐ (G), and syringyl‐ (S) monolignols (Ma, 2024). While bryophytes are considered not to contain real lignin, Lycopodiophyta and Pteridophyta are the first land plants to have lignified xylem. Due to the ambiguous nomenclature of *p*‐coumaroyl and caffeoyl esters with shikimic and quinic acids, their chemical structures and nomenclature according to Abrankó and Clifford (2017) are provided in Figure S1.

In order to access the molecular and biochemical background for the formation of hydroxycinnamic acid esters in *N. brasiliense*, we first identified putative HCT sequences in the transcriptome of a close relative to the RA‐producing fern *N. brasiliense* (formerly *B. brasiliense*) with an accessible transcriptome, namely *S. spicant* (formerly *B. spicant*), and amplified the corresponding DNA sequences from *N. brasiliense* in order to express them heterologously and perform a full biochemical characterization. The results for five active HCTs from *N. brasiliense* will be presented here.

## RESULTS

### Identification of BAHD acyltransferase sequences

Due to the presence of various caffeoyl esters in *N. brasiliense* (Ufland & Petersen, 2026), among them RA and its derivatives, we aimed at identifying the responsible genes and enzymes and concentrated on those of the BAHD superfamily. We performed BLASTP searches in the transcriptome of *S. spicant* (syn. *B. spicant*), a related species without RA accumulation (1kP database, taxid: 114457, sample code: VITX; Carpenter et al., 2019; Leebens‐Mack et al., 2019), using the RAS amino acid sequence from *C. blumei* (syn. *C. scutellarioides*) (UniProt A0PDV5; Berger et al., 2006) as a query with the assumption that the acyltransferase responsible for the formation of precursors of, for example, rosmarinic acid in ferns is just like in Boraginaceae, Lamiaceae, and Chloranthaceae a BAHD acyltransferase. By this, we found several sequences coding for BAHD acyltransferases (Table S1). Scaffold 2003763 was of special interest, as it is the only sequence having the “lysine‐handle” as described by Levsh et al. (2019), which is putatively involved in binding and placement of 4‐hydroxyphenyllactic acid, and was therefore included in our investigation. The selected scaffolds served as primer templates for amplifying promising homologs in *N. brasiliense*.

*NbHCT* sequences were amplified based on the sequences extracted from the *S. spicant* 1 kP database either directly (*NbHCT1*) or with help of 3′‐ and 5′‐RACE PCR (*NbHCT2‐9*). *NbHCT6* and *NbHCT7* were found by applying primers directed against a degenerated DFGWG motif. No homolog was found for them in *S. spicant*. *NbHCT1* was used as a codon‐optimized sequence as a successful protein expression could only be achieved after codon optimization for the expression host *Escherichia coli*. Full‐length sequences were ligated into the expression vector pET‐15b which was introduced into *E. coli* SoluBL21 for heterologous expression. Protein extracts from the transformed bacteria were subjected to SDS‐PAGE and western blot analyses for verification of protein expression (Figure S2). Table 1 shows general data for all NbHCTs further analyzed in this report.

**Table 1:** BAHD acyltransferase sequences identified in *Neoblechnum brasiliense* with their GenBank accessions, the homologous sequences in *Struthiopteris spicant* (1kP database, VITX—if applicable), the length of the coding sequence and the native protein, the molecular mass of the native protein, the calculated isoelectric point and the proven catalytic activity shown in this study

Sequence	GenBank accession	Homologous sequence in *S. spicant* VITX_scaffold_…	Length molecular mass isoelectric point	Catalytic activity (this study)
*NbHCT1*	PV344582	2007085	1575 bp/524 aa 58 755.40 Da pH 7.54	Hydroxycinnamoyl‐CoA:shikimic acid HCT
*NbHCT2*	PV344583	2011375	1356 bp/451 aa 49 896.20 Da pH 6.60	Hydroxycinnamoyl‐CoA:shikimic/quinic acid HCT
*NbHCT3*	PV344584	2011306	1311 bp/436 aa 49 014.85 Da pH 7.89	Hydroxycinnamoyl‐CoA:quinic acid HCT
*NbHCT4*	PV344585	2009524	1284 bp/427 aa 46 926.43 Da pH 7.2	No main substrate identified
*NbHCT5*	PV344586	2014301	1305 bp/434 aa 47 979.90 Da pH 5.66	Hydroxycinnamoyl‐CoA:tryptamine HCT
*NbHCT6*	PV344587	No scaffold	1407 bp/468 aa 51 149.29 Da pH 5.72	No main substrate identified
*NbHCT7*	PV344588	No scaffold	1404 bp/467 aa 50 617.66 Da pH 5.88	No main substrate identified
*NbHCT8*	PV344589	2099633	1881 bp/626 aa 69 573.64 Da pH 5.59	Hydroxycinnamoyl‐CoA:3‐hydroxyanthranilic acid HCT
*NbHCT9*	PV344590	2003763	1293 bp/430 aa 48 066.99 Da pH 5.90	No main substrate identified

### Substrate search for BAHD acyltransferases from *N. brasiliense*

**Figure 1:**
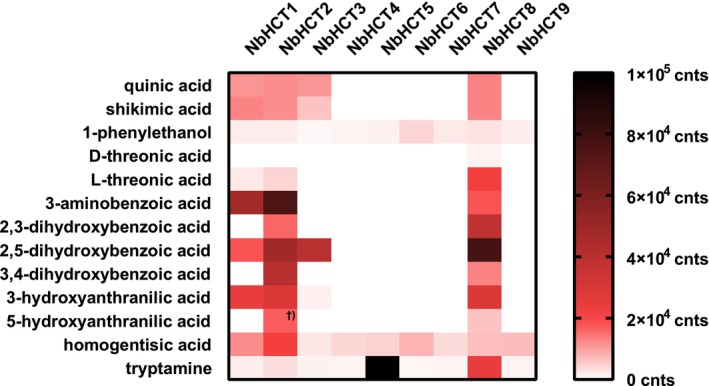
Double gradient heat map of extracted ion chromatograms (EIC) of substrate search assays with varying acyl acceptors (left side) and *p*‐coumaroyl‐CoA as acyl donor. Gradient reaching from 0 counts (cnts) to 2 × 10^4^ cnts (white to red) and 2 × 10^4^ cnts to 1 × 10^5^ cnts (red to black). Note that a direct comparison between different acyl acceptors is not possible due to different ionization potential or detection methods. ^†^Product formation by NbHCT2 resulted in a different retention time than the reference substance (amide) suggesting the formation of an ester.

A substrate search using various hydroxycinnamoyl‐CoA thioesters as acyl donor and 70 different putative acceptor substrates was performed to assess the activities of the expressed NbHCT proteins. NbHCT1, NbHCT2, NbHCT3, and NbHCT8 transform a number of different acyl acceptors and donors as described in Table S2. The biochemical characterization of NbHCT1 showed that 3‐hydroxyanthranilate and shikimate were about equally accepted as acyl acceptors and had a clear preference for *p*‐coumaroyl‐CoA as acyl donor (Table S3; Figure 1). NbHCT2 accepted shikimate, 3‐hydroxyanthranilate, and quinate. NbHCT3 preferentially forms 5‐*O*‐quinic acid esters with either *p*‐coumaric or caffeic acid, with a slight preference for the latter. NbHCT8 catalyzed the formation of many esters and amides, being the most promiscuous enzyme. Biochemical characterization showed that 3‐hydroxyanthranilic acid is the preferred acyl acceptor and *p*‐coumaroyl‐CoA and caffeoyl‐CoA are equally well accepted, with a slight preference for caffeoyl‐CoA at lower concentrations. NbHCT5 accepted tryptamine. NbHCT4, NbHCT6, NbHCT7, and NbHCT9 showed low but detectable product formation of *p*‐coumaroylhomogentisic acid, *p*‐coumaroyl‐*N*‐tryptamine, and *p*‐coumaroyl‐1‐phenylethanol. No substrate yielded product formation substantially above background. Detailed information is shown in Tables S4–S12.

#### Biochemical characterization of NbHCT1

NbHCT1 accepts a wide range of acyl acceptors and acyl donors for the formation of various esters and amides (see Tables S3 and S4; Figure 1; Figure S8). The best acceptors are shikimic, quinic, and 3‐hydroxyanthranilic acids. With shikimic and quinic acid, the formation of 5‐*O*‐esters is catalyzed, while an amide is formed with 3‐hydroxyanthranilic acid. To a very low extent, also the 4‐*O*‐ester of shikimic acid is observed as a product. The formation of 4/5‐*O*‐shikimic/quinic acid esters in this study was verified with the help of commercial 3/4/5‐*O*‐ester standards and their distinct fragmentation patterns as described by Clifford et al. (2003) and derivation from caffeoyl‐3/4/5‐*O*‐shikimic acid standards for formed *p*‐coumaroyl‐4‐*O*‐shikimic acids (Figure S9). The amide (*p*‐coumaroyl‐*N*‐3‐hydroxyanthranilic acid) was verified by comparing its fragmentation pattern and retention time with a chemically synthesized standard, which had been verified by NMR (Ernst et al., 2022). Further used acyl acceptors were l‐threonic, 3‐aminobenzoic, and 2,5‐dihydroxybenzoic acids. Assays with 1‐phenylethanol, homogentisic acid, and tryptamine result in weak product formation. Cinnamoyl‐CoA, *p*‐coumaroyl‐CoA, caffeoyl‐CoA, feruloyl‐CoA, sinapoyl‐CoA, and benzoyl‐CoA are all accepted with shikimic acid as acyl acceptors. The highest activity was measured at pH 6.97 and 33°C for the formation of caffeoyl‐5‐*O*‐shikimic acid (Figure S3; Table 2).

**Table 2:** Biochemical data for NbHCT1 (hydroxycinnamoyl‐CoA:shikimic acid hydroxycinnamoyltransferase); *n* = 3 × 3 ± SEM, *k*_cat_ is calculated with the molar mass of the protein (including 6x‐His‐Tag) of 61.3 kDa

Kinetic data for	With constant substrate	*K*_m_ (μM)	*V*_max_ (mkat kg^−1^)	*k*_cat_ (sec^−1^)	*k*_cat_/*K*_m_ (L mol^−1^ sec^−1^)
Caffeoyl‐CoA	Shikimic acid	27.64 ± 0.73	0.138 ± 0.002	0.0085 ± 0.0001	307.55 ± 10.65
Shikimic acid	Caffeoyl‐CoA	1134.67 ± 153.56	0.185 ± 0.015	0.0114 ± 0.0009	10.28 ± 1.35
*p*‐Coumaroyl‐CoA	Shikimic acid	60.13 ± 2.92	3.209 ± 0.284	0.1967 ± 0.0174	3280.89 ± 305.92
Shikimic acid	*p*‐Coumaroyl‐CoA	1556.00 ± 31.34	2.899 ± 0.102	0.1777 ± 0.0063	114.43 ± 6.20
*p*‐Coumaroyl‐CoA	Quinic acid	29.36 ± 1.73	0.392 ± 0.049	0.0240 ± 0.0030	823.78 ± 111.93
Quinic acid	*p*‐Coumaroyl‐CoA	30 146.46 ± 2954.84	0.256 ± 0.005	0.0157 ± 0.0003	0.53 ± 0.04
*p*‐Coumaroyl‐CoA	3‐Hydroxyanthranilic acid	4.92 ± 0.42	0.291 ± 0.018	0.0178 ± 0.0011	3646.68 ± 252.46
3‐Hydroxyanthranilic acid	*p*‐Coumaroyl‐CoA	226.29 ± 138.37	0.229 ± 0.025	0.0140 ± 0.0015	112.94 ± 44.60
pH optimum	6.97		Temperature optimum	33.1°C	

**Figure 2:**
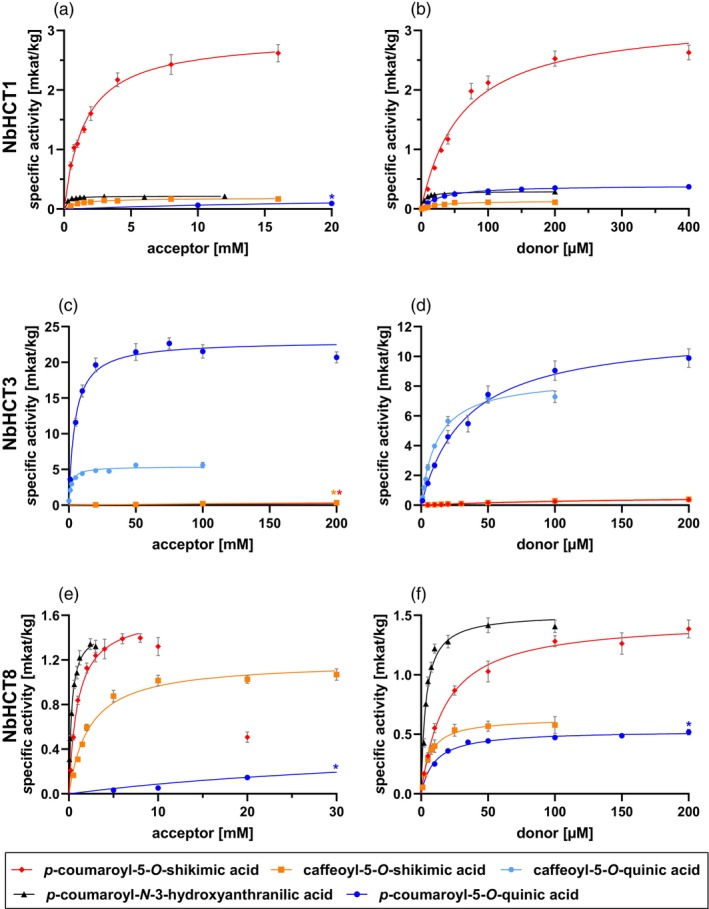
Substrate saturation curves of three *N. brasiliense* HCTs. Substrate saturation curves for NbHCT1 (a, b), NbHCT3 (c, d), and NbHCT8 (e, f) with variable concentrations of acceptors (left side) and donors (right side) (*n* = 9 ± SEM, except for graphs with shikimic acid for NbHCT3: *n* = 3 ± SEM, evaluated with Cornish–Bowden plots (Cornish‐Bowden & Eisenthal, 1978). Graphs marked with an asterisk (*) are not fully shown for better illustration, please refer to Figures S4, S6, S7 for complete display of all graphs.

Substrate saturation curves for acyl acceptors and donors were recorded for caffeoyl‐5‐*O*‐shikimic acid, *p*‐coumaroyl‐5‐*O*‐shikimic acid, *p*‐coumaroyl‐5‐*O*‐quinic acid, and *p*‐coumaroyl‐*N*‐3‐hydroxyanthranilic acid (Figure 2a,b; Figure S4; Table 2). 3‐Hydroxyanthranilic acid (*K*_m_ 0.2 mM) has the highest affinity in comparison to shikimic acid (*K*_m_ 1.2 mM) and quinic acid (*K*_m_ 30.1 mM) when forming the *p*‐coumaroyl derivatives. However, the turnover number for shikimic acid (0.18 sec^−1^) is 10‐fold higher than for 3‐hydroxyanthranilic acid (0.014 sec^−1^) and quinic acid (0.016 sec^−1^), resulting in approximately the same catalytic efficiency for 3‐hydroxyanthranilic acid (112.94 L mol^−1^ sec^−1^) and shikimic acid (114.43 L mol^−1^ sec^−1^), while the catalytic efficiency with quinate (0.53 L mol^−1^ sec^−1^) is 200 times lower. The catalytic efficiency for shikimate with caffeoyl‐CoA (10.28 L mol^−1^ sec^−1^) is 10 times lower than the one with *p*‐coumaroyl‐CoA (114.43 L mol^−1^ sec^−1^), resulting in a preference for *p*‐coumaroyl‐CoA over caffeoyl‐CoA. This is further confirmed when looking at the kinetic data for the CoA‐derivatives with constant concentrations of shikimic acid: the *K*_m_‐value for caffeoyl‐CoA (*K*_m_ 27.64 μM) is half the value for *p*‐coumaroyl‐CoA (*K*_m_ 60.13 μM) and the turnover number for *p*‐coumaroyl‐CoA is 20 times higher (0.197 versus 0.009 sec^−1^), resulting in a 10‐fold higher catalytic efficiency for *p*‐coumaroyl‐CoA over caffeoyl‐CoA (3280.89 versus 307.55 L mol^−1^ sec^−1^). The catalytic efficiency for *p*‐coumaroyl‐CoA with 3‐hydroxyanthranilate is comparable (3646.68 L mol^−1^ sec^−1^), while the catalytic efficiency for *p*‐coumaroyl‐CoA with quinate was significantly lower (823.78 L mol^−1^ sec^−1^).

#### Biochemical characterization of NbHCT2

**Figure 3:**
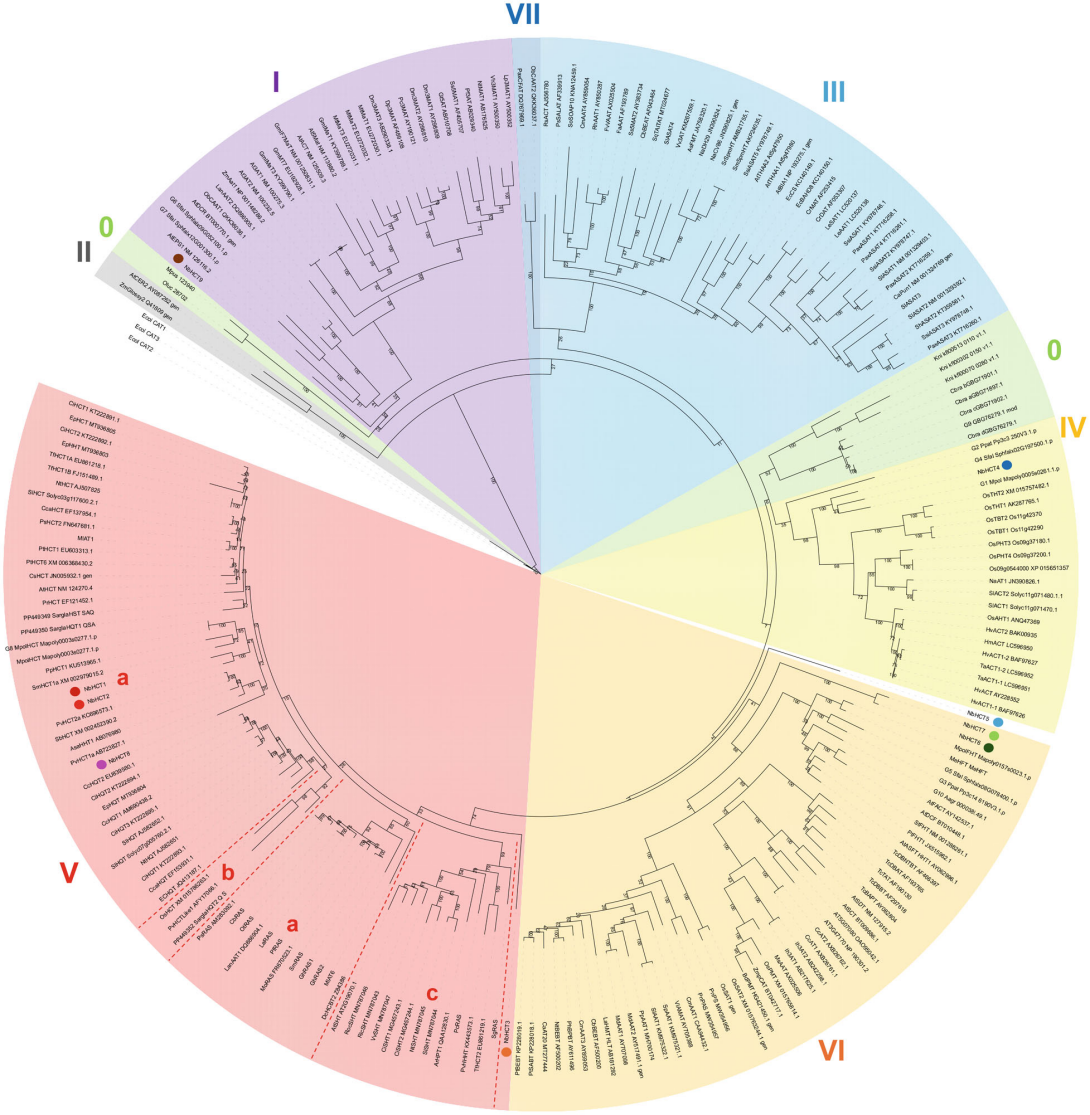
Phylogenetic tree of BAHD acyltransferases. 1000 bootstrap replicates. *Neoblechnum brasiliense* BAHDs are placed in clades I, IV, V, and VI. The tree was rooted using three chloramphenicol acyltransferases from *Escherichia coli*, which is considered to be one of the origins of BAHD acyltransferases. Clade numbering and subclades of clade V following Kruse et al. (2022). Dots mark NbHCTs: NbHCT1 dark red, NbHCT2 light red, NbHCT3 orange, NbHCT4 dark blue, NbHCT5 light blue, NbHCT6 dark green, NbHCT7 light green, NbHCT8 pink, NbHCT9 brown.

NbHCT2 accepts a very broad range of acyl acceptors and donors to form esters and amides (Tables S3 and S5; Figures 1 and S8). 5‐*O*‐esters are formed with shikimic and quinic acid. Small traces of 4‐*O*‐esters with shikimic acid can also be detected. Amides are formed with 3‐hydroxyanthranilic acid. Interestingly, the product formed with 5‐hydroxyanthranilic acid shows a different retention time and fragmentation pattern in comparison to the *p*‐coumaroyl‐*N*‐5‐hydroxyanthranilic acid reference substance suggesting the formation of an ester instead of an amide. l‐Threonic, 3‐aminobenzoic, 2,3‐dihydroxybenzoic, 2,5‐dihydroxybenzoic, and 3,4‐dihydroxybenzoic acids are accepted as well. Homogentisic acid, 1‐phenylethanol, and tryptamine are accepted weakly. Cinnamoyl‐CoA, *p*‐coumaroyl‐CoA, caffeoyl‐CoA, feruloyl‐CoA, sinapoyl‐CoA, and benzoyl‐CoA are all accepted with shikimic acid as acyl acceptor. NbHCT2 and NbHCT1 are sister enzymes and display 61.7% identity (Figure 3; Figure S5).

#### Biochemical characterization of NbHCT3

NbHCT3 forms 5‐*O*‐esters with quinic and shikimic acid and amides with 3‐hydroxyanthranilic acid. Additionally, 4‐*O*‐esters with shikimic acid are formed. 2,5‐Dihydroxybenzoic acid is also accepted and 1‐phenylethanol, homogentisic acid, and tryptamine very weakly (Figures 1 and S8; Tables S3 and S6). Cinnamoyl‐CoA, *p*‐coumaroyl‐CoA, caffeoyl‐CoA, feruloyl‐CoA, sinapoyl‐CoA, and benzoyl‐CoA are utilized with quinic acid as acyl acceptor. The optimal reaction conditions were measured at pH 7.47 and 37.6°C for the formation of *p*‐coumaroyl‐5‐*O*‐quinic acid (Figure S3; Table 3).

**Table 3:** Biochemical data for NbHCT3 (hydroxycinnamoyl‐CoA:quinic acid hydroxycinnamoyltransferase); *n* = 3 × 3 ± SEM. *k*_cat_ is calculated with the molar mass of the protein (including 6x‐His‐Tag) of 51.2 kDa

Kinetic data for	With constant substrate	*K*_m_ (μM)	*V*_max_ (mkat kg^−1^)	*k*_cat_ (sec^−1^)	*k*_cat_/*K*_m_ (L mol^−1^ sec^−1^)
Caffeoyl‐CoA^a^	Shikimic acid	168.97	0.741	0.0379	224.35
Shikimic acid^a^	Caffeoyl‐CoA	233 975.01	0.713	0.0365	0.16
*p*‐Coumaroyl‐CoA^a^	Shikimic acid	176.87	0.674	0.0345	195.11
Shikimic acid^a^	*p*‐Coumaroyl‐CoA	146 798.21	0.460	0.0235	0.16
Caffeoyl‐CoA	Quinic acid	11.53 ± 1.31	8.476 ± 0.585	0.4338 ± 0.0299	38 010.22 ± 2111.56
Quinic acid	Caffeoyl‐CoA	1751.67 ± 109.48	5.428 ± 0.245	0.2778 ± 0.0125	159.69 ± 10.95
*p*‐Coumaroyl‐CoA	Quinic acid	34.06 ± 4.19	11.880 ± 1.578	0.6080 ± 0.0808	17 801.71 ± 494.15
Quinic acid	*p*‐Coumaroyl‐CoA	4550.67 ± 2166.63	22.977 ± 1.699	1.1759 ± 0.0870	260.95 ± 29.61
pH optimum	7.47		Temperature optimum	37.6°C	

^a^ *n* = 3.

Substrate saturation curves for acyl acceptors and donors were recorded for caffeoyl‐5‐*O*‐shikimic, *p*‐coumaroyl‐5‐*O*‐shikimic, caffeoyl‐5‐*O*‐quinic, and *p*‐coumaroyl‐5‐*O*‐quinic acids (Figure 2c,d; Figure S6; Table 3). Substrate saturation could not be achieved for shikimic acid as acyl acceptor, and the data were therefore evaluated using the method of Cornish‐Bowden and Eisenthal (1978). Comparing the *K*_m_ values for shikimic acid with constant *p*‐coumaroyl‐CoA (*K*_m_ 146.8 mM) and caffeoyl‐CoA (*K*_m_ 234.0 mM) against the values for quinic acid with constant *p*‐coumaroyl‐CoA (*K*_m_ 4.6 mM) and caffeoyl‐CoA (*K*_m_ 1.8 mM) shows that quinic acid is strongly preferred. The turnover numbers for these acceptors also show the preference for quinic acid (1.18 sec^−1^ with *p*‐coumaroyl‐CoA and 0.28 sec^−1^ with caffeoyl‐CoA) over shikimic acid (0.02 sec^−1^ with *p*‐coumaroyl‐CoA and 0.04 sec^−1^ with caffeoyl‐CoA). This results in a 1600‐fold higher catalytic efficiency for quinic acid with *p*‐coumaroyl‐CoA (260.95 L mol^−1^ sec^−1^) over shikimic acid (0.16 L mol^−1^ sec^−1^) and in a 1000‐fold higher catalytic efficiency with caffeoyl‐CoA (159.69 L mol^−1^ sec^−1^ with quinic acid versus 0.16 L mol^−1^ sec^−1^ with shikimic acid). The *K*_m_ values for *p*‐coumaroyl‐CoA (34.06 μM) and caffeoyl‐CoA (11.53 μM) with shikimate differ only slightly, just as their turnover numbers (0.61 sec^−1^ for *p*‐coumaroyl‐CoA and 0.43 sec^−1^ for caffeoyl‐CoA). Taking together, the catalytic efficiency for *p*‐coumaroyl‐CoA (17 801.71 L mol^−1^ sec^−1^) is only half of the catalytic efficiency for caffeoyl‐CoA (38 010.22 L mol^−1^ sec^−1^). At low acyl donor concentrations, caffeoyl‐CoA is preferred because of the lower *K*_m_, while at higher acyl donor concentrations, the higher turnover number of *p*‐coumaroyl‐CoA outperforms caffeoyl‐CoA. In contrast to NbHCT1 and NbHCT2, NbHCT3 qualifies to be a hydroxycinnamoyl‐CoA: quinate HCT (HQT).

#### Biochemical characterization of NbHCT8

NbHCT8 is the most promiscuous enzyme described here, accepting the broadest range of acyl acceptors (Figure 1; Tables S3 and S7). It forms 5‐*O*‐esters with shikimic and quinic acid, 4‐*O*‐esters with shikimic acid (traces), amides with 3‐hydroxyanthranilic, 5‐hydroxyanthranilic, and 3‐aminobenzoic acids, and various esters with 2,3‐dihydroxybenzoic acid, 2,5‐dihydroxybenzoic acid, and 3,4‐dihydroxybenzoic acid. Both d‐ and l‐threonic acid were accepted with a strong preference for l‐threonic acid. Tryptamine, homogentisic acid, and 1‐phenylethanol are also converted, but the latter two very weakly. Cinnamoyl‐CoA, *p*‐coumaroyl‐CoA, caffeoyl‐CoA, feruloyl‐CoA, sinapoyl‐CoA, and benzoyl‐CoA are accepted with 3‐hydroxyanthranilic acid as acyl acceptor. The highest activity was measured at pH 6.53 and 39.9°C for the formation of *p*‐coumaroyl‐5‐*O*‐shikimic acid (Figure S3; Table 4).

**Table 4:** Biochemical data for NbHCT8 (hydroxycinnamoyl‐CoA:3‐hydroxyanthranilic acid hydroxycinnamoyltransferase); *n* = 3 × 3 ± SEM. *k*_cat_ is calculated with the molar mass of the protein (including 6x‐His‐Tag) of 71.7 kDa

Kinetic data for	With constant substrate	*K*_m_ (μM)	*V*_max_ (mkat kg^−1^)	*k*_cat_ (sec^−1^)	*k*_cat_/*K*_m_ (L mol^−1^ sec^−1^)
Caffeoyl‐CoA	Shikimic acid	6.00 ± 1.03	0.625 ± 0.017	0.0448 ± 0.0012	7884.96 ± 1226.49
Shikimic acid	Caffeoyl‐CoA	2329.33 ± 153.17	1.192 ± 0.030	0.0855 ± 0.0021	37.09 ± 3.11
*p*‐Coumaroyl‐CoA	Shikimic acid	19.07 ± 1.09	1.527 ± 0.086	0.1096 ± 0.0062	5803.06 ± 585.38
Shikimic acid^a^	*p*‐Coumaroyl‐CoA	1027.33 ± 67.14	1.632 ± 0.082	0.1170 ± 0.0059	114.47 ± 6.34
*p*‐Coumaroyl‐CoA	Quinic acid	10.53 ± 0.61	0.535 ± 0.013	0.0384 ± 0.0009	3661.43 ± 129.41
Quinic acid	*p*‐Coumaroyl‐CoA	54 753.33 ± 13 037.17	0.485 ± 0.063	0.0348 ± 0.0045	0.74 ± 0.21
*p*‐Coumaroyl‐CoA	3‐Hydroxyanthranilic acid	3.51 ± 0.24	1.512 ± 0.070	0.1085 ± 0.0050	31 302.52 ± 380.54
3‐Hydroxyanthranilic acid	*p*‐Coumaroyl‐CoA	307.60 ± 10.39	1.496 ± 0.063	0.1073 ± 0.0045	350.59 ± 25.01
pH optimum	6.53		Temperature optimum	39.9°C	

^a^ Substrate inhibition with shikimic acid at concentrations exceeding 8 mM.

Substrate saturation curves for acyl acceptors and donors were recorded for caffeoyl‐5‐*O*‐shikimic, *p*‐coumaroyl‐5‐*O*‐shikimic, *p*‐coumaroyl‐5‐*O*‐quinic, and *p*‐coumaroyl‐*N*‐3‐hydroxyanthranilic acids (Figure 2e,f; Figure S7; Table 4). The *K*_m_ value for quinic acid (54.8 mM) with *p*‐coumaroyl‐CoA is significantly higher than the values for shikimate (1.0 mM) and 3‐hydroxyanthranilate (0.3 mM). The same is true for the turnover numbers with constant *p*‐coumaroyl‐CoA concentrations (0.03 sec^−1^ for quinic acid versus 0.12 sec^−1^ for shikimic acid versus 0.11 sec^−1^). Taking together, the catalytic efficiency for 3‐hydroxyanthranilic acid with *p*‐coumaric acid (350.59 L mol^−1^ sec^−1^) is the highest followed by shikimic acid (114.47 L mol^−1^ sec^−1^) and quinic acid (0.74 L mol^−1^ sec^−1^). For shikimic acid with *p*‐coumaroyl‐CoA, a substrate inhibition could be observed, with 8 mM shikimic acid being the concentration of maximal observable velocity. The *K*_m_ and *k*_cat_ values were calculated on the rising part of the substrate saturation curve; therefore, the values are only applicable for concentrations lower than 8 mM. The catalytic efficiencies for *p*‐coumaroyl‐CoA with 3‐hydroxyanthranilic acid (31 302.52 L mol^−1^ sec^−1^) and shikimic acid (5803.06 L mol^−1^ sec^−1^) demonstrate the superiority of 3‐hydroxyanthranilic acid as acceptor substrate. As there was no reference substance for caffeoyl‐*N*‐3‐hydroxyanthranilic acid available in our laboratory, we compared the results for acyl donors using shikimic acid as the acyl acceptor. The affinity for caffeoyl‐CoA (*K*_m_ 6.0 μM) is three times higher than for *p*‐coumaroyl‐CoA (*K*_m_ 19.1 μM) with shikimic acid. The turnover number for caffeoyl‐CoA (0.04 sec^−1^) is only about half that of *p*‐coumaroyl‐CoA (0.11 sec^−1^) with shikimic acid, resulting in only slightly different catalytic efficiencies for caffeoyl‐CoA (7884.96 L mol^−1^ sec^−1^) and *p*‐coumaroyl‐CoA (5803.06 L mol^−1^ sec^−1^) with shikimic acid. *p*‐Coumaroyl‐CoA and caffeoyl‐CoA are equally well accepted.

#### Biochemical characterization of NbHCT5

NbHCT5 shows a high turnover with tryptamine (Figure 1; Tables S3 and S8), which makes it the only enzyme characterized that accepts a positively charged acyl acceptor. Homogentisic acid and 1‐phenylethanol were accepted, but the resulting peaks were weak. Cinnamoyl‐CoA, *p*‐coumaroyl‐CoA, caffeoyl‐CoA, feruloyl‐CoA, sinapoyl‐CoA, and benzoyl‐CoA are incorporated into the corresponding products with tryptamine.

#### Biochemical characterization of NbHCT4, NbHCT6, NbHCT7, and NbHCT9

NbHCT4, NbHCT6, NbHCT7, and NbHCT9 all accept homogentisic acid, tryptamine, and 1‐phenylethanol together with *p*‐coumaroyl‐CoA as acyl donor to a very low extent (Table S3; Tables S9–S12; Figure 1). None of the 70 acyl acceptor substrates, except the three mentioned above, resulted in sufficient turnover; thus, no further characterization was carried out.

### Phylogenetic analysis

Figure 3 shows the results of a phylogenetic analysis of the BAHD sequences found in *N. brasiliense* and already characterized BAHD acyltransferases from other plant species with 1000 bootstrap replicates. Three chloramphenicol acetyltransferases from *E. coli*, a suggested origin of BAHD acyltransferases (Moghe et al., 2023), were used to root the tree. All used sequences are listed in Table S13. The clade numbering follows Kruse et al. (2022).

NbHCT1, NbHCT2, and NbHCT8 all accepting shikimic, quinic, and 3‐hydroxyanthranilic acids are nested within the shikimate subclade of clade Va. From these three enzymes, NbHCT8 showed the highest substrate promiscuity. NbHCT3 (preferring the acceptor quinic acid) was sorted into clade V. It is sorted as the outmost group with a bootstrap value of 77 for clade V and NbHCT3. The location of NbHCT3 in the phylogenetic tree is especially interesting given that it does not cluster with already characterized hydroxycinnamoyl‐CoA:quinate HCTs. These two observations could be attributed to the fact that the classification for BAHDs only included a small number of sequences from non‐seed plants as well as to a putative lineage‐specific duplication in non‐seed plants (Moghe et al., 2023). An additional phylogenetic analysis was performed to further evaluate the placement of NbHCT3 (Figure S10) across all plants and showed that only the leptosporangiate fern orders Cyatheales and Polypodiales contain orthologs of NbHCT3. Following the classification of Christenhusz and Chase (2014) the mentioned orders are monophyletic, which suggests that NbHCT3 is part of a lineage‐specific duplication in leptosporangiate ferns. A differing classification by Nitta et al. (2022) would include Salviniales together with the mentioned two to make them monophyletic. NbHCT6 and NbHCT7 have a high sequence identity (78.9%) and have no homolog in the transcriptome of *S. spicant*; they are part of clade VI (bootstrap value: 41), a clade with members accepting various acceptor substrates, among them ω‐hydroxy fatty acids (Moghe et al., 2023). No preferred substrate could be detected for either BAHD acyltransferase. NbHCT5 was not sorted into a clade and is only loosely connected to clades V + VI (bootstrap value: 4). This is interesting because of all substrates tested by us, only tryptamine was accepted by NbHCT5. Further analysis was conducted, and orthologs in Lycophyta, Bryophyta, Marchantiophyta, Anthocerotophyta, and Polypodiophyta could be found. This analysis revealed that NbHCT5 is closer to clade IV and probably part of it (Figure S11). NbHCT4 belongs to clade IV (with members catalyzing aliphatic amine acylation), and NbHCT9 is part of clade I (bootstrap value: 34) encompassing BAHDs involved in anthocyanin/flavonoid/phenolic glucoside acylation. Although these enzymes accepted homogentisic acid, 1‐phenylethanol, and tryptamine, other acyl acceptors not tested by us are more probable to be the main substrates.

## DISCUSSION

The occurrence of RA and chlorogenic acid in *N. brasiliense* (syn. *B. brasiliense*) was for the first time reported by Harborne (1966) and later also by other researchers (Andrade et al., 2016; Bohm, 1968; Szabo et al., 1993). In a previous study, we detected 0.60% of the dry weight caffeoyl‐5‐*O*‐quinic acid (chlorogenic acid) and 0.27% of the dry weight RA in fern fronds of *N. brasiliense* together with other related hydroxycinnamoyl derivatives (Ufland & Petersen, 2026). Since then, we were interested in the HCTs in *N. brasiliense* participating in the biosynthesis of these esters. Since no genomic or transcriptomic information about this species is available, we used RACE‐PCR to amplify HCT sequences from *N. brasiliense*. A close relative with a publicly accessible transcriptome (1kp database), *S. spicant* (syn. *B. spicant*), was used to generate suitable RACE‐PCR primers. Thus, a total of nine HCTs could be amplified. Full‐length coding sequences were heterologously expressed in *E. coli*. The translated amino acid sequences were then, together with other BAHD acyltransferases, phylogenetically mapped. HCTs from clade V (NbHCT1, NbHCT2, NbHCT3, NbHCT8) are particularly interesting because all to date characterized HST, HQT, and RAS sequences are nested in this clade. We comprehensively recorded kinetic data for NbHCT1, NbHCT3, and NbHCT8 along with their temperature and pH optima. These and all other investigated BAHDs accepted cinnamoyl‐CoA, *p*‐coumaroyl‐CoA, caffeoyl‐CoA, feruloyl‐CoA, sinapoyl‐CoA, and benzoyl‐CoA as acyl donors (tested with the main substrate only) on the condition that we could identify an acceptor substrate.

The most efficient HCT from *N. brasiliense* is NbHCT3 (Figure 2; Figure S6; Table 3) with a catalytic efficiency around 38 000 L mol^−1^ sec^−1^ with caffeoyl‐CoA and quinic acid as substrates, thus forming caffeoyl‐5‐*O*‐quinate. Whereas the maximal velocity is about the same, the affinity toward caffeoyl‐CoA is three times lower than for *p*‐coumaroyl‐CoA. Comparing the acyl acceptors, quinate with a catalytic efficiency of around 160 L mol^−1^ sec^−1^ with caffeoyl‐CoA is accepted 1000 times better than shikimate (0.16 L mol^−1^ sec^−1^ with caffeoyl‐CoA). Other less accepted substrates were l‐ and d‐threonic acid with preference for l‐threonic acid, 2,3‐dihydroxybenzoic acid, 2,5‐dihydroxybenzoic acid, 3‐hydroxyanthranilic acid (forming an amide), homogentisic acid, 1‐phenylethanol, and tryptamine. NbHCT3 was, therefore, annotated as a HQT which could be responsible for the direct formation of chlorogenic acid (caffeoyl‐5‐*O*‐quinic acid) in *N. brasiliense*. All other HCTs also accepted quinic acid, but preferred shikimic acid over quinic acid. Until now all HCTs that preferred quinate over shikimate were allocated in a quinate subclade of clade Va. Interestingly, NbHCT3 is not clustering with these sequences in the phylogenetic tree, but rather is placed in an outgroup of clade V. The bootstrap value (77) of clade V and NbHCT3 supports the assumption that NbHCT3 is still part of clade V. Our study suggests that NbHCT3 is a gene of a lineage‐specific duplication event happening in the last common ancestor of Cyatheales and Polypodiales, as orthologs could only be found in the mentioned orders. This suggests an independent evolution of HQTs in ferns of the orders Cyatheales and Polypodiales. Other fern‐exclusive orthologs could not be found.

The high substrate promiscuity observed for NbHCT3 and all other characterized NbHCTs is a commonly observed feature for HCTs (up to 27 different acyl acceptors). Most HCTs reported in literature have more than one donor and acceptor substrate each (Moghe et al., 2023). The promiscuity of NbHCTs could be explained by the broad range of tested acyl acceptors, of which some are known to be present in selected species (e.g., 5‐hydroxyanthranilic acid in *Avena sativa*, Li et al., 2019) or unknown to plants, the chemical similarity of some of the substrates (e.g., four different dihydroxybenzoic acids), or the long incubation times with high amounts of substrates and enzyme.

NbHCT1, also belonging to clade V, shows similar catalytic efficiencies for *p*‐coumaroyl‐CoA with either shikimic or 3‐hydroxyanthranilic acid (around 3500 L mol^−1^ sec^−1^). This is also true when comparing the catalytic efficiencies of the acceptors with *p*‐coumaroyl‐CoA (around 115 L mol^−1^ sec^−1^). The catalytic efficiency for caffeoyl‐CoA is around 10 times lower than for *p*‐coumaroyl‐CoA and for quinic acid around 230 times lower than for shikimic or 3‐hydroxyanthranilic acid, making *p*‐coumaroyl‐5‐*O*‐shikimate and *p*‐coumaroyl‐*N*‐3‐hydroxyanthranilate the most efficiently formed products. To our knowledge, there are no 3‐hydroxyanthranilic or hydroxycinnamoyl‐*N*‐3‐hydroxyanthranilic acid derivatives found in Blechnaceae so far, also not in our own study (Ufland & Petersen, 2026). Therefore, it is rather unlikely that 3‐hydroxyanthranilic acid is a natural substrate for NbHCT1. Considering this, we annotate NbHCT1 as a HST. NbHCT1 shows a high sequence similarity with NbHCT2 mainly differing in NbHCT1 having a longer connecting loop between the two domains with a high content of histidine residues. It seems likely that NbHCT2 is just like NbHCT1 as a HST and acceptance of shikimate has been demonstrated by us, although a full kinetic characterization has not been done. This is further supported by the fact that both enzymes are in the shikimate subclade of clade V. Other accepted substrates by NbHCT1 and 2 were l‐ and d‐threonic acids (with preference for l‐threonic acid), 3‐aminobenzoic, 2,3‐dihydroxybenzoic, 2,5‐dihydroxybenzoic, 3,4‐dihydroxybenzoic, 3‐hydroxyanthranilic (forming an amide), 5‐hydroxyanthranilic, homogentisic acids, 1‐phenylethanol and tryptamine. Interestingly, NbHCT1 forms an amide with 5‐hydroxyanthranilic acid (proven with help of a commercial standard, Figure 1), but NbHCT2 forms a product with the same mass but a different retention time, suggesting that NbHCT2 forms not an amide but an ester with 5‐hydroxyanthranilic acid (no commercial standard available).

NbHCT8, annotated as hydroxycinnamoyl‐CoA:3‐hydroxyanthranilic acid HCT, also belongs to clade V and has the highest catalytic efficiency for *p*‐coumaroyl‐CoA with 3‐hydroxyanthranilic acid (around 31 000 L mol^−1^ sec^−1^). Comparing the catalytic efficiencies for *p*‐coumaroyl‐CoA with shikimic acid, the value is around three times lower, and the catalytic efficiency for quinic acid is even 500 times lower. The catalytic efficiency for caffeoyl‐CoA in comparison to *p*‐coumaroyl‐CoA is roughly the same. As hydroxycinnamoyl amides with 3‐hydroxyanthranilic acid have not been found in *N. brasiliense*, it must be assumed that this is an intrinsic side activity of HCTs using shikimic and quinic acids as main substrates. Similar observations have already been made for HSTs and HQTs from other plants (Bömeke & Petersen, 2025; Ernst et al., 2022; Sander & Petersen, 2011). NbHCT8 is sorted into a subclade of clade Va together with enzymes with a broad acceptor range. This correlates to our observation that NbHCT8 is the most promiscuous regarding acyl acceptors. Besides the acyl acceptors mentioned above, l‐ and d‐threonic acids (with preference for l‐threonic acid), 3‐aminobenzoic, 2,3‐dihydroxybenzoic, 2,5‐dihydroxybenzoic, 3,4‐dihydroxybenzoic, 5‐hydroxyanthranilic, homogentisic acids, 1‐phenylethanol, and tryptamine were accepted ranging from good to low activities as shown in Figure 1.

NbHCT5 catalyzes the formation of tryptamine amides with various hydroxycinnamoyl derivatives to a high extent. A full kinetic characterization was not possible due to the unavailability of commercial standards. Due to the intense mass peaks (6.3 × 10^5^ counts for *p*‐coumaroyl‐*N*‐tryptamine) and no other identifiable acyl acceptors, we suggest an annotation as hydroxycinnamoyl‐CoA:tryptamine HCT. NbHCT5 forms an ortholog group with genes from Lycophyta, Bryophyta, Marchantiophyta, Anthocerotophyta, and Polypodiophyta. The bootstrap value (4) for NbHCT5 and clade V or VI does not support a decision, yet a closer look into the ortholog group (Figure S11) revealed that NbHCT5 is more closely related to clade IV, a clade that includes members known for acceptance of aliphatic amines. One explanation for the ambiguous inclusion in a BAHD clade could be the underrepresentation of non‐seed plant sequences in Figure 3. Tryptamine as main acceptor substrate has previously been described for a number of HCTs from *Oryza sativa* and *Hordeum vulgare* involved in the formation of phenolamide phytoalexins (Peng et al., 2016; Ube et al., 2019).

All expressed NbHCTs accepted a variety of acyl acceptors (e.g. 1‐phenylethanol) to a very low extent. We suggest that, because of the small size, these acceptors fit into the catalytic site but are not turned over quickly because these acceptors may not be stabilized in the active site. Moreover, in order to detect even low activities, high concentrations of substrates and enzyme were incubated for a long time. Low levels of basic activity were detectable for NbHCT4, 6, 7, and 9, but product formation remained at background levels with all 70 tested acyl acceptor substrates. NbHCT4 is part of clade IV. NbHCT6 and NbHCT7 are located in clade VI (bootstrap value: 41) and NbHCT9 is part of clade I (bootstrap value: 34). Here, further research is needed.

From a physiological point of view, it is interesting that the fern *N. brasiliense* already has redundant enzymes, for example, NbHCT1, 2, and 8 forming hydroxycinnamoylshikimic acid esters. These HSTs might be expressed at different levels in different tissues and developmental stages of *N. brasiliense* and explain the occurrence of multiple HSTs, which should be further investigated in future. BAHD acyltransferases might have played an important role in the evolution of plants by being responsible for the formation of various specialized metabolites with potent biological activities such as UV screens, pathogen, and herbivore deterrents or as stabilizing components; thus, ferns could be seen as transitional in this process. As ferns have lignified vessels, the formation of lignin monomers (monolignols), especially caffeyl, guaiacyl, and syringyl moieties, probably is formed by the same biochemical pathway via hydroxycinnamoylshikimic acid involving HST as in gymnosperms and angiosperms (Boerjan et al., 2003). The number of BAHD acyltransferase copies increased from only a few in algae to 50–200 in angiosperms. Two ferns investigated with this respect, *S. cucullata* and *A. filiculoides*, play an intermediate role containing less than 20 BAHD copies (Moghe et al., 2023). Gene duplication can lead to genetic redundancy, neo‐ or sub‐functionalization, or gene loss and may therefore be an important step in evolutionary diversification in ferns.

In conclusion, the expressed sequences of nine BAHD acyltransferases were isolated from *N. brasiliense*, a fern species accumulating RA and chlorogenic acid, and successfully expressed in *E. coli*. All enzymes were shown to act as HCTs with some of the 70 substrates tested in enzyme assays, although in some cases, only traces of products could be detected. Three of these enzymes were fully kinetically characterized. NbHCT1 is presumably involved in the formation of *p*‐coumaroyl‐5‐*O*‐shikimic acid in *N. brasiliense*, an important intermediate in the formation of C‐, G‐, and S‐monolignols and ultimately lignin. NbHCT3 is presumably involved in the formation of caffeoyl‐5‐*O*‐quinic acid (chlorogenic acid). Shikimic and quinic acid esters are formed by NbHCT8 as well, although the highest efficiency of this enzyme was observed for 3‐hydroxyanthranilic acid. Tryptamine amides were recorded as reaction products of NbHCT5. The enzyme, however, responsible for the formation of RA or its precursors has not yet been found in *N. brasiliense*. Further research into acyltransferases in ferns and especially in *N. brasiliense* is therefore required.

## MATERIALS AND METHODS

### Primers, sequencing, and gene synthesis

Commercial services were used for primer synthesis (Eurofins Genomics Germany), sequencing (Microsynth Seqlab), and gene synthesis (BioCat). All commercial kits were utilized in accordance with the instructions outlined in the manufacturers' manuals.

### Acquisition of plant material

Plant material of *N. brasiliense* (Desv.) Gasper & V.A.O. Dittrich (accession number: 1977/388) was obtained from the Botanical Garden, Marburg University, Germany, frozen in liquid nitrogen, and freeze‐dried overnight. The dry pinnae were stored at −80°C until further use.

### Total RNA extraction and 5′/3′‐RACE cDNA synthesis

RNA extraction was performed according to the protocol described by Pearson et al. (2006) with the final RNA precipitation overnight at 4°C. 5′/3′‐RACE cDNA was synthesized using the SMARTer® RACE 5′/3′ Kit (Takara).

### 5′/3′‐RACE primer design and amplification of genes

To obtain sequences for BAHD acyltransferases in *N. brasiliense* (Blechnaceae), the transcriptome of *S. spicant* (L.) Roth (formerly known as *B. spicant*, Blechnaceae) from the 1kP database (taxid: 114457, sample code: VITX) was searched using the BLASTP algorithm with the protein sequence of RAS from *C. blumei* Benth. as query (UniProt A0PDV5; Berger et al., 2006). The results are shown in Table S1.

To receive the full homologous sequences from *N. brasiliense*, 5′/3′‐RACE‐PCR was conducted for all genes, except *NbHCT1*. *NbHCT1* full‐length primers were designed directly on the homolog of *S. spicant* (scaffold 2007085). For *NbHCT2* (scaffold 2011375), *NbHCT4* (scaffold 2009524), *NbHCT5* (scaffold 2014301), *NbHCT8* (scaffold 2099633), and *NbHCT9* (scaffold 2003763), 5′/3′‐RACE primers were designed on GC‐rich regions of the homologous *S. spicant* sequences. For *NbHCT3, NbHCT6*, and *NbHCT7*, the 3′‐RACE primers were based on a degenerated DFGWG motif and scaffold 2011306 (*NbHCT3*) as the 5′ end. The sequences of the 5′‐ and 3′‐RACE amplicons were used to design primers for full‐length amplification. Restriction sites or 19 bp‐sequences complementary to the expression plasmid pET‐15b (Novagen) were added to full‐length primers for insertion into the plasmid (for primer sequences and PCR protocols, see Table S14). The codon‐optimized coding sequence of *NbHCT1* was obtained commercially (BioCat). After agarose (0.7%) gel electrophoresis, bands were cut out and eluted using NucleoSpin columns (Machery‐Nagel). The same workflow was followed for restriction enzyme‐digested pET‐15b for the subsequent construction of expression vectors.

### Construction of sequencing and expression vectors

All 5′/3′‐RACE‐PCR products were ligated into pDRIVE (Qiagen), introduced into chemically competent *E. coli* EZ cells (Qiagen) and plated out on blue‐white‐screening medium (LB‐agar; Lessard, 2013) with 0.1 mg ml^−1^ ampicillin, 0.08 mg ml^−1^ 5‐bromo‐4‐chloro‐3‐indoxyl‐β‐d‐galactopyranoside (X‐Gal), and 50 μM isopropyl‐β‐d‐thiogalactoside (IPTG). Plasmids were extracted from overnight‐incubated single white colonies with the QIAprep Spin Miniprep Kit (Qiagen) and sequenced.

Amplified full‐length sequences were introduced into pET‐15b either by ligation with T4 DNA ligase (Thermo Fisher Scientific; *NbHCT1*, *3*, *7*) into restriction sites and/or by homologous recombination (*NbHCT2*, *4*, *5*, *6*, *8*, *9*). Homologous recombination was performed by combining 200 ng restriction enzyme‐digested and purified pET‐15b vector with 400 ng of the amplified and purified full‐length‐PCR product and 70 μl chemically competent *E. coli* EZ cells for 30 min on ice, followed by a heat shock for 90 sec at 42°C. After the addition of 140 μl SOC medium (Lessard, 2013), the cells were incubated for 45 min at 37°C. Transformants were plated out on LB‐agar with 0.1 mg ml^−1^ ampicillin and incubated overnight at 37°C. Plasmids were then extracted from overnight‐incubated liquid cultures of single colonies with the QIAprep Spin Miniprep Kit (Qiagen) and sequenced. Chemically competent *E. coli* SoluBL21 (Amsbio) were then transformed with pET‐15b harboring the sequences of interest. Plasmids were extracted again and sequenced for sequence verification. Glycerol stocks were prepared and kept at −80°C.

### Heterologous expression of NbHCTs in *E. coli* SoluBL21

TB medium (Lessard, 2013) with 0.1 mg ml^−1^ ampicillin was inoculated with an overnight stock of *E. coli* SoluBL21 pET‐15b harboring one of the sequences of *NbHCT1‐9* and incubated at 37°C at 180 rpm until an OD_600_ of 0.4–0.6. Expression was then induced with 1 mM IPTG and the cultures further cultivated for 16 h at 25°C at 160 rpm. Cells were centrifuged at 3000 ***g*** at 4°C for 10 min and the medium was discarded. The cell pellet was frozen in liquid nitrogen and thawed on ice with 4 mL 50 mM potassium phosphate buffer (KP_i_) pH 8.0 per gram of bacterial pellet. The pellet was resuspended and a spatula tip of lysozyme added, followed by incubation on ice for 30 min. The suspension was ultrasonicated four times for 30 sec at 0.5 cycles and 100% amplitude on ice with intermediate 30 sec on ice. Residues were sedimented at 10 000 ***g*** at 4°C for 10 min. The supernatant (crude extract) was decanted.

### His‐tag purification and desalting of heterologously expressed NbHCTs

One milliliter of ROTI®Garose‐His/Ni Beads (Carl Roth) in a disposable column was equilibrated with 50 mM KP_i_, 300 mM NaCl, 10 mM imidazole, pH 8.0 buffer. The crude extract was adjusted to 300 mM NaCl, 10 mM imidazole and incubated with the His/Ni beads on ice for 1 h under shaking. The flow‐through was discarded. The matrix was washed thrice with 2 ml 50 mM KP_i_, 300 mM NaCl, 20 mM imidazole, pH 8.0 buffer each, and the recombinant 6xHis‐tagged protein eluted with 3× 1 ml 50 mM KP_i_, 300 mM NaCl, 250 mM imidazole, pH 8.0 buffer. Prepacked PD‐10 columns (Cytiva) were equilibrated with 25 ml 100 mM KP_i_ pH 7.0 buffer. The PD‐10 column was then loaded with 2.5 ml of the pooled elution fractions and eluted with 3.5 ml 100 mM KP_i_ buffer pH 7.0. The protein concentration was quantified as described by Bradford (1976) using bovine serum albumin (1 mg ml^−1^) as reference. Recombinant proteins were stored at −20 °C. An empty vector control carrying an empty pET‐15b was treated equally.

The molecular masses and isoelectric points of the translated amino acid sequences of NbHCT1‐9 were calculated using the ProtParam tool (https://web.expasy.org/protparam/).

### SDS‐PAGE and western blot analysis of heterologously expressed NbHCTs

SDS‐PAGE was essentially conducted according to Laemmli (1970) using 20 μl and/or 20 μl 10× concentrated, TCA‐precipitated protein solution per lane. Western blot analysis was performed according to Mahmood and Yang (2012) but using a discontinuous buffer system with an Immobilon‐P membrane as described in the user manual (https://www.sigmaaldrich.com/deepweb/assets/sigmaaldrich/product/documents/159/578/ipvh85rug‐mk). Mouse anti‐His_6_‐Tag monoclonal antibodies (Invitrogen, Life Technologies; MA1‐21315) were used as the primary antibody, followed by goat anti‐mouse IgG Fc, alkaline phosphatase conjugate (Life Technologies; A16087) as the secondary antibody. Bound alkaline phosphatase activity was visualized with nitro blue tetrazolium chloride/5‐bromo‐4‐chloro‐3‐indolyl‐phosphate following standard protocols (https://www.sysy.com/protocols/blot.php). Blots of NbHCTs and the empty vector control are depicted in Figure S2.

### Enzyme assays for substrate search

A total of 70 putative acyl acceptor substrates and seven acyl donor substrates were tested (Table S2). Assays consisted generally of 5 μl 2.5 mM donor, 20 μl acceptor in varying concentrations, 100 μl of 100 mM KP_i_ pH 7.0 containing approximately 5 μg of each NbHCT. The assays were incubated at 30°C for 3 h against empty vector controls. The assays were then, dependent on the acceptor substrate (Table S2), prepared for LC/ESI‐MS/MS analysis by either method A or B. For method A, the assays were stopped with 25 μl 6 N HCl and extracted with 500 μl ethyl acetate. The organic phase was evaporated, and the residues redissolved in 100 μl 10% acetonitrile, 0.1% formic acid, and centrifuged for 10 min at 16 000 ***g***. For method B, the assays were stopped with 25 μl 6 N HCl and 150 μl methanol. The precipitated protein was then sedimented at 16 000 ***g*** for 20 min and the supernatant subjected to analysis.

For analysis by LC/ESI‐MS/MS (HPLC: Agilent 1260 series, column: Multospher 120 RP 18, 5 μm, 250 × 2 mm; solvent A: 0.1% formic acid in water, solvent B: 0.1% formic acid in acetonitrile; 0–10 min: 5% B → 100% B, 10–15 min: 100% B, 15–15.1 min: 100% B → 5% B, 15.1–20 min → 5% B; flow rate: 0.5 ml min^−1^; mass spectrometer: micrOTOF‐Q III with ESI‐source, Bruker Daltonics, calibration with 5 mM Na‐formate), 10 μl (negative mode) or 5 μl (positive mode) was analyzed, dependent on the acceptor substrate (Table S2).

### Enzyme assays for biochemical characterization

#### pH and temperature optimum

pH and temperature optima for NbHCT1 (caffeoyl‐5‐*O*‐shikimic acid), NbHCT3 (*p*‐coumaroyl‐5‐*O*‐quinic acid), and NbHCT8 (*p*‐coumaroyl‐5‐*O*‐shikimic acid) were performed in 125 μl assays as described in Table S15. After stopping the enzyme reactions with 20 μl 6 N HCl, the assays were extracted twice with 500 μl ethyl acetate each. The combined ethyl acetate fractions were evaporated, and the residues redissolved in 100 μl 35% methanol, 0.01% *o*‐phosphoric acid and quantified by HPLC against an external standard of 0.5 nmol of the corresponding product.

#### Kinetic parameters

Kinetic parameters for NbHCT1, NbHCT3, and NbHCT8 were determined in 125 μl assays, following the conditions and compositions described in Table S16. Linear enzyme activity over the whole reaction time and a suitable range for the variable substrate was tested beforehand. The constant substrate was adjusted to a concentration approximately five times the corresponding *K*_m_, ensuring not to exert a negative effect on the velocity of the reaction. After stopping the enzyme assays with 20 μl 6 N HCl, the assays were extracted twice with 500 μl ethyl acetate. The combined ethyl acetate fractions were then evaporated, redissolved in 100 μl 35% methanol, 0.01% *o*‐phosphoric acid and quantified by HPLC against an external standard containing 0.5 nmol of the corresponding product. Kinetic parameters were determined with GraphPad Prism 10 for each triplicate, except for assays with NbHCT3 and shikimic acid as acyl acceptor substrate, where no substrate saturation could be attained. These data were evaluated according to Cornish‐Bowden and Eisenthal (1978).

### Phylogenetic analysis

Phylogenetic analysis was performed according to Kruse et al. (2022). The sequences were aligned with MAFFT 7.526 (Katoh et al., 2002) (parameters: *‐‐maxiterate 1000 –genafpair ‐‐thread 70*) and checked for correct alignment of the HxxxDG and DFGWG motifs. IQ‐Tree v1.6.10 (Nguyen et al., 2015) was then used to generate a phylogenetic tree (parameters: *‐st AA ‐nt AUTO ‐ntmax 70 ‐b 1000 ‐m LG+F+G4*). The resulting tree was visualized with iTOL version 7.2 (https://itol.embl.de/). The sequences included in the tree were taken from Kruse et al. (2022) and some own sequences added (Table S13).

To investigate the origin of *NbHCT3* and *NbHCT5* further, BLASTP searches in the total 1kp transcriptome with the respective protein sequences were conducted (Datasets A and C in Supporting Information datasets; https://db.cngb.org/onekp/, Carpenter et al., 2019; Leebens‐Mack et al., 2019). Sequences were aligned with Clustal Omega (Sievers et al., 2011) together with BAHD sequences (Table S13) and a phylogenetic tree created with Simple Phylogeny (EMBL‐EBI Job Dispatcher) (Madeira et al., 2024). After orthologous sequences of *NbHCT3* and *NbHCT5* were found, the process was redone excluding the plant species with orthologous sequences (Datasets B and D in Supporting information datasets).

## Accession Numbers

The native cDNA coding sequences of the enzymes characterized in this work were deposited in Genbank as: PV344582 – *NbHCT1*, PV344583 – *NbHCT2*, PV344584 – *NbHCT3*, PV344585 – *NbHCT4*, PV344586 – *NbHCT5*, PV344587 – *NbHCT6*, PV344588 – *NbHCT7*, PV344589 – *NbHCT8*, PV344590 – *NbHCT9*.

## CONFLICT OF INTEREST

The authors have no competing interests to declare that are relevant to the content of this article.

## Supporting information


**Figure S1.** Nomenclature for shikimic and quinic acid esters.
**Figure S2.** Western blot analysis.
**Figure S3.** pH and temperature optima of NbHCT1, NbHCT3, and NbHCT8.
**Figure S4.** Michaelis–Menten graphs for NbHCT1.
**Figure S5.** Amino acid sequence identity and similarity of NbHCT1‐9.
**Figure S6.** Michaelis–Menten graphs for NbHCT3.
**Figure S7.** Michaelis–Menten graphs for NbHCT8.
**Figure S8.** Extracted ion chromatograms (EIC) of substrate search assays.
**Figure S9.** MS/MS fragmentation of 3/4/5‐*O*‐shikimic/quinic acid esters.
**Figure S10.** Phylogenetic analysis of NbHCT3 orthologs.
**Figure S11.** Phylogenetic analysis of NbHCT5 orthologs.
**Table S1.** BLASTP search in the transcriptome of *Struthiopteris spicant*.
**Table S2.** Screened putative NbHCT substrates and method of preparation and detection.
**Table S3.** Investigated NBHCTs and their transformed acyl donors and acyl acceptors.
**Table S4.** Summary of identified products formed in NbHCT1 assays.
**Table S5.** Summary of identified products formed in NbHCT2 assays.
**Table S6.** Summary of identified products formed in NbHCT3 assays.
**Table S7.** Summary of identified products formed in NbHCT8 assays.
**Table S8.** Summary of identified products formed in NbHCT5 assays.
**Table S9.** Summary of identified products formed in NbHCT4 assays.
**Table S10.** Summary of identified products formed in NbHCT6 assays.
**Table S11.** Summary of identified products formed in NbHCT7 assays.
**Table S12.** Summary of identified products formed in NbHCT9 assays.
**Table S13.** Sequences used in the phylogenetic tree.
**Table S14.** PCR conditions and primers for amplification of *NbHCT1‐9*.
**Table S15.** Conditions, composition, and detection parameters for the determination of pH and temperature optima.
**Table S16.** Conditions, composition, and detection parameters for the determination of kinetic parameters.

**Dataset A.** BLASTP search of NbHCT3 in the 1kp database.
**Dataset B.** BLASTP search of NbHCT3 in the 1kp database excluding Cyatheales and Polypodiales.
**Dataset C.** BLASTP search of NbHCT5 in the 1kp database.
**Dataset D.** BLASTP search of NbHCT5 in the 1kp database excluding Lycophyta, Bryophyta, Marchantiophyta, Anthocerotophyta, and Polypodiaphyta.

## Data Availability

Data supporting the information given in the main manuscript are presented in the Supporting Information file.

## References

[tpj70837-bib-0001] Abrankó, L. & Clifford, M.N. (2017) An unambiguous nomenclature for the acyl‐quinic acids commonly known as chlorogenic acids. Journal of Agricultural and Food Chemistry, 65, 3602–3608. Available from: 10.1021/acs.jafc.7b00729 28420230

[tpj70837-bib-0002] Andrade, J.M.d.M. , Biegelmeyer, R. , Dresch, R.R. , Maurmann, N. , Pranke, P. & Henriques, A.T. (2016) *In vitro* antioxidant and enzymatic approaches to evaluate neuroprotector potential of *Blechnum* extracts without cytotoxicity to human stem cells. Pharmacognosy Magazine, 12, 171–177. Available from: 10.4103/0973-1296.186349 27601845 PMC4989790

[tpj70837-bib-0003] Berger, A. , Meinhard, J. & Petersen, M. (2006) Rosmarinic acid synthase is a new member of the superfamily of BAHD acyltransferases. Planta, 224, 1503–1510. Available from: 10.1007/s00425-006-0393-y 17047986

[tpj70837-bib-0004] Boerjan, W. , Ralph, J. & Baucher, M. (2003) Lignin biosynthesis. Annual Review of Plant Biology, 54, 519–546. Available from: 10.1146/annurev.arplant.54.031902.134938 14503002

[tpj70837-bib-0005] Bohm, B.A. (1968) Phenolic compounds in ferns—III. An examination of some ferns for caffeic acid derivatives. Phytochemistry, 7, 1825–1830. Available from: 10.1016/S0031-9422(00)86654-6

[tpj70837-bib-0006] Bömeke, P. & Petersen, M. (2025) Phenolic metabolism in *Sarcandra glabra* is mediated by distinct BAHD hydroxycinnamoyltransferases. The Plant Journal, 121, e70035. Available from: 10.1111/tpj.70035 40029908 PMC11875395

[tpj70837-bib-0007] Bradford, M.M. (1976) A rapid and sensitive method for the quantitation of microgram quantities of protein utilizing the principle of protein‐dye binding. Analytical Biochemistry, 72, 248–254. Available from: 10.1016/0003-2697(76)90527-3 942051

[tpj70837-bib-0008] Cao, H. , Chai, T.T. , Wang, X. , Morais‐Braga, M.F.B. , Yang, J.H. , Wong, F.C. et al. (2017) Phytochemicals from fern species: potential for medicine applications. Phytochemistry Reviews, 16, 379–440. Available from: 10.1007/s11101-016-9488-7 32214919 PMC7089528

[tpj70837-bib-0009] Carballo‐Sanchez, M.P. , Miguel‐Chávez, R.S. , Alarcón, A. & Ferrera‐Cerrato, R. (2022) Polyphenol characterization in *Azolla filiculoides* after drying and enzymatic hydrolysis processes. BioResources, 17, 2074–2083. Available from: 10.15376/biores.17.2.2074-2083

[tpj70837-bib-0010] Carpenter, E.J. , Matasci, N. , Ayyampalayam, S. , Wu, S. , Sun, J. , Yu, J. et al. (2019) Access to RNA‐sequencing data from 1,173 plant species: the 1000 plant transcriptomes initiative (1KP). GigaScience, 8, 1–7. Available from: 10.1093/gigascience/giz126 PMC6808545 31644802

[tpj70837-bib-0011] Christenhusz, M.J.M. & Chase, M.W. (2014) Trends and concepts in fern classification. Annals of Botany, 113, 571–594. Available from: 10.1093/aob/mct299 24532607 PMC3936591

[tpj70837-bib-0012] Clifford, M.N. , Johnston, K.L. , Knight, S. & Kuhnert, N. (2003) Hierarchical scheme for LC‐MSn identification of chlorogenic acids. Journal of Agricultural and Food Chemistry, 51, 2900–2911. Available from: 10.1021/jf026187q 12720369

[tpj70837-bib-0013] Cornish‐Bowden, A. & Eisenthal, R. (1978) Estimation of Michaelis constant and maximum velocity from the direct linear plot. Biochimica et Biophysica Acta, 523, 268–272. Available from: 10.1016/0005-2744(78)90030-X 629990

[tpj70837-bib-0014] D'Auria, J.C. (2006) Acyltransferases in plants: a good time to be BAHD. Current Opinion in Plant Biology, 9, 331–340. Available from: 10.1016/j.pbi.2006.03.016 16616872

[tpj70837-bib-0015] de Gasper, A.L. , Dittrich, V.A.D.O. , Smith, A.R. & Salino, A. (2016) A classification for Blechnaceae (Polypodiales: Polypodiopsida): new genera, resurrected names, and combinations. Phytotaxa, 275, 191–227. Available from: 10.11646/phytotaxa.275.3.1

[tpj70837-bib-0016] Ernst, L. , Wohl, J. , Bauerbach, E. & Petersen, M. (2022) Hydroxycinnamoyltransferase and CYP98 in phenolic metabolism in the rosmarinic acid‐producing hornwort *Anthoceros agrestis* . Planta, 255, 75. Available from: 10.1007/s00425-022-03856-9 35235057 PMC8891189

[tpj70837-bib-0017] Fasolo, J.M.M.A. , Vizuete, A.F.K. , Rico, E.P. , Rambo, R.B.S. , Toson, N.S.B. , Santos, E. et al. (2021) Anti‐inflammatory effect of rosmarinic acid isolated from *Blechnum brasiliense* in adult zebrafish brain. Comparative Biochemistry and Physiology, 239, 108874. Available from: 10.1016/j.cbpc.2020.108874 32805443

[tpj70837-bib-0018] Harborne, J.B. (1966) Caffeic acid ester distribution in higher plants. Zeit‐schrift für Naturforschung, 21b, 604–605. Available from: 10.1515/znb-1966-0634

[tpj70837-bib-0019] Hohlfeld, M. , Veit, M. & Strack, D. (1996) Hydroxycinnamoyltransferases involved in the accumulation of caffeic acid esters in gametophytes and sporophytes of *Equisetum arvense* . Plant Physiology, 111, 1153–1159. Available from: 10.1104/pp.111.4.1153 12226354 PMC160991

[tpj70837-bib-0020] Katoh, K. , Misawa, K. , Kuma, K. & Miyata, T. (2002) MAFFT: a novel method for rapid multiple sequence alignment based on fast Fourier transform. Nucleic Acids Research, 30, 3059–3066. Available from: 10.1093/nar/gkf436 12136088 PMC135756

[tpj70837-bib-0021] Kongsung, S. , Inthachat, W. , Chantong, B. , Suttisansanee, U. , On‐Nom, N. , Chupeerach, C. et al. (2024) Box‐Behnken design‐based optimization of phytochemical extraction from *Diplazium esculentum* (Retz.) Sw. Associated with its antioxidant and anti‐Alzheimer's properties. Molecules, 29, 2204. Available from: 10.3390/molecules29102204 38792065 PMC11124457

[tpj70837-bib-0022] Kriegshauser, L. , Knosp, S. , Grienenberger, E. , Tatsumi, K. , Gütle, D.D. , Sørensen, I. et al. (2021) Function of the HYDROXYCINNAMOYL‐CoA:SHIKIMATE HYDROXYCINNAMOYL TRANSFERASE is evolutionarily conserved in embryophytes. Plant Cell, 33, 1472–1491. Available from: 10.1093/plcell/koab044 33638637 PMC8254490

[tpj70837-bib-0023] Kruse, L.H. , Weigle, A.T. , Irfan, M. , Martínez‐Gómez, J. , Chobirko, J.D. , Schaffer, J.E. et al. (2022) Orthology‐based analysis helps map evolutionary diversification and predict substrate class use of BAHD acyltransferases. The Plant Journal, 111, 1453–1468. Available from: 10.1111/tpj.15902 35816116

[tpj70837-bib-0024] Laemmli, U.K. (1970) Cleavage of structural proteins during assembly of the head of bacteriophage T4. Nature, 227, 680–685. Available from: 10.1038/227680a0 5432063

[tpj70837-bib-0025] Landmann, C. , Hücherig, S. , Fink, B. , Hoffmann, T. , Dittlein, D. , Coiner, H.A. et al. (2011) Substrate promiscuity of a rosmarinic acid synthase from lavender (*Lavandula angustifolia* L.). Planta, 234, 305–320. Available from: 10.1007/s00425-011-1400-5 21424826

[tpj70837-bib-0026] Leebens‐Mack, J.H. , Barker, M.S. , Carpenter, E.J. , Deyholos, M.K. , Gitzendanner, M.A. , Graham, S.W. et al. (2019) One thousand plant transcriptomes and the phylogenomics of green plants. Nature, 574, 679–685. Available from: 10.1038/s41586-019-1693-2 31645766 PMC6872490

[tpj70837-bib-0027] Lessard, J.C. (2013) Growth media for *E. coli* . In: Lorsch, J. (Ed.) Methods in enzymology. San Diego: Academic Press, pp. 181–189. Available from: 10.1016/B978-0-12-420067-8.00011-8 24182923

[tpj70837-bib-0028] Levsh, O. , Pluskal, T. , Carballo, V. , Mitchell, A.J. & Weng, J.K. (2019) Independent evolution of rosmarinic acid biosynthesis in two sister families under the lamiids clade of flowering plants. The Journal of Biological Chemistry, 294, 15193–15205. Available from: 10.1074/jbc.RA119.010454 31481469 PMC6802498

[tpj70837-bib-0029] Li, Z. , Chen, Y. , Meesapyodsuk, D. & Qiu, X. (2019) The biosynthetic pathway of major avenanthramides in oat. Metabolites, 9, 163. Available from: 10.3390/metabo9080163 31394723 PMC6724135

[tpj70837-bib-0030] Ma, Q.H. (2024) Lignin biosynthesis and its diversified roles in disease resistance. Genes, 15, 295. Available from: 10.3390/genes15030295 38540353 PMC10969841

[tpj70837-bib-0031] Madeira, F. , Madhusoodanan, N. , Lee, J. , Eusebi, A. , Niewielska, A. , Tivey, A.R.N. et al. (2024) The EMBL‐EBI job dispatcher sequence analysis tools framework in 2024. Nucleic Acids Research, 52, W521–W525. Available from: 10.1093/nar/gkae241 38597606 PMC11223882

[tpj70837-bib-0032] Mahmood, T. & Yang, P.C. (2012) Western blot: technique, theory, and trouble shooting. North American Journal of Medical Sciences, 4, 429–434. Available from: 10.4103/1947-2714.100998 23050259 PMC3456489

[tpj70837-bib-0033] Moghe, G. , Kruse, L.H. , Petersen, M. , Scossa, F. , Fernie, A.R. , Gaquerel, E. et al. (2023) BAHD company: the ever‐expanding roles of the BAHD acyltransferase gene family in plants. Annual Review of Plant Biology, 74, 165–194. Available from: 10.1146/annurev-arplant-062922-050122 36450296

[tpj70837-bib-0034] Nguyen, L.‐T. , Schmidt, H.A. , Haeseler, A. & Minh, B.Q. (2015) IQ‐TREE: a fast and effective stochastic algorithm for estimating maximum‐likelihood phylogenies. Molecular Biology and Evolution, 32, 268–274. Available from: 10.1093/molbev/msu300 25371430 PMC4271533

[tpj70837-bib-0035] Nitta, J.H. , Schuettpelz, E. , Ramírez‐Barahona, S. & Iwasaki, W. (2022) An open and continuously updated fern tree of life. Frontiers in Plant Science, 13, 909768. Available from: 10.3389/fpls.2022.909768 36092417 PMC9449725

[tpj70837-bib-0036] Ogata, A. , Tsuruga, A. , Matsuno, M. & Mizukami, H. (2004) Elicitor‐induced rosmarinic acid biosynthesis in *Lithospermum erythrorhizon* cell suspension cultures: activities of rosmarinic acid synthase and the final two cytochrome P450‐catalyzed hydroxylations. Plant Biotechnology, 21, 393–396. Available from: 10.5511/plantbiotechnology.21.393

[tpj70837-bib-0037] Pearson, G. , Lago‐Leston, A. , Valente, M. & Serrão, E. (2006) Simple and rapid RNA extraction from freeze‐dried tissue of brown algae and seagrasses. European Journal of Phycology, 41, 97–104. Available from: 10.1080/09670260500505011

[tpj70837-bib-0038] Peng, M. , Gao, Y. , Chen, W. , Wang, W. , Shen, S. , Shi, J. et al. (2016) Evolutionarily distinct BAHD *N*‐acyltransferases are responsible for natural variation of aromatic amine conjugates in Rice. Plant Cell, 28, 1533–1550. Available from: 10.1105/tpc.16.00265 27354554 PMC4981135

[tpj70837-bib-0039] Petersen, M. (2013) Rosmarinic acid: new aspects. Phytochemistry Reviews, 12, 207–227. Available from: 10.1007/s11101-013-9282-8

[tpj70837-bib-0040] Petersen, M. , Abdullah, Y. , Benner, J. , Eberle, D. , Gehlen, K. , Hücherig, S. et al. (2009) Evolution of rosmarinic acid biosynthesis. Phytochemistry, 70, 1663–1679. Available from: 10.1016/j.phytochem.2009.05.010 19560175

[tpj70837-bib-0041] Petersen, M. & Alfermann, A.W. (1988) Two new enzymes of rosmarinic acid biosynthesis from cell cultures of *Coleus blumei*: hydroxyphenylpyruvate reductase and rosmarinic acid synthase. Zeitschrift für Naturforschung, 43, 501–504. Available from: 10.1515/znc-1988-7-804

[tpj70837-bib-0042] Petersen, M. , Häusler, E. , Karwatzki, B. & Meinhard, J. (1993) Proposed biosynthetic pathway for rosmarinic acid in cell cultures of *Coleus blumei* Benth. Planta, 189, 10–14. Available from: 10.1007/BF00201337

[tpj70837-bib-0043] Renault, H. , Alber, A. , Horst, N.A. , Basilio Lopes, A. , Fich, E.A. , Kriegshauser, L. et al. (2017) A phenol‐enriched cuticle is ancestral to lignin evolution in land plants. Nature Communications, 8, 14713. Available from: 10.1038/ncomms14713 PMC5344971 28270693

[tpj70837-bib-0044] Sander, M. & Petersen, M. (2011) Distinct substrate specificities and unusual substrate flexibilities of two hydroxycinnamoyltransferases, rosmarinic acid synthase and hydroxycinnamoyl‐CoA:shikimate hydroxycinnamoyltransferase, from *Coleus blumei* Benth. Planta, 233, 1157–1171. Available from: 10.1007/s00425-011-1367-2 21318289

[tpj70837-bib-0045] Sievers, F. , Wilm, A. , Dineen, D. , Gibson, T.J. , Karplus, K. , Li, W. et al. (2011) Fast, scalable generation of high‐quality protein multiple sequence alignments using Clustal Omega. Molecular Systems Biology, 7, 539. Available from: 10.1038/msb.2011.75 21988835 PMC3261699

[tpj70837-bib-0046] St. Pierre, B. & de Luca, V. (2000) Evolution of acyltransferase genes: origin and diversification of the BAHD superfamily of acyltransferases involved in secondary metabolism. Recent Advances in Phytochemistry, 34, 285–315. Available from: 10.1016/S0079-9920(00)80010-6

[tpj70837-bib-0047] Szabo, E. , Petersen, M. & Alfermann, A.W. (1993) Occurrence of rosmarinic acid in *Blechnum* species. Planta Medica, 59, A651.

[tpj70837-bib-0048] Takeda, R. , Hasegawa, J. & Shinozaki, M. (1990) The first isolation of lignans, megacerotonic acid and anthocerotonic acid, from non‐vascular plants, Anthocerotae (hornworts). Tetrahedron Letters, 31, 4159–4162. Available from: 10.1016/S0040-4039(00)97569-5

[tpj70837-bib-0049] Ube, N. , Yabuta, Y. , Tohnooka, T. , Ueno, K. , Taketa, S. & Ishihara, A. (2019) Biosynthesis of phenylamide phytoalexins in pathogen‐infected barley. International Journal of Molecular Sciences, 20, 5541. Available from: 10.3390/ijms20225541 31698855 PMC6888128

[tpj70837-bib-0050] Ufland, M. & Petersen, M. (2026) Phenolic compounds in species of the Blechnaceae. Plant Biology, 28, 282–291. Available from: 10.1111/plb.70116 41016049 PMC12710811

[tpj70837-bib-0051] Wang, C.Z. , Davin, L.B. & Lewis, N.G. (2001) Stereoselective phenolic coupling in *Blechnum spicant*: formation of 8‐2′ linked (‐)‐*cis*‐blechnic, (‐)‐*trans*‐blechnic and (‐)‐brainic acids. Chemical Communications, 113–114, 113–114. Available from: 10.1039/B008174O

[tpj70837-bib-0052] Waswa, E.N. , Muema, F.W. , Odago, W.O. , Mutinda, E.S. , Nanjala, C. , Mkala, E.M. et al. (2022) Traditional uses, phytochemistry, and pharmacological properties of the genus *Blechnum*—a narrative review. Pharmaceuticals, 15, 905. Available from: 10.3390/ph15070905 35890203 PMC9323518

[tpj70837-bib-0053] Weitzel, C. & Petersen, M. (2011) Cloning and characterisation of rosmarinic acid synthase from *Melissa officinalis* L. Phytochemistry, 72, 572–578. Available from: 10.1016/j.phytochem.2011.01.039 21354582

[tpj70837-bib-0054] Zhu, L. , Li, Y. , Yang, J. , Zuo, L. & Zhang, D. (2008) Studies on chemical constituents of *Sarcandra glabra* . Zhongguo Zhong Yao Za Zhi, 33, 155–157. 18619347

