# Supplementary material for: Identification and characterization of BAHD hydroxycinnamoyltransferases in the fern Neoblechnum brasiliense
Source: Plant J. 2026 Mar 29;126(1):e70837. doi: 10.1111/tpj.70837 (PMC13033379; doi:10.1111/tpj.70837)
Supplement: Supplementary file 1 — Figure S1. Nomenclature for shikimic and quinic acid esters. Figure S2. Western blot analysis. Figure S3. pH and temperature optima of NbHCT1, NbHCT3, and NbHCT8. Figure S4. Michaelis–Menten graphs for NbHCT1. Figure S5. Amino acid sequence identity and similarity of NbHCT1‐9. Figure S6. Michaelis–Menten graphs for NbHCT3. Figure S7. Michaelis–Menten graphs for NbHCT8. Figure S8. Extracted ion chromatograms (EIC) of substrate search assays. Figure S9. MS/MS fragmentation of 3/4/5‐O‐shikimic/quinic acid esters. Figure S10. Phylogenetic analysis of NbHCT3 orthologs. Figure S11. Phylogenetic analysis of NbHCT5 orthologs. Table S1. BLASTP search in the transcriptome of Struthiopteris spicant. Table S2. Screened putative NbHCT substrates and method of preparation and detection. Table S3. Investigated NBHCTs and their transformed acyl donors and acyl acceptors. Table S4. Summary of identified products formed in NbHCT1 assays. Table S5. Summary of identified products formed in NbHCT2 assays. Table S6. Summary of identified products formed in NbHCT3 assays. Table S7. Summary of identified products formed in NbHCT8 assays. Table S8. Summary of identified products formed in NbHCT5 assays. Table S9. Summary of identified products formed in NbHCT4 assays. Table S10. Summary of identified products formed in NbHCT6 assays. Table S11. Summary of identified products formed in NbHCT7 assays. Table S12. Summary of identified products formed in NbHCT9 assays. Table S13. Sequences used in the phylogenetic tree. Table S14. PCR conditions and primers for amplification of NbHCT1‐9. Table S15. Conditions, composition, and detection parameters for the determination of pH and temperature optima. Table S16. Conditions, composition, and detection parameters for the determination of kinetic parameters. [file TPJ-126-0-s001.pdf]

Supporting Information for

Identification and characterization of BAHD hydroxycinnamoyltransferases in the fern *Neoblechnum brasiliense*

Maximilian Ufland and Maike Petersen

Supporting Figures

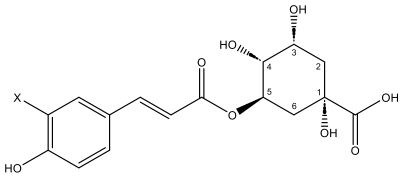

X = H *p*-coumaroyl-5-*O*-quinic acid
X= OH caffeoyl-5-*O*-quinic acid (chlorogenic acid)

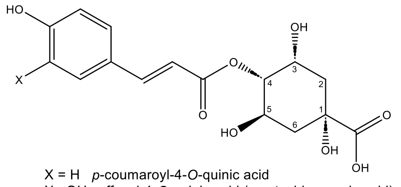

X = H *p*-coumaroyl-4-*O*-quinic acid
X= OH caffeoyl-4-*O*-quinic acid (cryptochlorogenic acid)

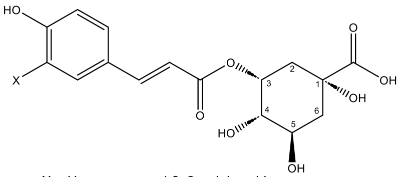

X = H *p*-coumaroyl-3-*O*-quinic acid
X= OH caffeoyl-3-*O*-quinic acid (neochlorogenic acid)

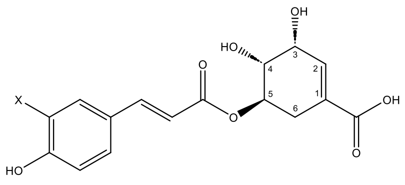

X = H *p*-coumaroyl-5-*O*-shikimic acid
X= OH caffeoyl-5-*O*-shikimic acid

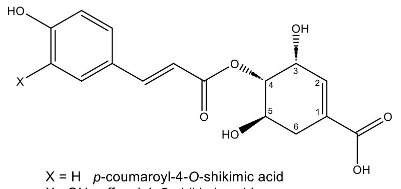

X = H *p*-coumaroyl-4-*O*-shikimic acid
X= OH caffeoyl-4-*O*-shikimic acid

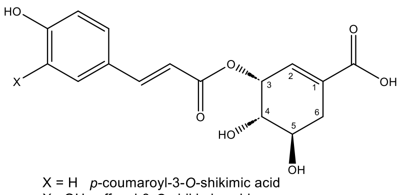

X = H *p*-coumaroyl-3-*O*-shikimic acid
X= OH caffeoyl-3-*O*-shikimic acid

Figure S1 Nomenclature for shikimic and quinic acid esters used in this paper following Abrankó and Clifford (2017).

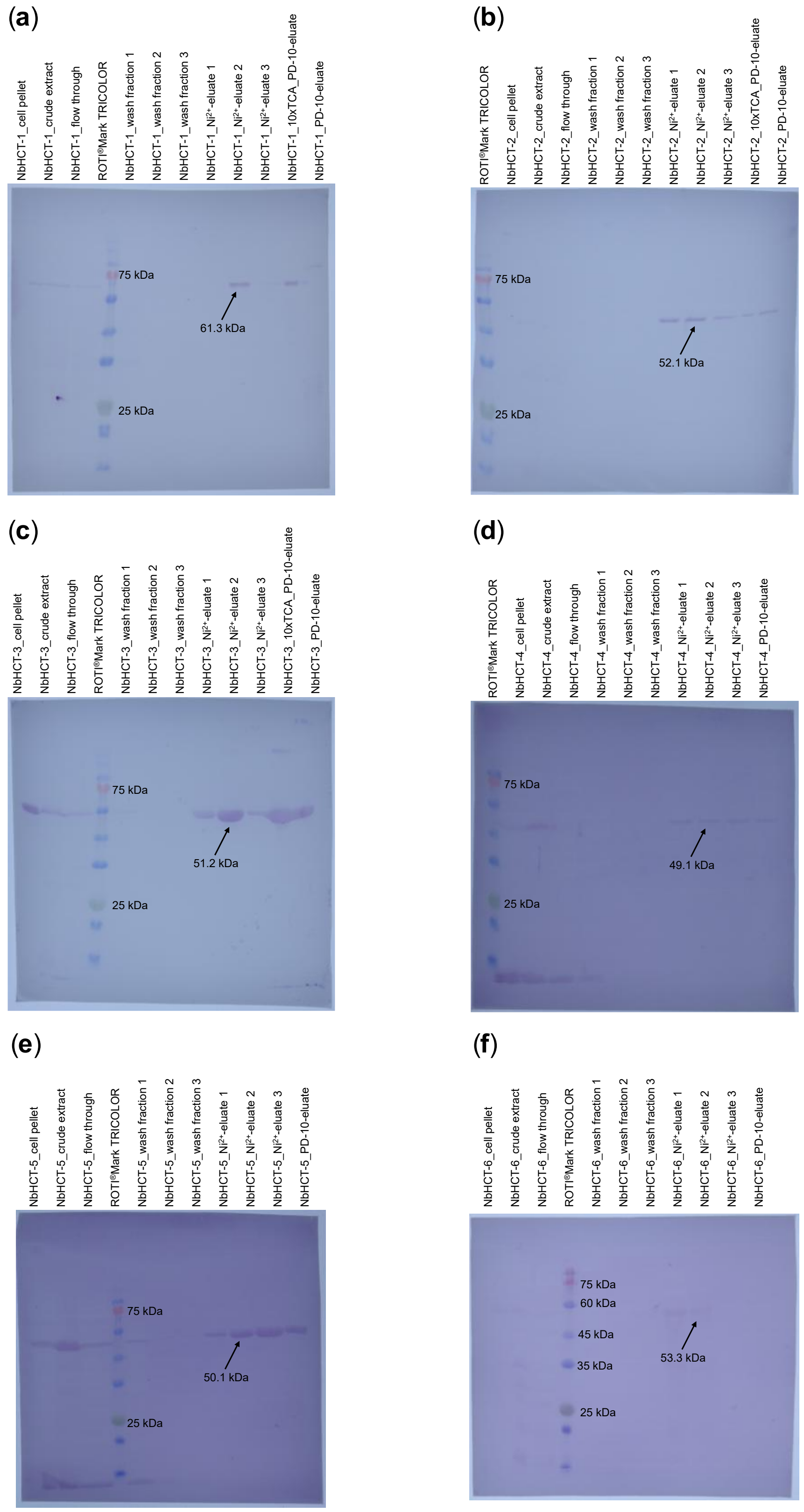
(a)
NbHCT-1_cell pellet
NbHCT-1_crude extract
NbHCT-1_flow through
ROTI®Mark TRICOLOR
NbHCT-1_wash fraction 1
NbHCT-1_wash fraction 2
NbHCT-1_wash fraction 3
NbHCT-1_Ni²⁺-eluate 1
NbHCT-1_Ni²⁺-eluate 2
NbHCT-1_Ni²⁺-eluate 3
NbHCT-1_10xTCA_PD-10-eluate
NbHCT-1_PD-10-eluate
75 kDa
61.3 kDa
25 kDa
(b)
ROTI®Mark TRICOLOR
NbHCT-2_cell pellet
NbHCT-2_crude extract
NbHCT-2_flow through
NbHCT-2_wash fraction 1
NbHCT-2_wash fraction 2
NbHCT-2_wash fraction 3
NbHCT-2_Ni²⁺-eluate 1
NbHCT-2_Ni²⁺-eluate 2
NbHCT-2_Ni²⁺-eluate 3
NbHCT-2_10xTCA_PD-10-eluate
NbHCT-2_PD-10-eluate
75 kDa
52.1 kDa
25 kDa
(c)
NbHCT-3_cell pellet
NbHCT-3_crude extract
NbHCT-3_flow through
ROTI®Mark TRICOLOR
NbHCT-3_wash fraction 1
NbHCT-3_wash fraction 2
NbHCT-3_wash fraction 3
NbHCT-3_Ni²⁺-eluate 1
NbHCT-3_Ni²⁺-eluate 2
NbHCT-3_Ni²⁺-eluate 3
NbHCT-3_10xTCA_PD-10-eluate
NbHCT-3_PD-10-eluate
75 kDa
51.2 kDa
25 kDa
(d)
ROTI®Mark TRICOLOR
NbHCT-4_cell pellet
NbHCT-4_crude extract
NbHCT-4_flow through
NbHCT-4_wash fraction 1
NbHCT-4_wash fraction 2
NbHCT-4_wash fraction 3
NbHCT-4_Ni²⁺-eluate 1
NbHCT-4_Ni²⁺-eluate 2
NbHCT-4_Ni²⁺-eluate 3
NbHCT-4_PD-10-eluate
75 kDa
49.1 kDa
25 kDa
(e)
NbHCT-5_cell pellet
NbHCT-5_crude extract
NbHCT-5_flow through
ROTI®Mark TRICOLOR
NbHCT-5_wash fraction 1
NbHCT-5_wash fraction 2
NbHCT-5_wash fraction 3
NbHCT-5_Ni²⁺-eluate 1
NbHCT-5_Ni²⁺-eluate 2
NbHCT-5_Ni²⁺-eluate 3
NbHCT-5_PD-10-eluate
75 kDa
50.1 kDa
25 kDa
(f)
NbHCT-6_cell pellet
NbHCT-6_crude extract
NbHCT-6_flow through
ROTI®Mark TRICOLOR
NbHCT-6_wash fraction 1
NbHCT-6_wash fraction 2
NbHCT-6_wash fraction 3
NbHCT-6_Ni²⁺-eluate 1
NbHCT-6_Ni²⁺-eluate 2
NbHCT-6_Ni²⁺-eluate 3
NbHCT-6_PD-10-eluate
75 kDa
60 kDa
45 kDa
53.3 kDa
35 kDa
25 kDa

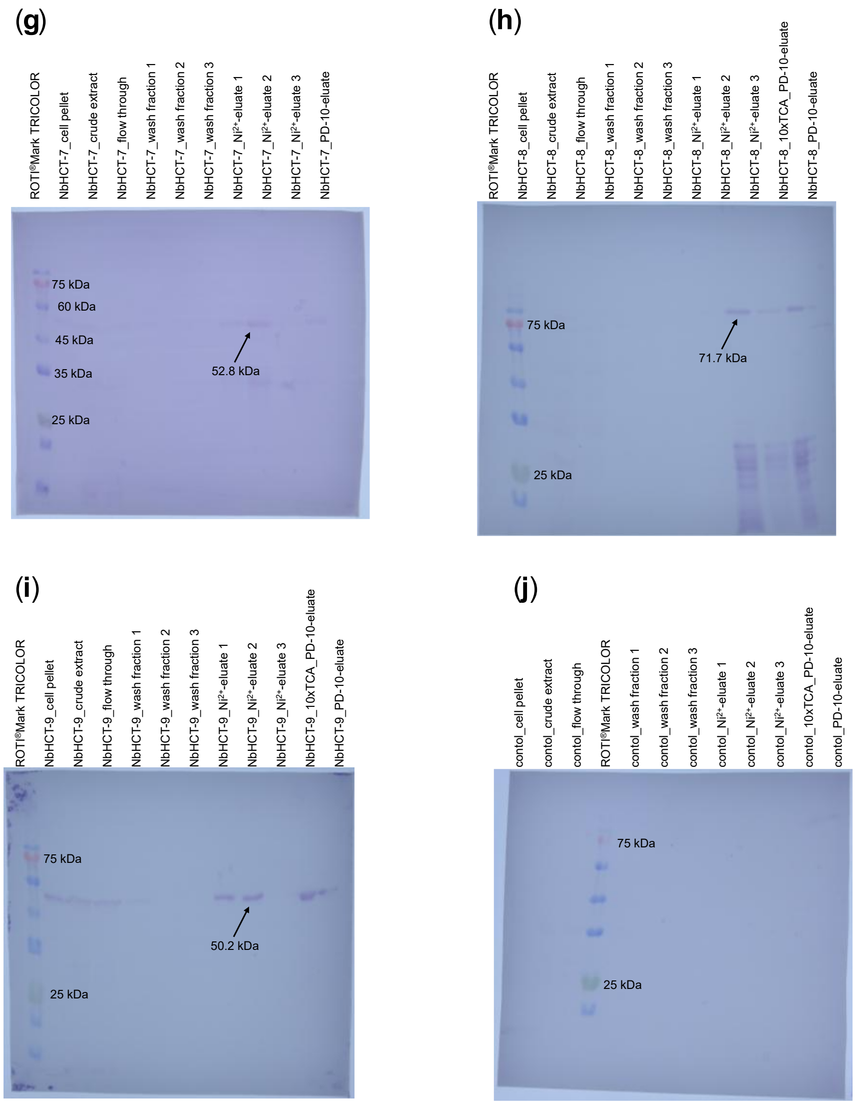

Figure S2 Western Blot analysis of NbHCT1 (**a**), NbHCT2 (**b**), NbHCT3 (**c**), NbHCT4 (**d**), NbHCT5 (**e**), NbHCT6 (**f**), NbHCT7 (**g**), NbHCT8 (**h**), NbHCT9 (**i**) and empty vector control (**j**). Arrows point on the visualized BAHD acyltransferase with the calculated mass of the protein including the 6xHis-Tag.

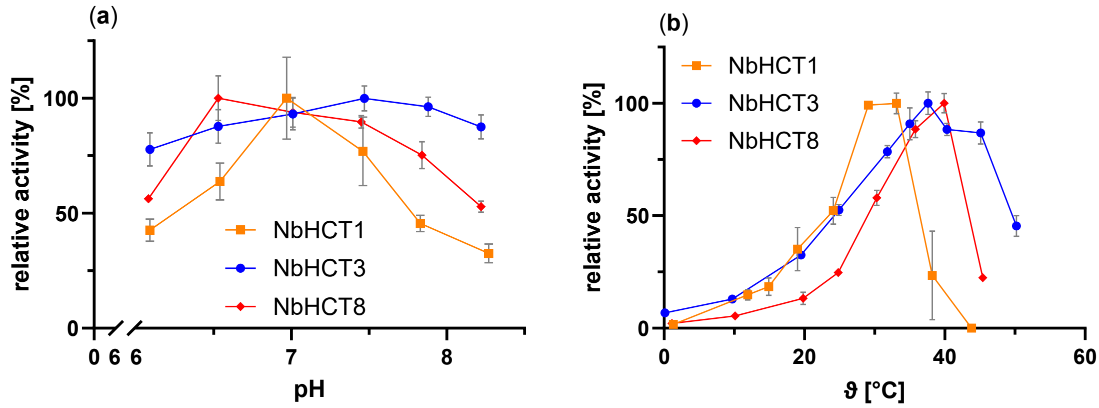

Figure S3 pH and temperature optima of NbHCT1, NbHCT3 and NbHCT8. (**a**) pH optimum of NbHCT1 for the formation of caffeoyl-5-*O*-shikimic acid (100% ≙ 0.296 mkat/kg), NbHCT3 for the formation of *p*-coumaroyl-5-*O*-quinic acid (100% ≙ 3.68 mkat/kg) and NbHCT8 for the formation of *p*-coumaroyl-5-*O*-shikimic acid (100% ≙ 0.840 mkat/kg). (**b**) temperature optimum of NbHCT1 for the formation of caffeoyl-5-*O*-shikimic acid (100% ≙ 0.253 mkat/kg), NbHCT3 for the formation of *p*-coumaroyl-5-*O*-quinic acid (100% ≙ 13.4 mkat/kg) and NbHCT8 for the formation of *p*-coumaroyl-5-*O*-shikimic acid (100% ≙ 1.13 mkat/kg).

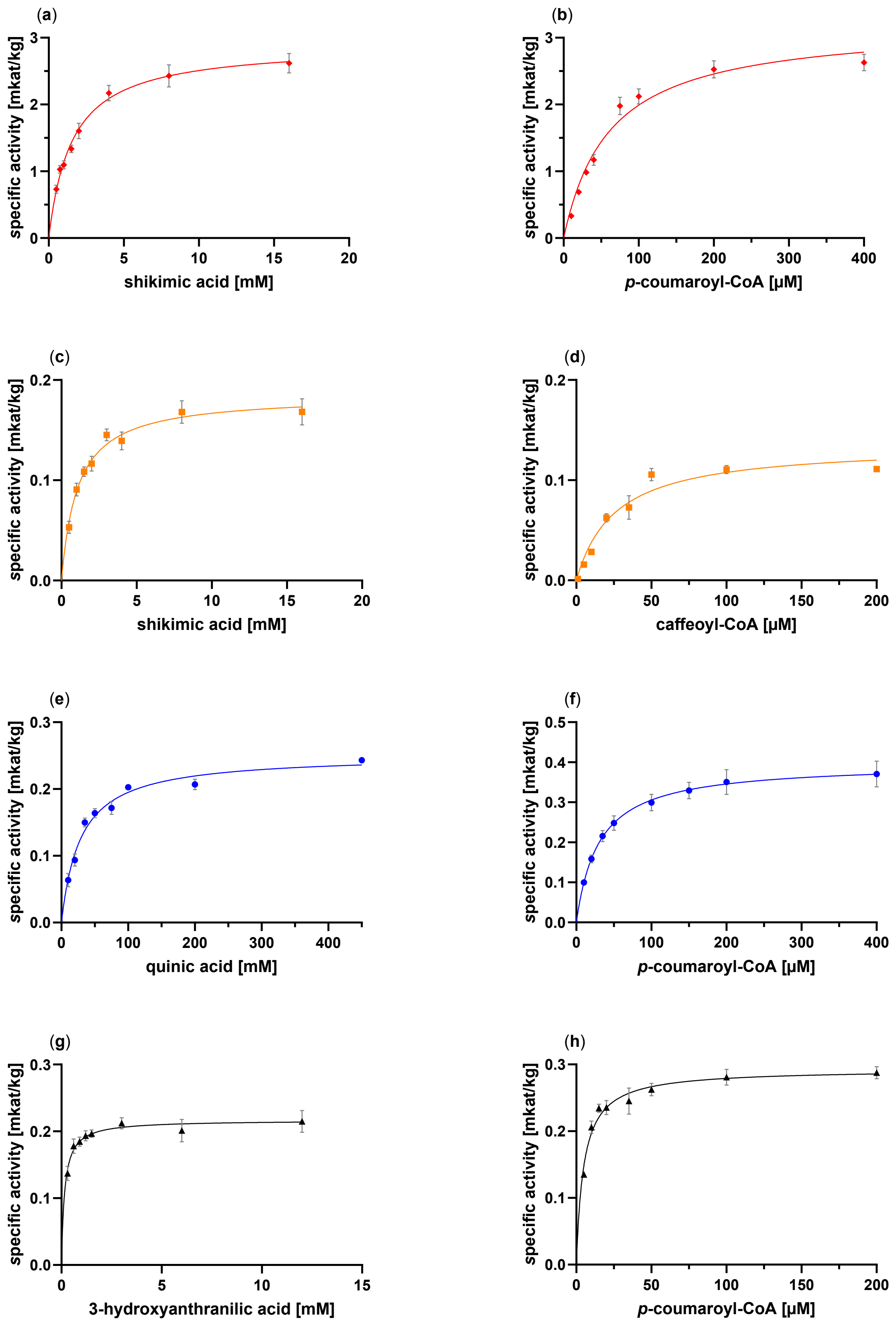

Figure S4 Michaelis-Menten graphs for NbHCT1 with variable concentrations of acceptors (left) and donors (right). Substrate saturation curves for (**a**) shikimic acid with *p*-coumaroyl-CoA; (**b**) *p*-coumaroyl-CoA with shikimic acid; (**c**) shikimic acid with caffeoyl-CoA; (**d**) caffeoyl-

CoA with shikimic acid; (**e**) quinic acid with *p*-coumaroyl-CoA; (**f**) *p*-coumaroyl-CoA with quinic acid; (**g**) 3-hydroxyanthranilic acid with *p*-coumaroyl-CoA; (**h**) *p*-coumaroyl-CoA with 3-hydroxyanthranilic acid (n = 9 ± SEM).

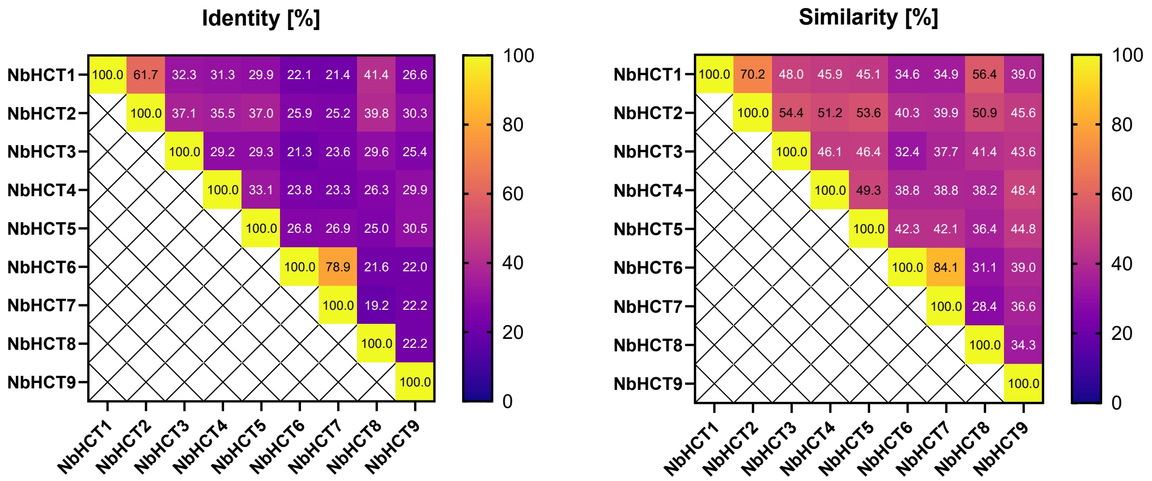

Figure S5 Amino acid sequence identity and similarity (EMBOSS Needle) of NbHCT1-9.

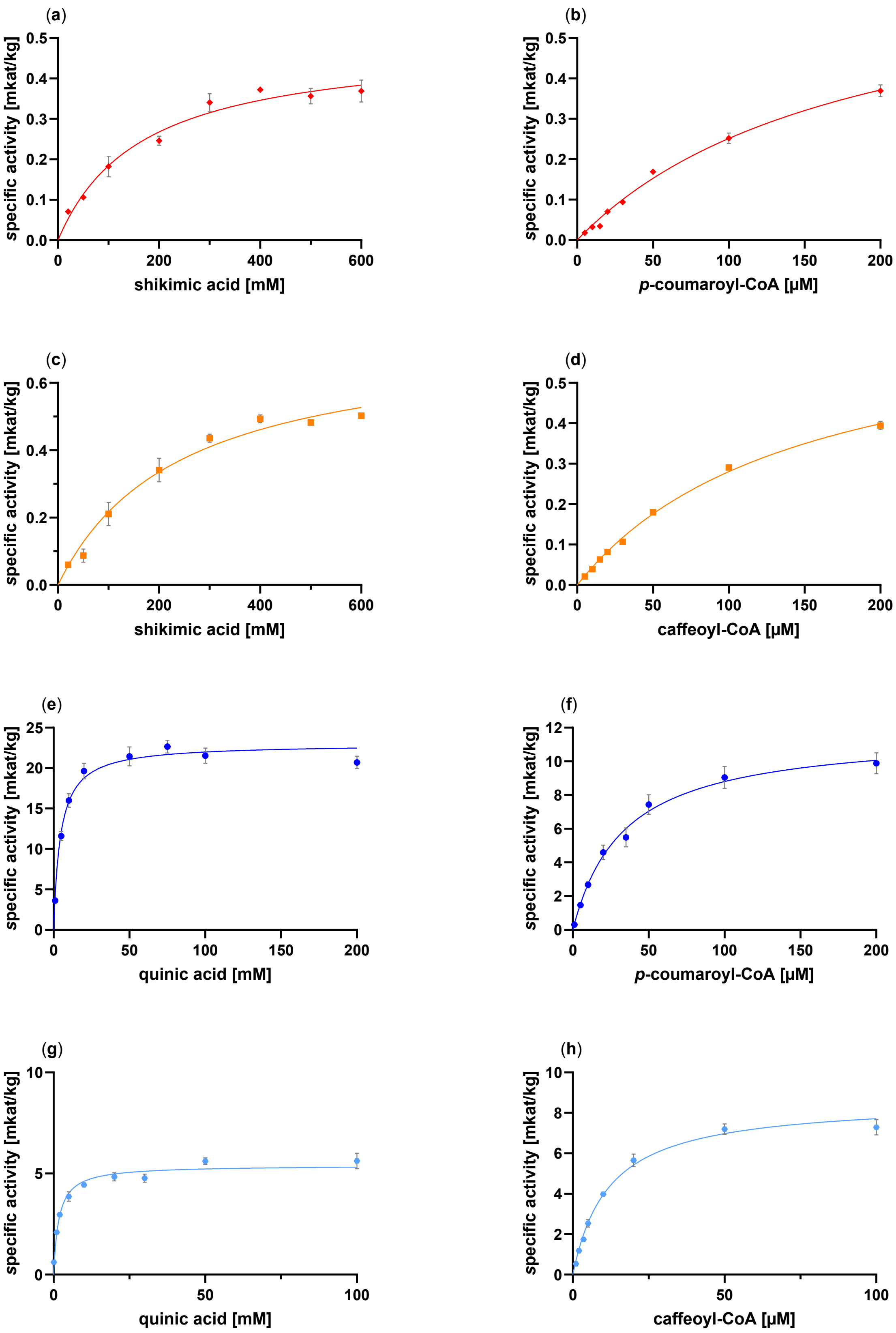

Figure S6 Michaelis-Menten graphs for NbHCT3 with variable concentrations of acceptors (left) and donors (right). Substrate saturation curves for (**a**) shikimic acid with *p*-coumaroyl-CoA; (**b**) *p*-coumaroyl-CoA with shikimic acid; (**c**) shikimic acid with caffeoyl-CoA; (**d**) caffeoyl-

CoA with shikimic acid; (**e**) quinic acid with *p*-coumaroyl-CoA; (**f**) *p*-coumaroyl-CoA with quinic acid; (**g**) quinic acid with caffeoyl-CoA; (**h**) caffeoyl-CoA with quinic acid. (**a**)-(**d**) $n = 3 \pm$ SEM, evaluated with plots according to Cornish-Bowden and Eisenthal (1978); (**e**)-(**h**) $n = 9 \pm$ SEM.

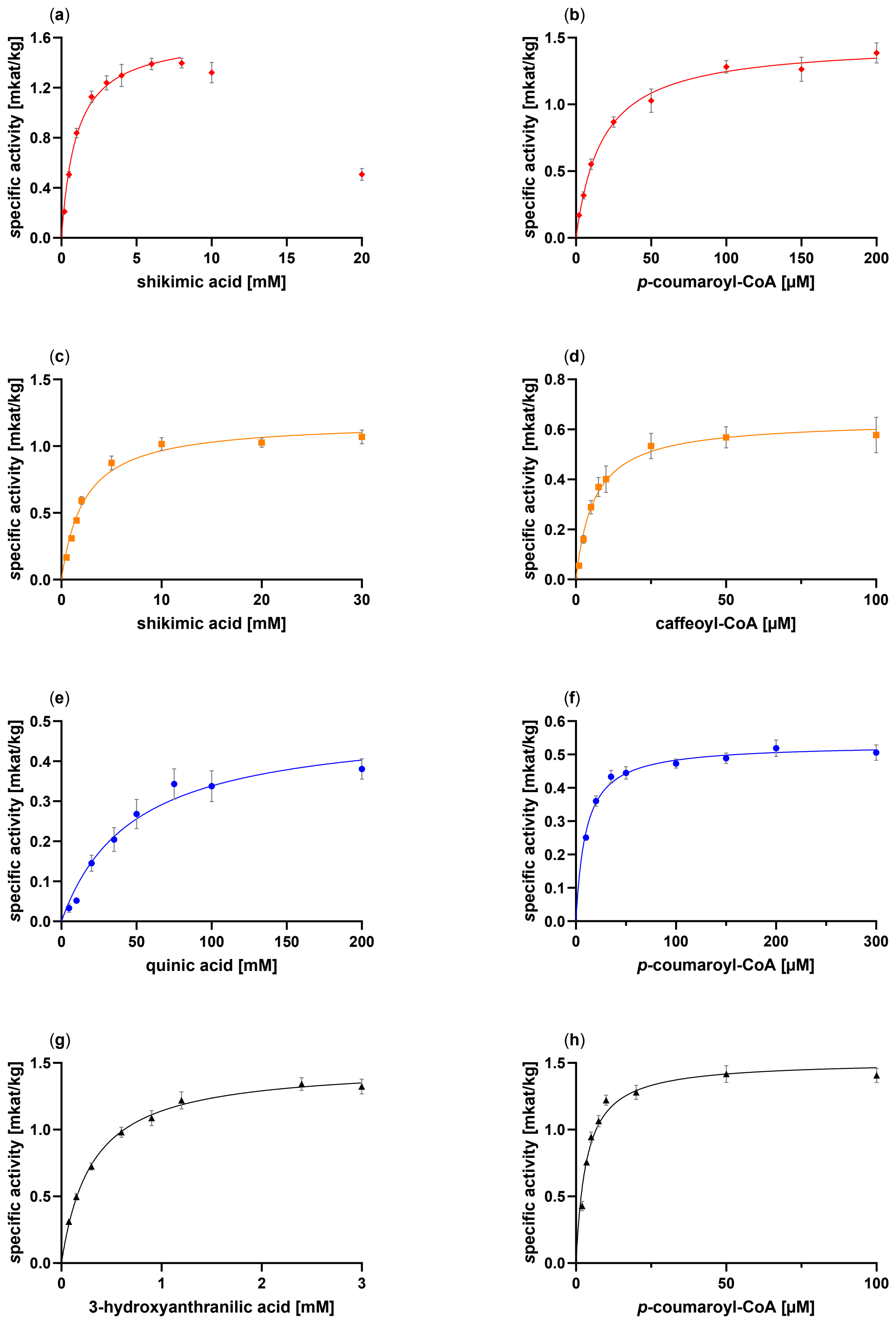

Figure S7 Michaelis-Menten graphs for NbHCT8 with variable concentrations of acceptors (left) and donors (right). Substrate saturation curves for (**a**) shikimic acid with *p*-coumaroyl-CoA, substrate inhibition by shikimic acid is observed at higher concentrations; (**b**) *p*-

coumaroyl-CoA with shikimic acid; (**c**) shikimic acid with caffeoyl-CoA; (**d**) caffeoyl-CoA with shikimic acid; (**e**) quinic acid with *p*-coumaroyl-CoA; (**f**) *p*-coumaroyl-CoA with quinic acid; (**g**) 3-hydroxyanthranilic acid with *p*-coumaroyl-CoA; (**h**) *p*-coumaroyl-CoA with 3-hydroxyanthranilic acid (n = 9 ± SEM).

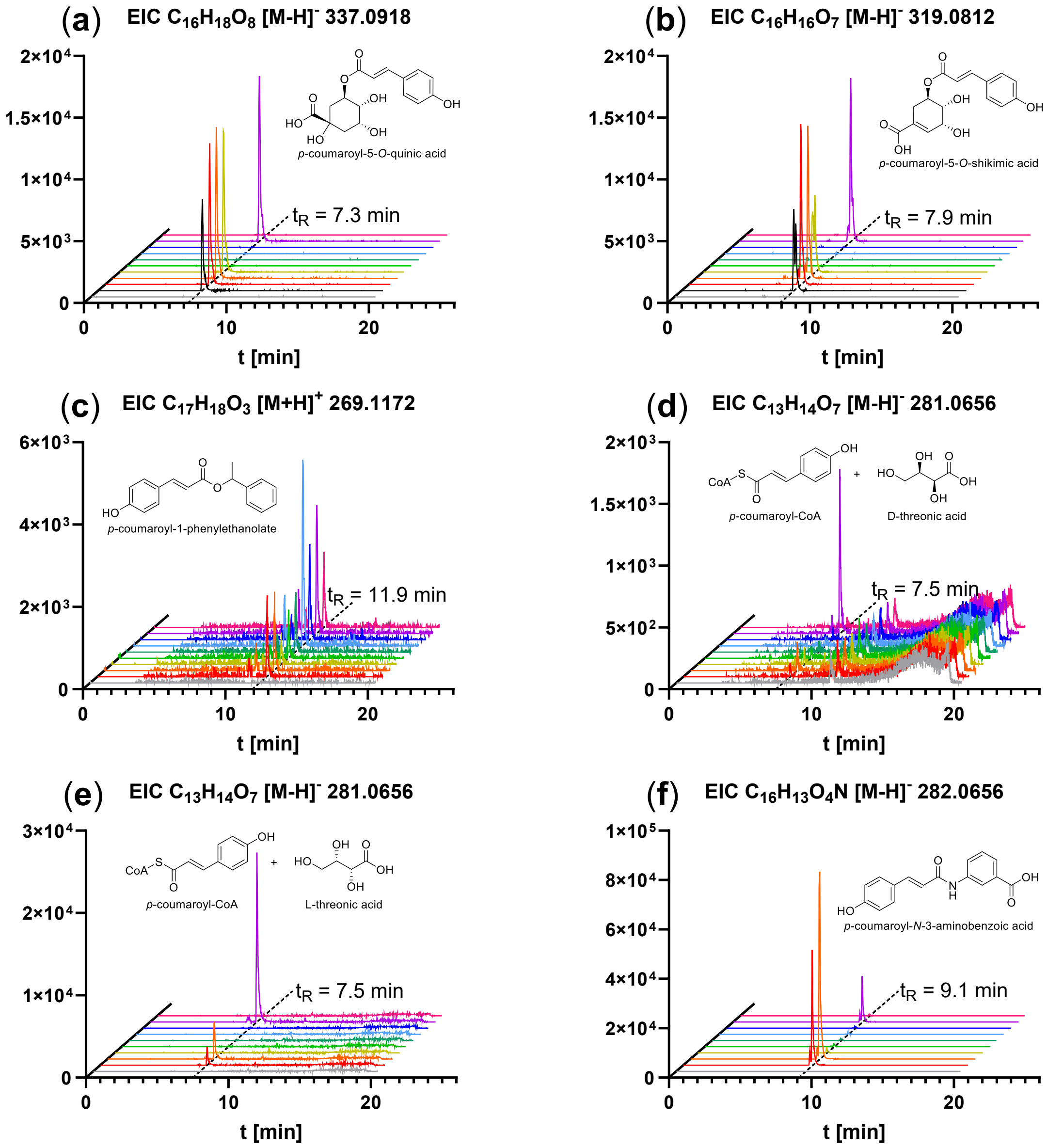

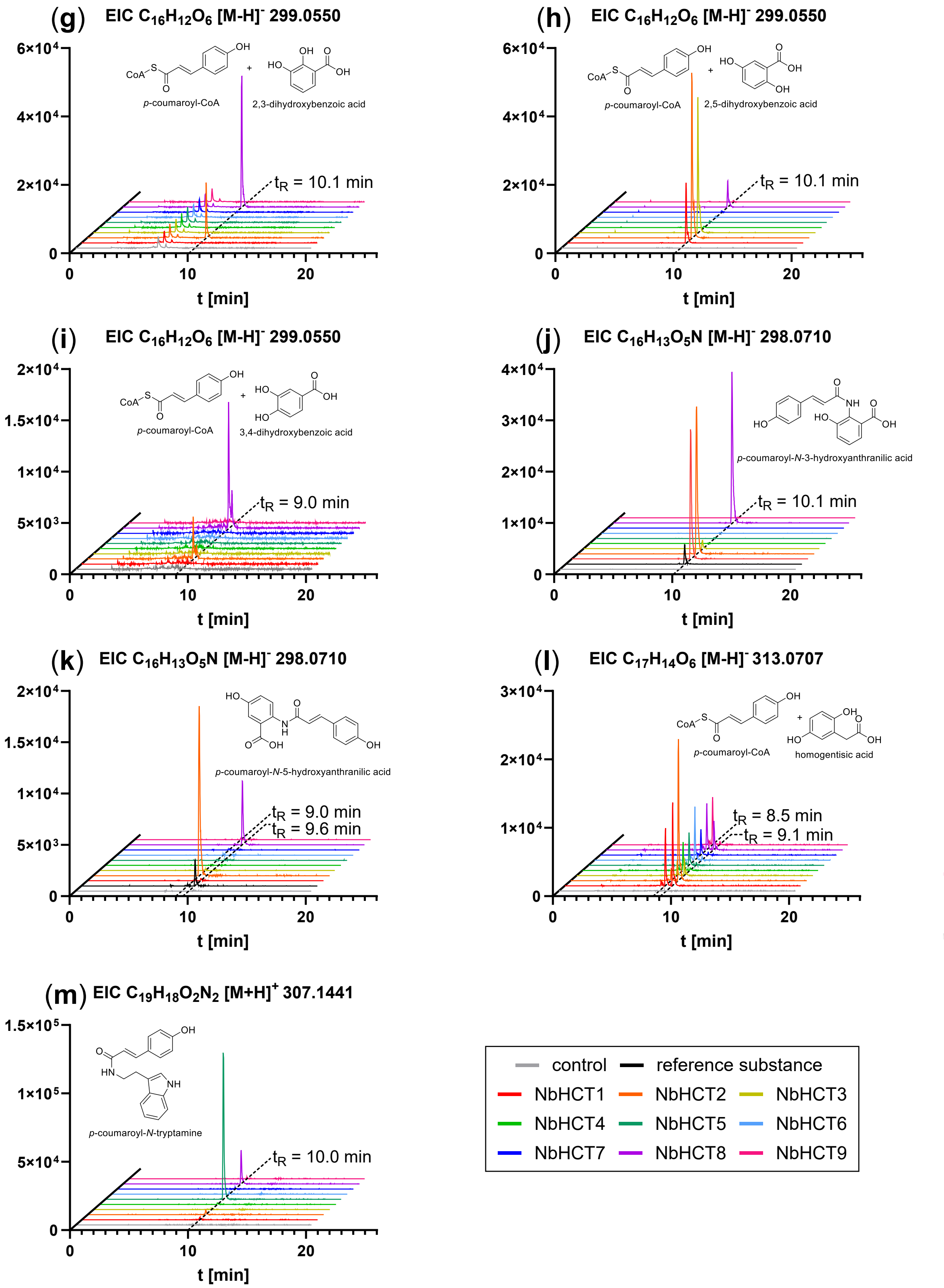

Figure S8 Extracted ion chromatograms (EIC) of substrate search assays with *p*-coumaroyl-CoA as acyl donor: grey (control), black (reference substance), red (NbHCT1), orange (NbHCT2), yellow (NbHCT3), light green (NbHCT4), dark green (NbHCT5), light blue (NbHCT6), dark blue (NbHCT7), purple (NbHCT8), pink (NbHCT9). Extracted ion

chromatograms (EIC) for the formation of (**a**) *p*-coumaroyl-5-*O*-quinic acid, (**b**) *p*-coumaroyl-5-*O*-shikimic acid, (**c**) *p*-coumaroyl-1-phenylethanol, (**d**) ester of *p*-coumaric and D-threonic acids, (**e**) ester of *p*-coumaric and L-threonic acids, (**f**) *p*-coumaroyl-*N*-3-aminobenzoic acid, (**g**) ester of *p*-coumaric and 2,3-dihydroxybenzoic acids, (**h**) ester of *p*-coumaric and 2,5-dihydroxybenzoic acids, (**i**) ester of *p*-coumaric and 3,4-dihydroxybenzoic acids, (**j**) *p*-coumaroyl-*N*-3-hydroxyanthranilic acid, (**k**) *p*-coumaroyl-*N*-5-hydroxyanthranilic acid, (**l**) ester of *p*-coumaric and homogentisic acids, (**m**) *p*-coumaroyl-*N*-tryptamine.

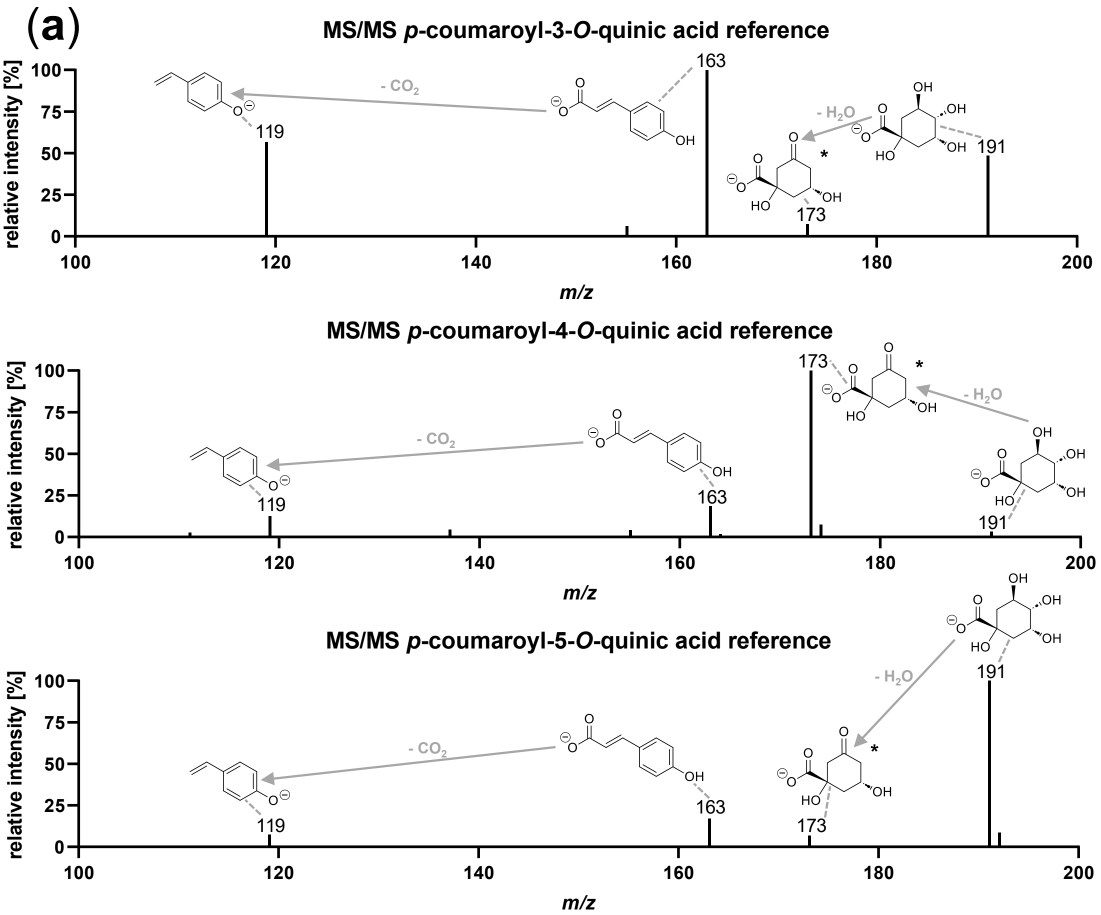

MS/MS caffeoyl-3-*O*-quinic acid reference

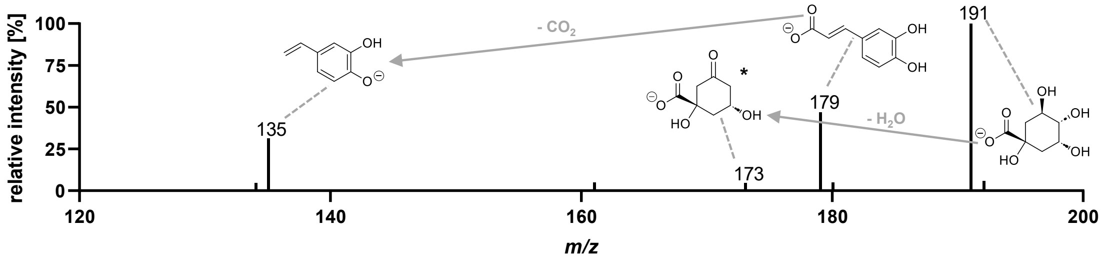

MS/MS caffeoyl-4-*O*-quinic acid reference

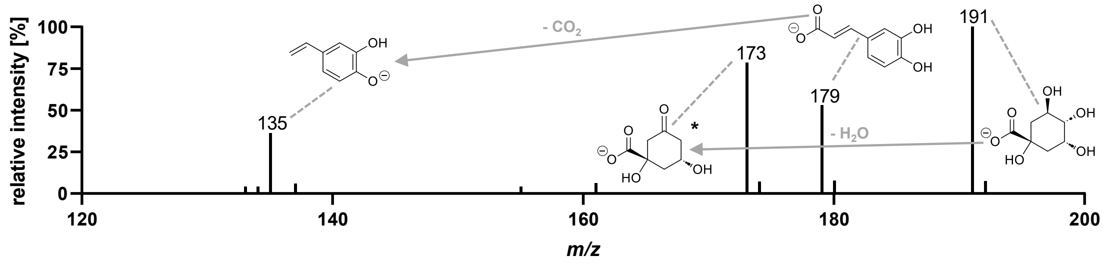

MS/MS caffeoyl-5-*O*-quinic acid reference

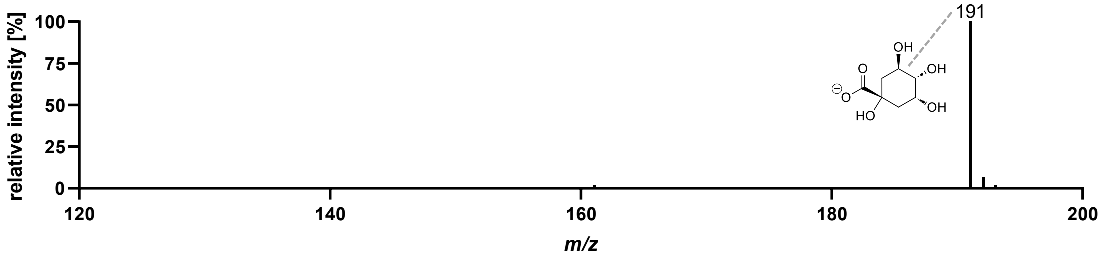

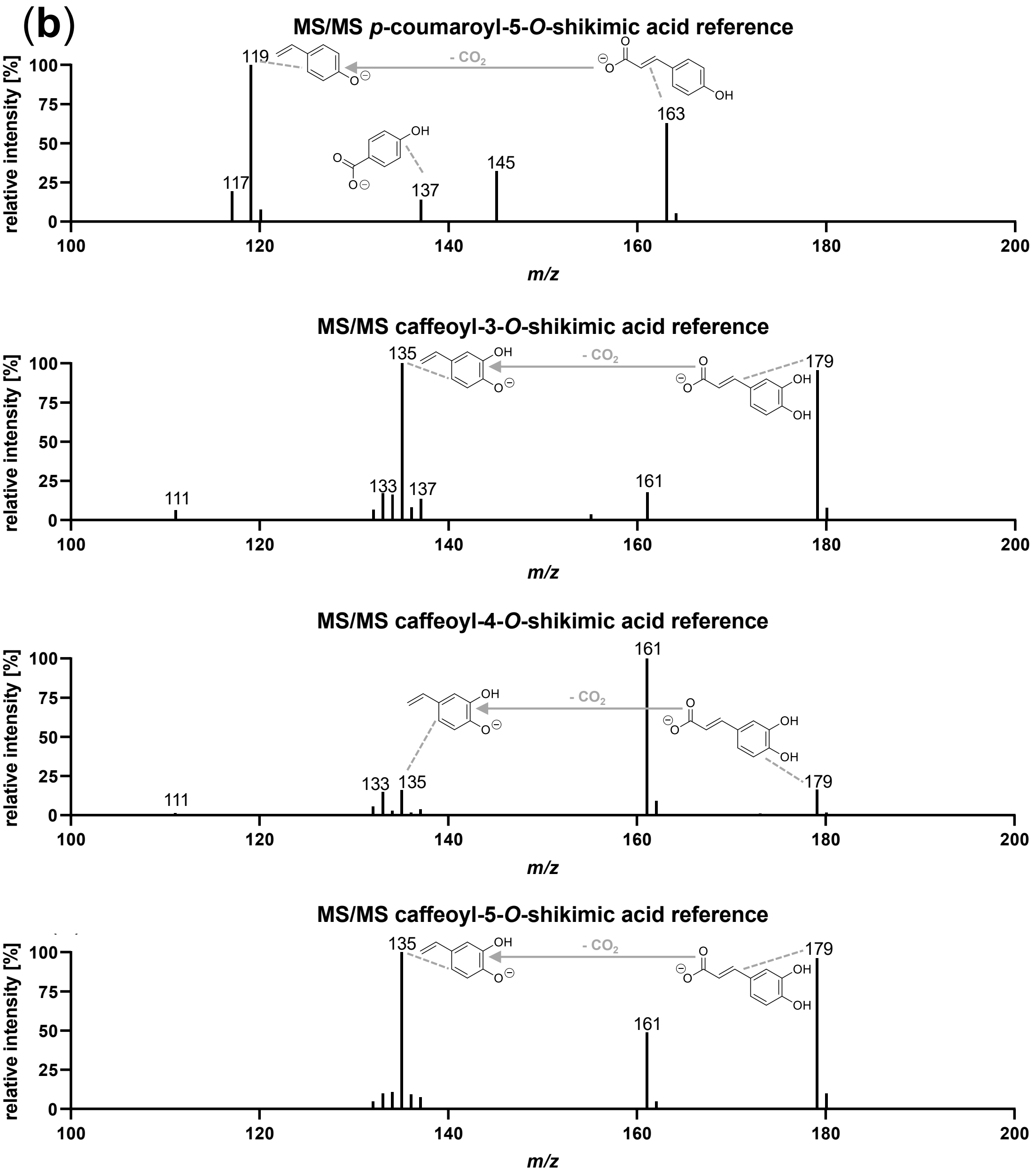
(b)
MS/MS p-coumaroyl-5-O-shikimic acid reference
relative intensity [%]
m/z
119
117
137
145
163
- CO2
OH
MS/MS caffeoyl-3-O-shikimic acid reference
relative intensity [%]
m/z
111
133
135
137
161
179
- CO2
OH
MS/MS caffeoyl-4-O-shikimic acid reference
relative intensity [%]
m/z
111
133
135
161
179
- CO2
OH
MS/MS caffeoyl-5-O-shikimic acid reference
relative intensity [%]
m/z
135
161
179
- CO2
OH

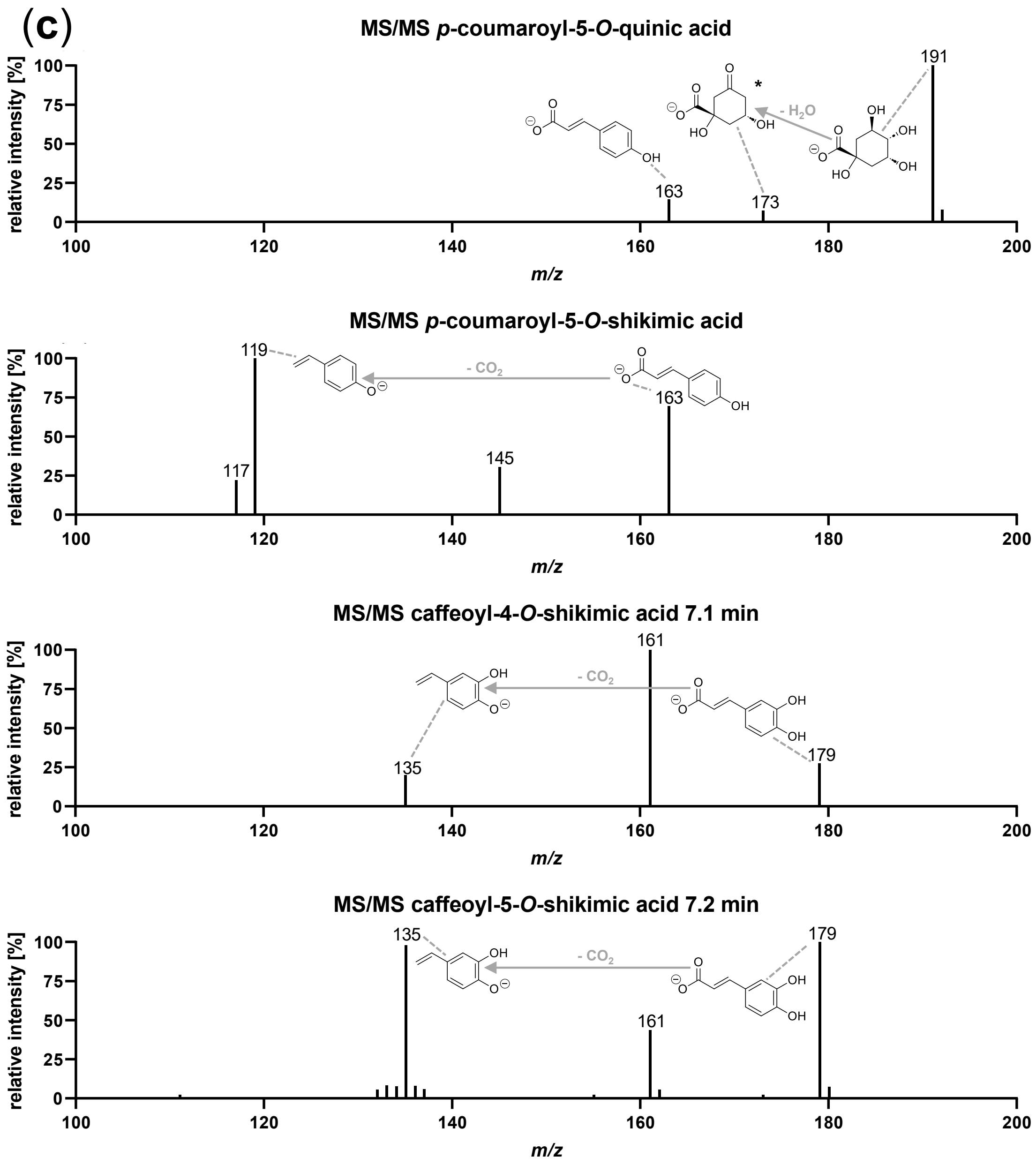
(c)
MS/MS p-coumaroyl-5-O-quinic acid
relative intensity [%]
m/z
191
163
173
- H2O
*
MS/MS p-coumaroyl-5-O-shikimic acid
119
117
145
163
- CO2
MS/MS caffeoyl-4-O-shikimic acid 7.1 min
161
135
179
- CO2
MS/MS caffeoyl-5-O-shikimic acid 7.2 min
135
161
179
- CO2

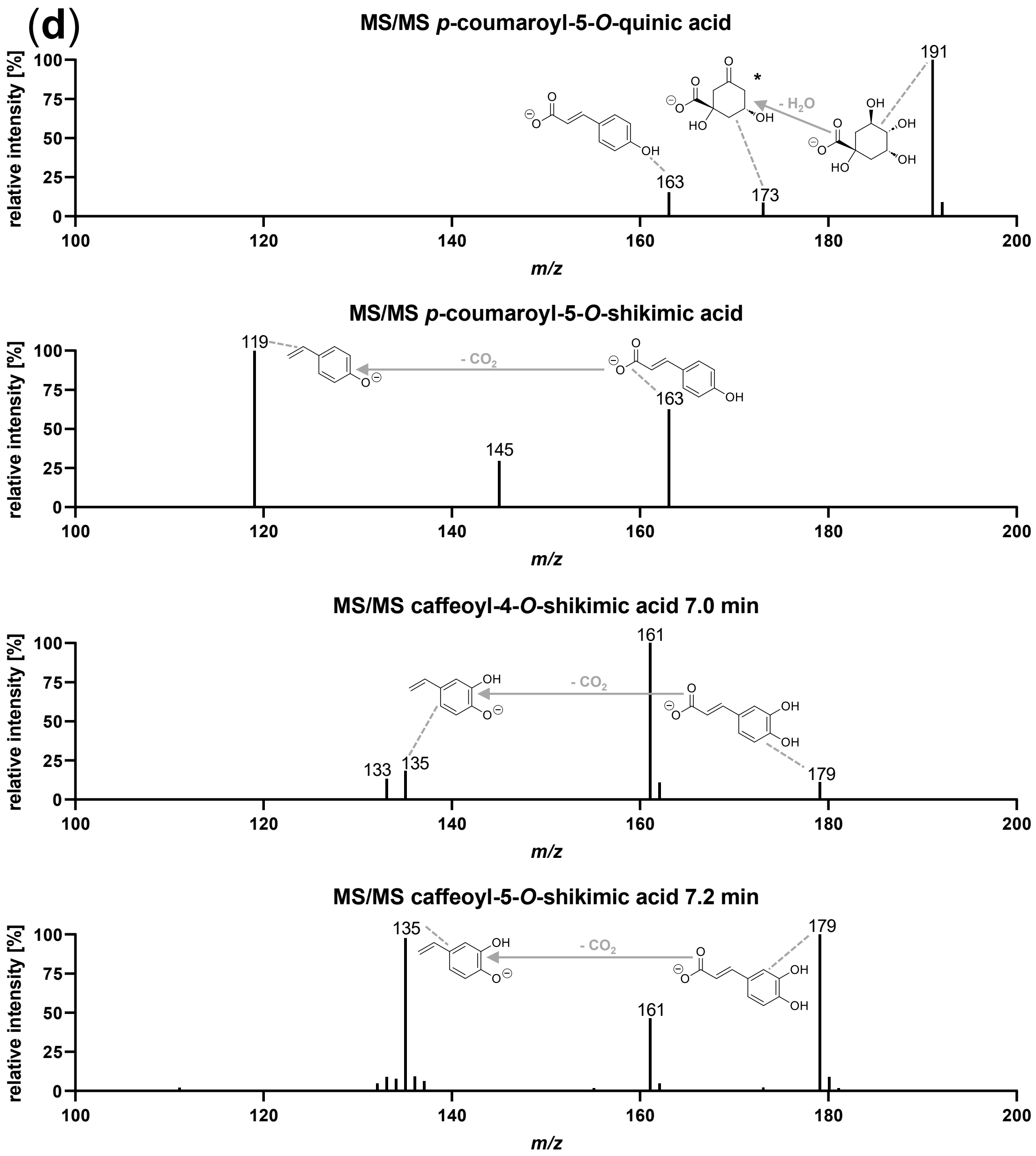

(d)
MS/MS p-coumaroyl-5-O-quinic acid
relative intensity [%]
m/z
191
163
173
- H2O
*
MS/MS p-coumaroyl-5-O-shikimic acid
119
145
163
- CO2
MS/MS caffeoyl-4-O-shikimic acid 7.0 min
161
133
135
179
- CO2
MS/MS caffeoyl-5-O-shikimic acid 7.2 min
135
161
179
- CO2

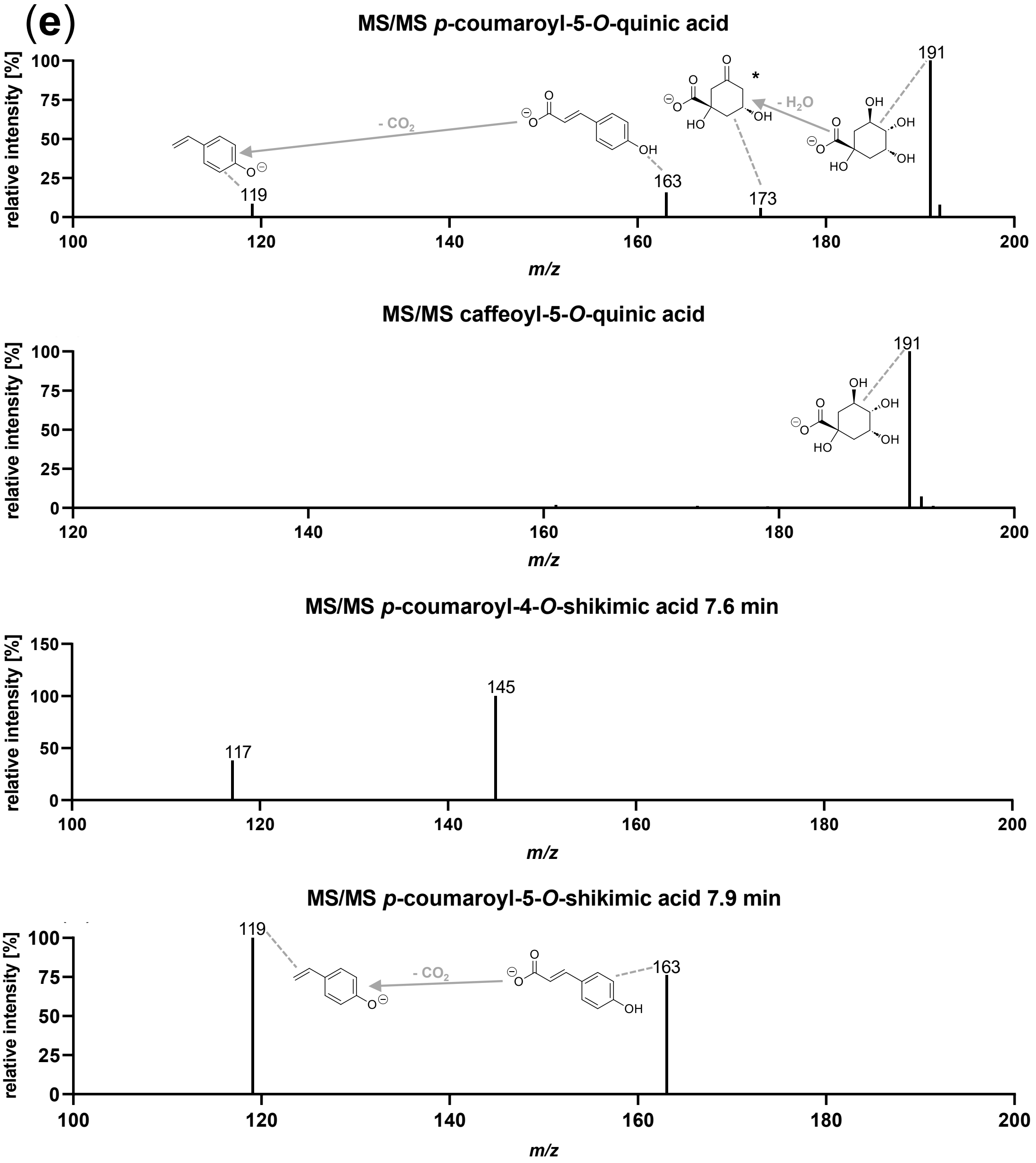

(e)
MS/MS p-coumaroyl-5-O-quinic acid
relative intensity [%]
- CO2
- H2O
119
163
173
191
m/z
MS/MS caffeoyl-5-O-quinic acid
relative intensity [%]
191
m/z
MS/MS p-coumaroyl-4-O-shikimic acid 7.6 min
relative intensity [%]
117
145
m/z
MS/MS p-coumaroyl-5-O-shikimic acid 7.9 min
relative intensity [%]
119
- CO2
163
m/z

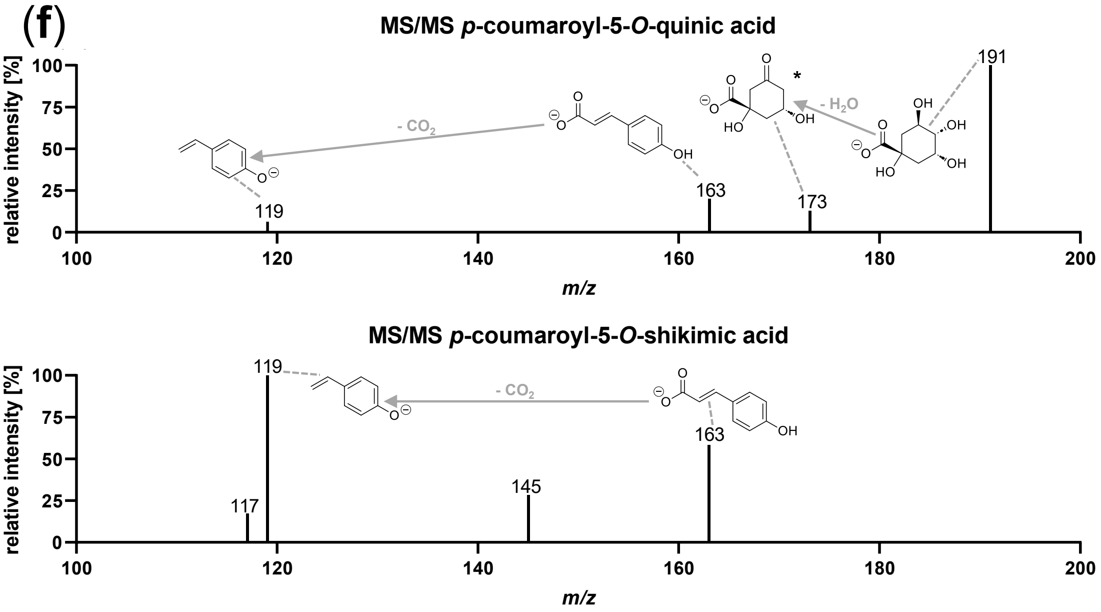

Figure S9 MS/MS fragmentation of 3/4/5-*O*-shikimic/quinic acid esters. The water loss (*) shown at fragment 173 is only one possibility. If no MS/MS event occurred, the retention time was used to determine the regio isomer (see Tables S4-S12). Note, that the 4/5-*O* derivatives elute with nearly identical retention times (4-*O* earlier than 5-*O*) and the 3-*O* derivative much earlier for quinic acid or much later for shikimic acid derivatives. (**a**) MS/MS fragmentation of reference compounds of *p*-coumaroyl/caffeoyl-3/4/5-*O*-quinic acid esters, (**b**) MS/MS fragmentation of reference compounds of *p*-coumaroyl-5-*O*-shikimic acid and caffeoyl-3/4/5-*O*-shikimic acid esters, (**c**) MS/MS fragmentation of quinic and shikimic acid esters formed by NbHCT1, (**d**) MS/MS fragmentation of quinic and shikimic acid esters formed by NbHCT2, (**e**) MS/MS fragmentation of quinic and shikimic acid esters formed by NbHCT3, (**f**) MS/MS fragmentation of quinic and shikimic acid esters formed by NbHCT8.

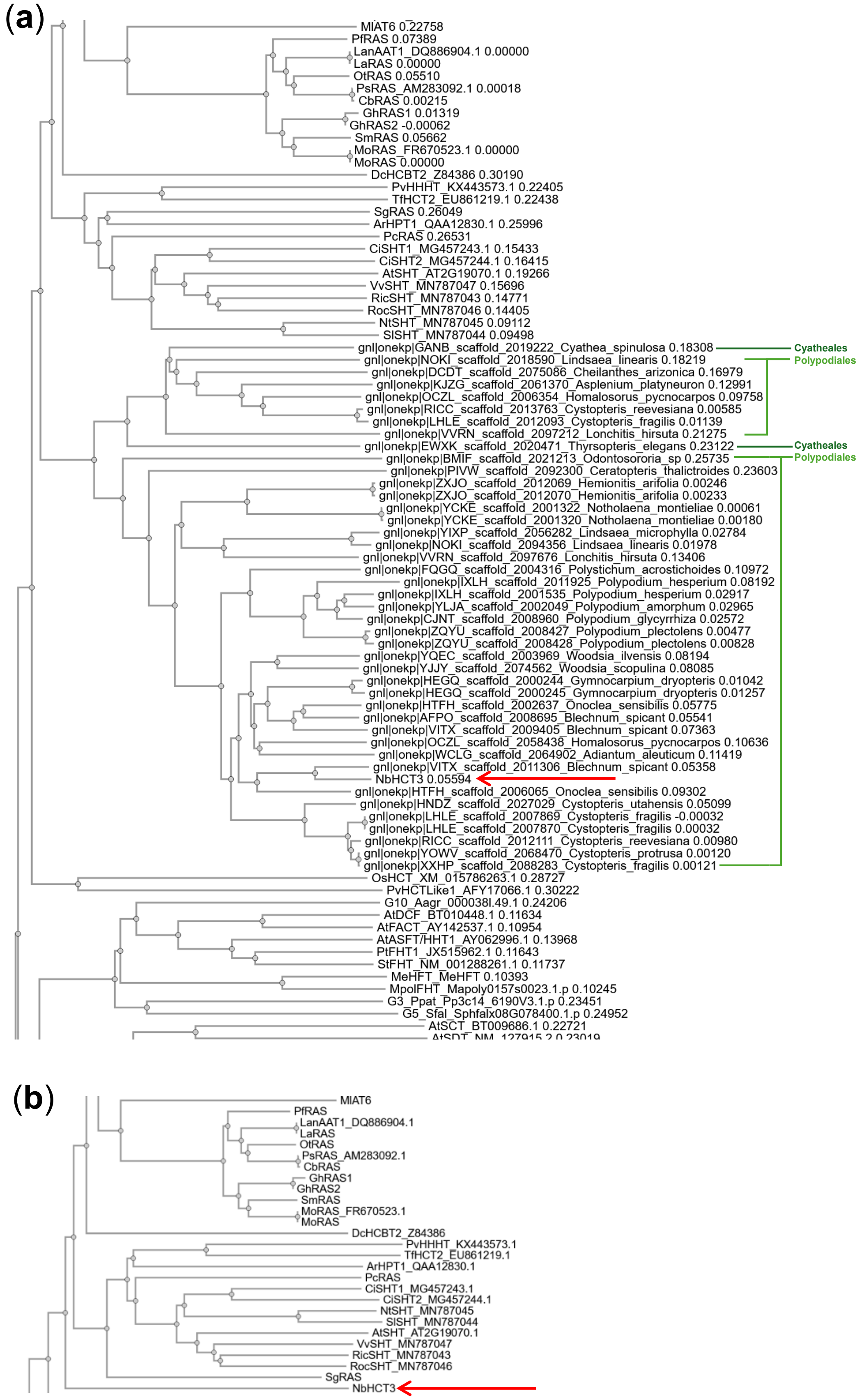

(a)
MlAT6 0.22758
PfRAS 0.07389
LanAAT1_DQ886904.1 0.00000
LaRAS 0.00000
OtRAS 0.05510
PsRAS_AM283092.1 0.00018
CbRAS 0.00215
GhRAS1 0.01319
GhRAS2 -0.00062
SmRAS 0.05662
MoRAS_FR670523.1 0.00000
MoRAS 0.00000
DcHCBT2_Z84386 0.30190
PvHHHT_KX443573.1 0.22405
TfHCT2_EU861219.1 0.22438
SgRAS 0.26049
ArHPT1_QAA12830.1 0.25996
PcRAS 0.26531
CiSHT1_MG457243.1 0.15433
CiSHT2_MG457244.1 0.16415
AtSHT_AT2G19070.1 0.19266
VvSHT_MN787047 0.15696
RicSHT_MN787043 0.14771
RocSHT_MN787046 0.14405
NtSHT_MN787045 0.09112
SlSHT_MN787044 0.09498
gnl|onekp|GANB_scaffold_2019222_Cyathea_spinulosa 0.18308
gnl|onekp|NOKI_scaffold_2018590_Lindsaea_linearis 0.18219
gnl|onekp|DCDT_scaffold_2075086_Cheilanthes_arizonica 0.16979
gnl|onekp|KJZG_scaffold_2061370_Asplenium_platyneuron 0.12991
gnl|onekp|OCZL_scaffold_2006354_Homalosorus_pycnocarpos 0.09758
gnl|onekp|RICC_scaffold_2013763_Cystopteris_reevesiana 0.00585
gnl|onekp|LHLE_scaffold_2012093_Cystopteris_fragilis 0.01139
gnl|onekp|VVRN_scaffold_2097212_Lonchitis_hirsuta 0.21275
gnl|onekp|EWXK_scaffold_2020471_Thyrsopteris_elegans 0.23122
gnl|onekp|BMIF_scaffold_2021213_Odontosororia_sp 0.25735
gnl|onekp|PIVW_scaffold_2092300_Ceratopteris_thalictroides 0.23603
gnl|onekp|ZXJO_scaffold_2012069_Hemionitis_arifolia 0.00246
gnl|onekp|ZXJO_scaffold_2012070_Hemionitis_arifolia 0.00233
gnl|onekp|YCKE_scaffold_2001322_Notholaena_montieliae 0.00061
gnl|onekp|YCKE_scaffold_2001320_Notholaena_montieliae 0.00180
gnl|onekp|YIXP_scaffold_2056282_Lindsaea_microphylla 0.02784
gnl|onekp|NOKI_scaffold_2094356_Lindsaea_linearis 0.01978
gnl|onekp|VVRN_scaffold_2097676_Lonchitis_hirsuta 0.13406
gnl|onekp|FQGQ_scaffold_2004316_Polystichum_acrostichoides 0.10972
gnl|onekp|IXLH_scaffold_2011925_Polypodium_hesperium 0.08192
gnl|onekp|IXLH_scaffold_2001535_Polypodium_hesperium 0.02917
gnl|onekp|YLJA_scaffold_2002049_Polypodium_amorphum 0.02965
gnl|onekp|CJNT_scaffold_2008960_Polypodium_glycyrrhiza 0.02572
gnl|onekp|ZQYU_scaffold_2008427_Polypodium_plectolens 0.00477
gnl|onekp|ZQYU_scaffold_2008428_Polypodium_plectolens 0.00828
gnl|onekp|YQEC_scaffold_2003969_Woodsia_ilvensis 0.08194
gnl|onekp|YJJY_scaffold_2074562_Woodsia_scopulina 0.08085
gnl|onekp|HEGQ_scaffold_2000244_Gymnocarpium_dryopteris 0.01042
gnl|onekp|HEGQ_scaffold_2000245_Gymnocarpium_dryopteris 0.01257
gnl|onekp|HTFH_scaffold_2002637_Onoclea_sensibilis 0.05775
gnl|onekp|AFPO_scaffold_2008695_Blechnum_spicant 0.05541
gnl|onekp|VITX_scaffold_2009405_Blechnum_spicant 0.07363
gnl|onekp|OCZL_scaffold_2058438_Homalosorus_pycnocarpos 0.10636
gnl|onekp|WCLG_scaffold_2064902_Adiantum_aleuticum 0.11419
gnl|onekp|VITX_scaffold_2011306_Blechnum_spicant 0.05358
NbHCT3 0.05594
gnl|onekp|HTFH_scaffold_2006065_Onoclea_sensibilis 0.09302
gnl|onekp|HNDZ_scaffold_2027029_Cystopteris_utahensis 0.05099
gnl|onekp|LHLE_scaffold_2007869_Cystopteris_fragilis -0.00032
gnl|onekp|LHLE_scaffold_2007870_Cystopteris_fragilis 0.00032
gnl|onekp|RICC_scaffold_2012111_Cystopteris_reevesiana 0.00980
gnl|onekp|YOWV_scaffold_2068470_Cystopteris_protrusa 0.00120
gnl|onekp|XXHP_scaffold_2088283_Cystopteris_fragilis 0.00121
Cyatheales
Polypodiales
Cyatheales
Polypodiales
OsHCT_XM_015786263.1 0.28727
PvHCTLike1_AFY17066.1 0.30222
G10_Aagr_000038l.49.1 0.24206
AtDCF_BT010448.1 0.11634
AtFACT_AY142537.1 0.10954
AtASFT/HHT1_AY062996.1 0.13968
PtFHT1_JX515962.1 0.11643
StFHT_NM_001288261.1 0.11737
MeHFT_MeHFT 0.10393
MpolFHT_Mapoly0157s0023.1.p 0.10245
G3_Ppat_Pp3c14_6190V3.1.p 0.23451
G5_Sfal_Sphfalx08G078400.1.p 0.24952
AtSCT_BT009686.1 0.22721
(b)
MlAT6
PfRAS
LanAAT1_DQ886904.1
LaRAS
OtRAS
PsRAS_AM283092.1
CbRAS
GhRAS1
GhRAS2
SmRAS
MoRAS_FR670523.1
MoRAS
DcHCBT2_Z84386
PvHHHT_KX443573.1
TfHCT2_EU861219.1
ArHPT1_QAA12830.1
PcRAS
CiSHT1_MG457243.1
CiSHT2_MG457244.1
NtSHT_MN787045
SlSHT_MN787044
AtSHT_AT2G19070.1
VvSHT_MN787047
RicSHT_MN787043
RocSHT_MN787046
SgRAS
NbHCT3

Figure S10 Phylogenetic analysis of NbHCT3 orthologs across plants and known BAHDs (Data sets A and B in Supporting information datasets). NbHCT3 is marked with a red arrow. (**a**) Cutout of the phylogenetic analysis of NbHCT3 orthologs including all plants. The plant orders are marked on the right side. (**b**) Cutout of the phylogenetic analysis of NbHCT3 orthologs including all plants excluding Cyatheales and Polypodiales (negative control).

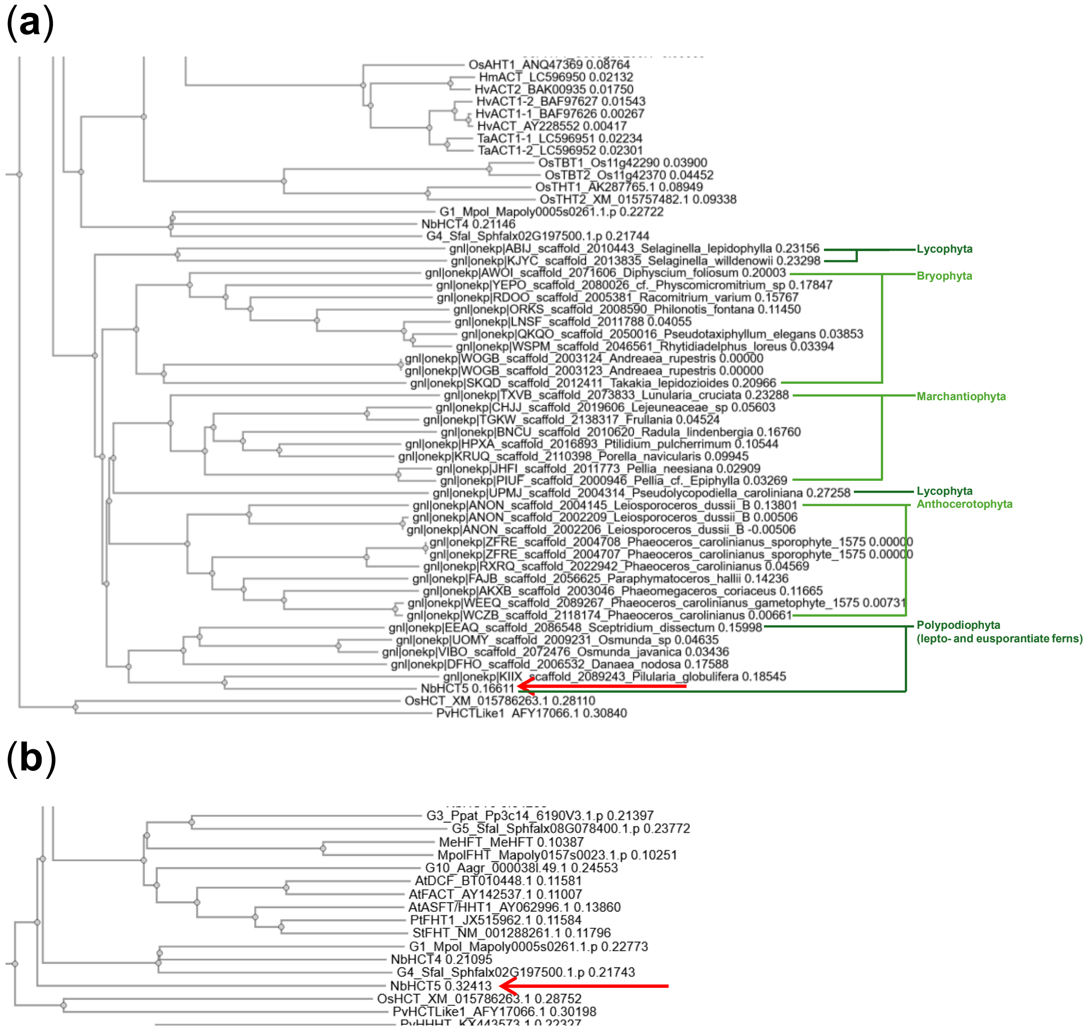

Figure S11 Phylogenetic analysis of NbHCT5 orthologs across plants and known BAHDs (Data sets C and D in Supporting information datasets). NbHCT5 is marked with a red arrow. (**a**) Cutout of the phylogenetic analysis of NbHCT5 orthologs including all plants. The plant diversions are marked on the right side. The sequences above the marked sequences belong to clade IV and indicate a closer relationship to clade IV. (**b**) Cutout of the phylogenetic analysis of NbHCT5 orthologs including all plants excluding Lycophyta, Bryophyta, Marchantiophyta, Anthocerotophyta and Polypodiaphyta (negative control).

Supporting Tables

Table S1 BLASTP search in the transcriptome of *Struthiopteris spicant* (1kP database VITX) with the amino acid sequence of rosmarinic acid synthase (RAS) from *Coleus blumei* (syn. *Coleus scutellarioides*, UniProt A0PDV5; Berger *et al.*, 2006) as query. Note: alignment length does not refer to scaffold length; aa = amino acids; cutoff value: E = $1.0 \cdot 10^{-50}$. Scaffold 2003763 was added to the list because of the presence of a lysine-handle (according to Levsh *et al.*, 2019).

Identifier	Identity [%]	Alignment length [aa]	E-value	Template for
VITX_scaffold_2011375	45	470	$5.39 \cdot 10^{-140}$	*NbHCT2*
VITX_scaffold_2009405	38	448	$5.64 \cdot 10^{-95}$	
VITX_scaffold_2011306	37	441	$1.07 \cdot 10^{-86}$	*NbHCT3*
VITX_scaffold_2010977	34	438	$3.58 \cdot 10^{-75}$	
VITX_scaffold_2015342	33	438	$9.57 \cdot 10^{-75}$	
VITX_scaffold_2009524	37	384	$8.06 \cdot 10^{-74}$	*NbHCT4*
VITX_scaffold_2014301	36	424	$3.29 \cdot 10^{-71}$	*NbHCT5*
VITX_scaffold_2010590	33	410	$2.11 \cdot 10^{-67}$	
VITX_scaffold_2007085	46	217	$2.49 \cdot 10^{-63}$	*NbHCT1*
VITX_scaffold_2007085	46	213	$2.16 \cdot 10^{-58}$	
VITX_scaffold_2099633	44	270	$1.14 \cdot 10^{-59}$	*NbHCT8*
VITX_scaffold_2006651	30	446	$1.95 \cdot 10^{-56}$	
VITX_scaffold_2099069	30	438	$4.77 \cdot 10^{-53}$	
VITX_scaffold_2003763	29	431	$4.15 \cdot 10^{-47}$	*NbHCT9*

Table S2 Screened putative NbHCT substrates and method of preparation and detection. Methods A and B are explained in the main text. The term “aliphatic or aromatic alcohol/amine” refers to the carbon atom that bears an alcohol or amine group.

Substrate	Stock solution used in assays	Method of preparation for LC/ESI-MS/MS	Ionization
Acyl acceptors – shikimic acid pathway derivatives			
quinic acid	1 M pH 7.0	method A	negative mode
shikimic acid	1 M pH 7.0	method A	negative mode
chlorogenic acid + quinic acid	25 mM / 1 M pH 7.0	method A	negative mode
D/L-phenylalanine	100 mM	method A	positive mode
D/L-tyrosine	100 mM	method B	positive mode
DL-DOPA	100 mM	method B	positive mode
Acyl acceptors – 4-hydroxyphenyllactic acid derivatives and precursors			
D/L-3-phenyllactic acid	1 M pH 7.0	method A	negative mode
DL/L-4-hydroxyphenyllactic acid	100 mM	method A	negative mode
DL-3,4-dihydroxyphenyllactic acid	100 mM	method A	negative mode
piscidic acid	38 mM	method A	negative mode
Acyl acceptors – aliphatic alcohols			
glycerol	100 mM	method A	positive mode
methanol	100 mM	method A	positive mode
ethanol	100 mM	method A	positive mode
iso-propanol	100 mM	method A	positive mode
n-propanol	100 mM	method A	positive mode
butanol	100 mM	method A	positive mode
ω-hydroxypalmitic acid	50 mM pH 7.3 10 % ethanol	method A	negative mode
coniferyl alcohol	100 mM	method A	positive mode
benzyl alcohol	1 M pH 7.0	method A	positive mode
4-hydroxybenzyl alcohol	100 mM	method A	positive mode
4-isopropylbenzyl alcohol	100 mM	method A	positive mode
1-phenylethanol	100 mM	method A	positive mode
2-phenylethanol	undiluted	method A	positive mode
2-(4-hydroxyphenyl-)ethanol	100 mM	method A	positive mode
3-phenylpropanol	pure	method A	positive mode
3-(4-hydroxyphenyl-)propanol	100 mM	method A	positive mode

4-phenylbutanol	pure	method A	positive mode
D-glucose	1 M pH 7.0	method A	positive mode
D-galactose	1 M pH 7.0	method A	positive mode
D/L-threonic acid	10 mM	method A	negative mode
mucic acid	10 mM	method A	negative mode
D-glucaric acid	100 mM	method A	negative mode
D/L-tartaric acid	100 mM	method A	negative mode
meso-tartaric acid	100 mM	method A	negative mode
D/L-malic acid	100 mM	method A	negative mode
Acyl acceptors – aromatic alcohols and amines			
2-hydroxybenzoic acid	100 mM	method A	negative mode
3-hydroxybenzoic acid	100 mM	method A	negative mode
4-hydroxybenzoic acid	100 mM	method A	negative mode
2-aminobenzoic acid	100 mM	method A	negative mode
3-aminobenzoic acid	100 mM	method A	negative mode
4-aminobenzoic acid	100 mM	method A	negative mode
2,3-dihydroxybenzoic acid	100 mM	method A	negative mode
2,4-dihydroxybenzoic acid	100 mM	method A	negative mode
2,5-dihydroxybenzoic acid	100 mM	method A	negative mode
3,4-dihydroxybenzoic acid	100 mM	method A	negative mode
3-hydroxyanthranilic acid	75 mM pH 7.0	method A	negative mode
5-hydroxyanthranilic acid	75 mM pH 7.0	method A	negative mode
3-aminosalicylic acid	100 mM	method A	negative mode
vanillic acid	100 mM	method A	negative mode
homogentisic acid	100 mM	method A	negative mode
4-hydroxyphenylacetic acid	100 mM	method A	negative mode
Acyl acceptors – aliphatic amines			
putrescine	96 mM pH 7.0	method B	positive mode
spermidine	63.5 mM pH 7.0	method B	positive mode
spermine	9.19 mM pH 7.0	method B	positive mode
agmatine	96.4 mM pH 7.0	method B	positive mode
2-phenylethylamine	20 mM	method B	positive mode
tyramine	9.5 mM pH 7.0	method B	positive mode
dopamine	100 mM	method B	positive mode
tryptamine	97.5 mM pH 7.0	method B	positive mode
serotonin	95 mM pH 7.0	method B	positive mode
D/L-tryptophan	40 mM	method B	positive mode

L-glutamine	50 mM	method B	positive mode
Acyl donors			
cinnamoyl-CoA	2.5 mM	depending on acyl acceptor	
p-coumaroyl-CoA	2.5 mM	depending on acyl acceptor	
caffeoyl-CoA	2.5 mM	depending on acyl acceptor	
feruloyl-CoA	2.5 mM	depending on acyl acceptor	
sinapoyl-CoA	2.5 mM	depending on acyl acceptor	
benzoyl-CoA	2.5 mM	depending on acyl acceptor	
acetyl-CoA	2.5 mM	depending on acyl acceptor	

Table S3 Investigated NbHCTs and their transformed acyl donors and acyl acceptors. Acyl acceptors were tested together with the respective acyl donors written in bold. Acyl donors were tested together with the respective acyl acceptor written in bold. Product verification (see Table S4-S12) was performed against negative controls. Note that due to the high amount of enzyme, substrates and the long incubation time, less accepted substrates still give respectable peaks. Peak intensities of the proposed products that were below 1.0·10^{3} counts, be it due to low amounts or poor ionization, are considered traces (written in italics) and are not broken down in Tables S4-S12.

Protein	Accepted acyl donors	Accepted acyl acceptors
NbHCT1	cinnamoyl-CoA, ***p*-coumaroyl-CoA**, caffeoyl-CoA, feruloyl-CoA, sinapoyl-CoA, benzoyl-CoA	**shikimic acid**, quinic acid, L-threonic acid, *D-threonic acid*, 3-aminobenzoic acid, *2,3-dihydroxybenzoic acid*, 2,5-dihydroxybenzoic acid, *3,4-dihydroxybenzoic acid*, 3-hydroxyanthranilic acid, *5-hydroxyanthranilic acid*, homogentisic acid, 1-phenylethanol, tryptamine
NbHCT2	cinnamoyl-CoA, ***p*-coumaroyl-CoA**, caffeoyl-CoA, feruloyl-CoA, sinapoyl-CoA, benzoyl-CoA	**shikimic acid**, quinic acid, L-threonic acid, *D-threonic acid*, 3-aminobenzoic acid, 2,3-dihydroxybenzoic acid, 2,5-dihydroxybenzoic acid, 3,4-dihydroxybenzoic acid, 3-hydroxyanthranilic acid, 5-hydroxyanthranilic acid, homogentisic acid, 1-phenylethanol, tryptamine
NbHCT3	cinnamoyl-CoA, ***p*-coumaroyl-CoA**, caffeoyl-CoA, feruloyl-CoA, sinapoyl-CoA, benzoyl-CoA	**quinic acid**, shikimic acid, *L-threonic acid*, *D-threonic acid*, *2,3-dihydroxybenzoic acid*, 2,5-dihydroxybenzoic acid, 3-hydroxyanthranilic acid, homogentisic acid, 1-phenylethanol, tryptamine
NbHCT4	***p*-coumaroyl-CoA**	*L-threonic acid*, *D-threonic acid*, *2,5-dihydroxybenzoic acid*, homogentisic acid, 1-phenylethanol, tryptamine
NbHCT5	cinnamoyl-CoA, ***p*-coumaroyl-CoA**, caffeoyl-CoA, feruloyl-CoA, sinapoyl-CoA, benzoyl-CoA	**tryptamine**, homogentisic acid, *L-threonic acid*, *D-threonic acid*, *2,5-dihydroxybenzoic acid*, 1-phenyl-ethanol,
NbHCT6	***p*-coumaroyl-CoA**	*D-threonic acid*, *2,5-dihydroxybenzoic acid*, homogent-isic acid, 1-phenylethanol, tryptamine
NbHCT7	***p*-coumaroyl-CoA**	*D-threonic acid*, *2,5-dihydroxybenzoic acid*, homo-gentisic acid, 1-phenylethanol, tryptamine
NbHCT8	cinnamoyl-CoA, ***p*-coumaroyl-CoA**, caffeoyl-CoA, feruloyl-CoA, sinapoyl-CoA, benzoyl-CoA, acetyl-CoA	**3-hydroxyanthranilic acid**, shikimic acid, quinic acid, L-threonic acid, D-threonic acid, 3-aminobenzoic acid, 2,3-dihydroxybenzoic acid, 2,5-dihydroxybenzoic acid, 3,4-dihydroxybenzoic acid, 5-hydroxyanthranilic acid, homogentisic acid, 1-phenylethanol, tryptamine
NbHCT9	***p*-coumaroyl-CoA**	*L-threonic acid*, *D-threonic acid*, *2,5-dihydroxybenzoic acid*, homogentisic acid, 1-phenylethanol, tryptamine

Table S4 Summary of identified products formed in NbHCT1 assays and resulting LC/ESI-MS and LC/ESI-MS/MS data (negative (**A**) or positive (**B**) ionization mode), *m/z* calculated with Compass DataAnalysis, products with less than $1.0 \cdot 10^3$ counts are considered traces and not listed, only fragments >10 % base peak are listed. * Compared with external authentic standard.

**A**<br>**Detected product**	**Molecular formula**	**Retention time [min]**	**Calculated *m/z* [M-H]$^-$**	**Measured *m/z* [M-H]$^-$**	**Peak intensity m/z [M-H]$^-$ [counts]**	**LC/ESI-MS/MS *m/z* (% base peak)**
cinnamoyl-5-*O*-shikimic acid	$C_{16}H_{16}O_6$	8.9	303.0863	303.1297	$1.2 \cdot 10^5$	MS2[303]: 147 (100), 137 (58)
p-coumaroyl-4-*O*-shikimic acid	$C_{16}H_{16}O_7$	7.6	319.0812	319.0918	$1.1 \cdot 10^3$	no MS2 event
p-coumaroyl-5-*O*-shikimic acid *	$C_{16}H_{16}O_7$	7.9	319.0812	319.0940	$1.3 \cdot 10^4$	MS2[319]: 163 (60), 145 (26), 137 (15), 119 (100), 117 (22)
caffeoyl-4-*O*-shikimic acid *	$C_{16}H_{16}O_8$	7.1	335.0761	335.1192	$4.8 \cdot 10^3$	MS2[335]: 179 (15), 161 (100), 135 (19), 133 (13)
caffeoyl-5-*O*-shikimic acid *	$C_{16}H_{16}O_8$	7.2	335.0761	335.1214	$1.2 \cdot 10^5$	MS2[335]: 179 (100), 161 (44), 135 (98)
feruloyl-5-*O*-shikimic acid	$C_{17}H_{18}O_8$	7.8	349.0918	349.1380	$6.1 \cdot 10^4$	MS2[349]: 193 (92), 178 (51), 175 (29), 160 (28), 137 (28), 134 (100)
sinapoyl-5-*O*-shikimic acid	$C_{18}H_{20}O_9$	7.7	379.1024	379.1489	$1.1 \cdot 10^4$	MS2[379]: 223 (74), 208 (67), 164 (100)
benzoyl-5-*O*-shikimic acid	$C_{14}H_{14}O_6$	8.2	277.0707	277.1120	$3.1 \cdot 10^3$	no MS2 event

p-coumaroyl-5-*O*-quinic acid *	$C_{16}H_{18}O_8$	7.3	337.0918	337.1048	1.1·10^4	MS2[337]: 191 (100), 163 (14)
ester of *p*-coumaric acid and L-threonic acid	$C_{13}H_{14}O_7$	7.5	281.0656	281.1049	2.3·10^3	no MS2 event
p-coumaroyl-*N*-3-aminobenzoic acid	$C_{16}H_{13}O_4N$	9.1	282.0761	282.1167	4.7·10^4	MS2[282]: 145 (100), 136 (14), 119 (58), 117 (40)
p-coumaroyl-*N*-3-hydroxyanthranilic acid *	$C_{16}H_{13}O_5N$	10.1	298.0710	298.0801	2.5·10^4	MS2[298]: 236 (68), 160 (14), 145 (77), 134 (43), 119 (100), 117 (22), 108 (15)
ester of *p*-coumaric acid and 2,5-dihydroxybenzoic acid	$C_{16}H_{12}O_6$	10.1	299.0550	299.0945	1.8·10^4	MS2[299]: 153 (35), 145 (100), 117 (35), 109 (36)
p-coumaroyl-2-*O*-homogentisic acid and *p*-coumaroyl-5-*O*-homogentisic acid	$C_{17}H_{14}O_6$	8.5	313.0707	313.1114	8.5·10^3	MS2[313]: 227 (100)
		9.1	313.0707	313.1113	1.2·10^4	MS2[313]: 145 (100), 122 (17), 117 (28)
**B Detected product**	**Molecular formula**	**Retention time [min]**	**Calculated *m/z* [M+H]⁺**	**Measured *m/z* [M+H]⁺**	**Peak intensity m/z [M+H]⁺ [counts]**	**LC/ESI-MS/MS *m/z* (% base peak)**
p-coumaroyl-1-phenylethanolate	$C_{17}H_{16}O_3$	11.9	269.1172	269.1116	2.0·10^3	no MS2 event
p-coumaroyl-tryptamine	$C_{19}H_{18}O_2N_2$	10.0	307.1441	307.1432	1.8·10^3	no MS2 event

Table S5 Summary of identified products formed in NbHCT2 assays and resulting LC/ESI-MS and LC/ESI-MS/MS data (negative (**A**) or positive (**B**) ionization mode), *m/z* calculated with Compass DataAnalysis, products with less than $1.0 \cdot 10^3$ counts are considered traces and not listed, only fragments >10 % base peak are listed. * Compared with external authentic standard.

**A**<br>**Detected product**	**Molecular formula**	**Retention time [min]**	**Calculated *m/z* [M-H]$^-$**	**Measured *m/z* [M-H]$^-$**	**Peak intensity m/z [M-H]$^-$ [counts]**	**LC/ESI-MS/MS *m/z* (% base peak)**
cinnamoyl-5-*O*-shikimic acid	$C_{16}H_{16}O_6$	8.9	303.0863	303.1308	$1.2 \cdot 10^5$	MS2[303]: 147 (100), 137 (85)
p-coumaroyl-4-*O*-shikimic acid	$C_{16}H_{16}O_7$	7.7	319.0812	319.039	$1.0 \cdot 10^3$	no MS2 event
p-coumaroyl-5-*O*-shikimic acid *	$C_{16}H_{16}O_7$	7.9	319.0812	319.0931	$1.2 \cdot 10^4$	MS2[319]: 163 (63), 145 (30), 137 (11), 119 (100), 117 (15)
caffeoyl-4-*O*-shikimic acid *	$C_{16}H_{16}O_8$	7.0	335.0761	335.1200	$1.2 \cdot 10^4$	MS2[335]: 179 (23), 161 (100), 135 (26), 133 (16)
caffeoyl-5-*O*-shikimic acid *	$C_{16}H_{16}O_8$	7.2	335.0761	335.1218	$1.2 \cdot 10^5$	MS2[335]: 179 (100), 161 (46), 135 (98)
feruloyl-5-*O*-shikimic acid	$C_{17}H_{18}O_8$	7.8	349.0918	349.1377	$1.0 \cdot 10^5$	MS2[349]: 193 (85), 178 (49), 175 (32), 160 (26), 137 (25), 134 (100)
sinapoyl-5-*O*-shikimic acid	$C_{18}H_{20}O_9$	7.7	379.1024	379.1467	$2.5 \cdot 10^3$	no MS2 event
benzoyl-5-*O*-shikimic acid	$C_{14}H_{14}O_6$	8.2	277.0707	277.1118	$6.6 \cdot 10^3$	MS2[277]: 162 (19), 121 (100)

p-coumaroyl-5-*O*-quinic acid *	$C_{16}H_{18}O_8$	7.3	337.0918	337.1042	$1.2 \cdot 10^4$	MS2[337]: 191 (100), 163 (15)
ester of *p*-coumaric acid and L-threonic acid	$C_{13}H_{14}O_7$	7.5	281.0656	281.1059	$4.4 \cdot 10^3$	MS2[281]: 163 (73), 151 (10), 119 (100)
p-coumaroyl-*N*-3-aminobenzoic acid	$C_{16}H_{13}O_4N$	9.1	282.0761	282.1164	$7.5 \cdot 10^4$	MS2[282]: 145 (100), 136 (13), 119 (56), 117 (42)
p-coumaroyl-*N*-3-hydroxyanthranilic acid *	$C_{16}H_{13}O_5N$	10.1	298.0710	298.0799	$2.9 \cdot 10^4$	MS2[298]: 236 (88), 163 (17), 160 (19), 145 (100), 134 (55), 119 (96), 117 (29), 108 (12)
p-coumaroyl-*O*-5-hydroxyanthranilic acid *	$C_{16}H_{13}O_5N$	9.0	298.0710	298.0786	$1.7 \cdot 10^4$	MS2[298]: 254 (61), 236 (12), 226 (44), 212 (32), 160 (87), 145 (20), 134 (100), 119 (19)
ester of *p*-coumaric acid and 2,3-dihydroxybenzoic acid	$C_{16}H_{12}O_6$	10.1	299.0550	299.0941	$1.6 \cdot 10^4$	MS2[299]: 153 (100), 145 (33), 117 (16), 109 (87)
ester of *p*-coumaric acid and 2,5-dihydroxybenzoic acid	$C_{16}H_{12}O_6$	10.1	299.0550	299.0953	$4.8 \cdot 10^4$	MS2[299]: 153 (26), 145 (100), 117 (36), 109 (43)
p-coumaroyl-3-*O*-3,4-dihydroxybenzoic acid and *p*-coumaroyl-4-*O*-	$C_{16}H_{12}O_6$	9.0	299.0550	299.0937	$4.1 \cdot 10^4$	MS2[299]: 153 (100), 145 (50), 117 (10), 109 (81)

3,4-dihydroxy-benzoic acid		9.2	299.0550	299.0931	$1.2 \cdot 10^3$	no MS2 event
p-coumaroyl-2-*O*-homogentisic acid and *p*-coumaroyl-5-*O*-homogentisic acid	$C_{17}H_{14}O_6$	8.5	313.0707	313.1103	$1.8 \cdot 10^3$	no MS2 event
		9.1	313.0707	313.1113	$2.1 \cdot 10^4$	MS2[313]: 145 (100), 122 (24), 117 (29)
**B Detected product**	**Molecular formula**	**Retention time [min]**	**Calculated *m/z* [M+H]$^+$**	**Measured *m/z* [M+H]$^+$**	**Peak intensity m/z [M+H]$^+$ [counts]**	**LC/ESI-MS/MS *m/z* (% base peak)**
p-coumaroyl-1-phenylethanolate	$C_{17}H_{16}O_3$	11.9	269.1172	269.1142	$1.9 \cdot 10^3$	no MS2 event
p-coumaroyl-tryptamine	$C_{19}H_{18}O_2N_2$	10.0	307.1441	307.1363	$3.4 \cdot 10^3$	MS2[307]: 147 (100), 144 (19), 119 (19)

Table S6 Summary of identified products formed in NbHCT3 assays and resulting LC/ESI-MS and LC/ESI-MS/MS data (negative (**A**) or positive (**B**) ionization mode), *m/z* calculated with Compass DataAnalysis, products with less than $1.0 \cdot 10^3$ counts are considered traces and not listed, only fragments >10 % base peak are listed. * Compared with external authentic standard.

**A**<br>**Detected product**	**Molecular formula**	**Retention time [min]**	**Calculated *m/z* [M-H]$^-$**	**Measured *m/z* [M-H]$^-$**	**Peak intensity m/z [M-H]$^-$ [counts]**	**LC/ESI-MS/MS *m/z* (% base peak)**
cinnamoyl-5-*O*-quinic acid	$C_{16}H_{18}O_7$	8.4	321.0969	303.1414	$2.7 \cdot 10^5$	MS2[321]: 173 (61), 147 (100)
p-coumaroyl-5-*O*-quinic acid *	$C_{16}H_{18}O_8$	7.3	337.0918	337.1050	$1.1 \cdot 10^4$	MS2[337]: 191 (100), 163 (16)
caffeoyl-5-*O*-quinic acid *	$C_{16}H_{18}O_9$	6.6	353.0867	353.1325	$9.2 \cdot 10^4$	MS2[335]: 191 (100)
feruloyl-5-*O*-quinic acid	$C_{17}H_{20}O_9$	7.3	367.1024	367.1495	$1.3 \cdot 10^5$	MS2[349]: 191 (100)
sinapoyl-5-*O*-quinic acid	$C_{18}H_{22}O_{10}$	7.2	397.1129	397.1612	$7.3 \cdot 10^4$	MS2[397]: 223 (20), 191 (100)
benzoyl-5-*O*-quinic acid	$C_{14}H_{16}O_7$	7.5	295.0812	295.1241	$1.0 \cdot 10^5$	MS2[295]: 173 (42), 121 (100)
p-coumaroyl-4-*O*-shikimic acid	$C_{16}H_{16}O_7$	7.6	319.0812	319.0929	$4.8 \cdot 10^3$	MS2[319]: 163 (10), 145 (100), 119 (19), 117 (38)
p-coumaroyl-5-*O*-shikimic acid *	$C_{16}H_{16}O_7$	7.9	319.0812	319.0935	$6.2 \cdot 10^3$	MS2[319]: 163 (76), 145 (20), 137 (17), 119 (100), 117 (20)
p-coumaroyl-*N*-3-hydroxyanthranilic acid *	$C_{16}H_{13}O_5N$	10.1	298.0710	298.0783	$1.5 \cdot 10^3$	no MS2 event

ester of *p*-coumaric acid and 2,5-dihydroxybenzoic acid	$C_{16}H_{12}O_6$	10.1	299.0550	299.0945	$4.0 \cdot 10^4$	MS2[299]: 153 (25), 145 (100), 117 (30), 109 (35)
p-coumaroyl-2-*O*-homogentisic acid and *p*-coumaroyl-5-*O*-homogentisic acid	$C_{17}H_{14}O_6$	8.5	313.0707	313.1101	$2.2 \cdot 10^3$	no MS2 event
		9.1	313.0707	313.1100	$2.5 \cdot 10^3$	no MS2 event
**B Detected product**	**Molecular formula**	**Retention time [min]**	**Calculated *m/z* [M+H]+**	**Measured *m/z* [M+H]+**	**Peak intensity m/z [M+H]+ [counts]**	**LC/ESI-MS/MS *m/z* (% base peak)**
p-coumaroyl-1-phenylethanolate	$C_{17}H_{16}O_3$	11.9	269.1172	269.1182	$7.7 \cdot 10^2$	no MS2 event
p-coumaroyl-tryptamine	$C_{19}H_{18}O_2N_2$	10.0	307.1441	307.1385	$1.4 \cdot 10^3$	no MS2 event

Table S7 Summary of identified products formed in NbHCT8 assays and resulting LC/ESI-MS and LC/ESI-MS/MS data (negative (**A**) or positive (**B**) ionization mode), *m/z* calculated with Compass DataAnalysis, products with less than $1.0 \cdot 10^3$ counts are considered traces and not listed, only fragments >10 % base peak are listed. * Compared with external authentic standard.

**A**<br>**Detected product**	**Molecular formula**	**Retention time [min]**	**Calculated *m/z* [M-H]$^-$**	**Measured *m/z* [M-H]$^-$**	**Peak intensity m/z [M-H]$^-$ [counts]**	**LC/ESI-MS/MS *m/z* (% base peak)**
cinnamoyl-*N*-3-hydroxyanthranilic acid	$C_{16}H_{13}O_4N$	11.2	282.0761	282.1186	$3.5 \cdot 10^5$	MS2[282]: 238 (18), 220 (68), 210 (38), 134 (100)
p-coumaroyl-*N*-3-hydroxyanthranilic acid *	$C_{16}H_{13}O_5N$	10.1	298.0710	298.0798	$2.9 \cdot 10^4$	MS2[298]: 236 (92), 160 (10), 145 (85), 134 (53), 119 (100), 117 (19), 108 (17)
caffeoyl-*N*-3-hydroxyanthranilic acid	$C_{16}H_{13}O_6N$	9.4	314.0659	314.1086	$5.7 \cdot 10^4$	MS2[314]: 252 (10), 161 (82), 152 (18), 135 (100)
feruloyl-*N*-3-hydroxyanthranilic acid	$C_{17}H_{15}O_6N$	10.1	328.0816	328.1259	$6.1 \cdot 10^4$	MS2[328]: 251 (30), 175 (66), 161 (28), 169 (56), 134 (100)
sinapoyl-*N*-3-hydroxyanthranilic acid	$C_{18}H_{17}O_7N$	10.0	358.0921	358.1374	$4.0 \cdot 10^4$	MS2[358]: 266 (21), 205 (87), 190 (67), 179 (35), 164 (100), 149 (27), 134 (48)

benzoyl-*N*-3-hydroxyanthranilic acid	$C_{14}H_{11}O_4N$	10.5	256.0604	256.1005	$2.4 \cdot 10^4$	MS2[256]: 212 (41), 194 (22), 134 (100)
p-coumaroyl-*N*-5-hydroxyanthranilic acid *	$C_{16}H_{13}O_5N$	9.6	298.0710	298.0779	$6.3 \cdot 10^3$	MS2[298]: 145 (100), 117 (35)
p-coumaroyl-5-*O*-quinic acid *	$C_{16}H_{18}O_8$	7.3	337.0918	337.1052	$1.3 \cdot 10^4$	MS2[337]: 191 (100), 163 (14)
p-coumaroyl-4-*O*-shikimic acid	$C_{16}H_{16}O_7$	7.6	319.0812	319.0929	$1.3 \cdot 10^3$	no MS2 event
p-coumaroyl-5-*O*-shikimic acid *	$C_{16}H_{16}O_7$	7.9	319.0812	319.0929	$1.3 \cdot 10^4$	MS2[319]: 163 (76), 145 (36), 137 (12), 119 (100), 117 (16)
ester of *p*-coumaric acid and D-threonic acid	$C_{13}H_{14}O_7$	7.5	281.0656	281.1066	$1.3 \cdot 10^3$	no MS2 event
ester of *p*-coumaric acid and L-threonic acid	$C_{13}H_{14}O_7$	7.5	281.0656	281.1048	$2.1 \cdot 10^4$	MS2[281]: 163 (56), 119 (100)
p-coumaroyl-*N*-3-aminobenzoic acid	$C_{16}H_{13}O_4N$	9.1	282.0761	282.1150	$1.8 \cdot 10^4$	MS2[282]: 145 (100), 119 (60), 117 (50)
ester of *p*-coumaric acid and 2,3-dihydroxybenzoic acid	$C_{16}H_{12}O_6$	10.1	299.0550	299.0949	$3.8 \cdot 10^4$	MS2[299]: 153 (100), 145 (32), 117 (12), 109 (71)
ester of *p*-coumaric acid and 2,5-dihydroxybenzoic acid	$C_{16}H_{12}O_6$	10.1	299.0550	299.0941	$7.7 \cdot 10^4$	MS2[299]: 153 (22), 145 (100), 117 (36), 109 (33)
p-coumaroyl-3-*O*-3,4-dihydroxybenzoic acid and *p*-coumaroyl-4-*O*-3,4-dihydroxybenzoic acid	$C_{16}H_{12}O_6$	9.0	299.0550	299.0943	$1.3 \cdot 10^4$	MS2[299]: 153 (100), 145 (42), 117 (10), 109 (81)
		9.2	299.0550	299.0938	$1.2 \cdot 10^3$	no MS2 event

p-coumaroyl-2-*O*-homogentisic acid and *p*-coumaroyl-5-*O*-homogentisic acid	$C_{17}H_{14}O_6$	8.5	313.0707	313.1097	$6.8 \cdot 10^3$	MS2[313]: 227 (100)
		9.1	313.0707	313.1095	$4.3 \cdot 10^3$	MS2[313]: 145 (100), 122 (24), 117 (37)
**B Detected product**	**Molecular formula**	**Retention time [min]**	**Calculated *m/z* [M+H]⁺**	**Measured *m/z* [M+H]⁺**	**Peak intensity m/z [M+H]⁺ [counts]**	**LC/ESI-MS/MS *m/z* (% base peak)**
p-coumaroyl-1-phenylethanolate	$C_{17}H_{16}O_3$	11.9	269.1172	269.1142	$3.1 \cdot 10^3$	MS2[269]: 165 (27), 147 (100), 119 (13), 105 (14)
p-coumaroyl-tryptamine	$C_{19}H_{18}O_2N_2$	10.0	307.1441	307.1423	$2.5 \cdot 10^4$	MS2[307]: 147 (100), 144 (13), 119 (15)

Table S8 Summary of identified products formed in NbHCT5 assays and resulting LC/ESI-MS and LC/ESI-MS/MS data (negative (**A**) or positive (**B**) ionization mode), *m/z* calculated with Compass DataAnalysis, products with less than $1.0 \cdot 10^3$ counts are considered traces and not listed, only fragments >10 % base peak are listed.

**A**<br>**Detected product**	**Molecular formula**	**Retention time [min]**	**Calculated *m/z* [M-H]$^-$**	**Measured *m/z* [M-H]$^-$**	**Peak intensity m/z [M-H]$^-$ [counts]**	**LC/ESI-MS/MS *m/z* (% base peak)**
p-coumaroyl-2-*O*-homogentisic acid *or p*-coumaroyl-5-*O*-homogentisic acid	$C_{17}H_{14}O_6$	8.5	313.0707	313.1103	$4.8 \cdot 10^3$	MS2[313]: 227 (100)
**B**<br>**Detected product**	**Molecular formula**	**Retention time [min]**	**Calculated *m/z* [M+H]$^+$**	**Measured *m/z* [M+H]$^+$**	**Peak intensity m/z [M+H]$^+$ [counts]**	**LC/ESI-MS/MS *m/z* (% base peak)**
cinnamoyl-tryptamine	$C_{19}H_{18}ON_2$	11.1	291.1492	291.1527	$6.3 \cdot 10^5$	MS2[291]: 274 (11), 144 (68), 131 (100)
p-coumaroyl-tryptamine	$C_{19}H_{18}O_2N_2$	10.0	307.1441	307.1451	$1.1 \cdot 10^5$	MS2[307]: 147 (100), 144 (10), 119 (10)
caffeoyl-tryptamine	$C_{19}H_{18}O_3N_2$	9.4	323.1390	323.1417	$1.8 \cdot 10^5$	MS2[323]: 163 (100), 144 (29)
feruloyl-tryptamine	$C_{20}H_{20}O_3N_2$	9.9	337.1547	337.1572	$2.1 \cdot 10^5$	MS2[337]: 177 (100)
sinapoyl-tryptamine	$C_{21}H_{22}O_4N_2$	9.8	367.1652	367.1655	$6.0 \cdot 10^4$	MS2[367]: 207 (100), 175 (15)
benzoyl-tryptamine	$C_{17}H_{16}ON_2$	10.6	265.1335	265.1336	$1.3 \cdot 10^5$	MS2[265]: 144 (100)
p-coumaroyl-1-phenyl-ethanolate	$C_{17}H_{16}O_3$	11.9	269.1172	269.1170	$1.5 \cdot 10^3$	no MS2 event

Table S9 Summary of identified products formed in NbHCT4 assays and resulting LC/ESI-MS and LC/ESI-MS/MS data (negative (**A**) or positive (**B**) ionization mode), *m/z* calculated with Compass DataAnalysis, products with less than $1.0 \cdot 10^3$ counts are considered traces and not listed, only fragments >10 % base peak are listed.

**A**<br>**Detected product**	**Molecular formula**	**Retention time [min]**	**Calculated *m/z* [M-H]$^-$**	**Measured *m/z* [M-H]$^-$**	**Peak intensity m/z [M-H]$^-$ [counts]**	**LC/ESI-MS/MS *m/z* (% base peak)**
p-coumaroyl-2-*O*-homogentisic acid *or* *p*-coumaroyl-5-*O*-homogentisic acid	$C_{17}H_{14}O_6$	8.5	313.0707	313.1111	$4.2 \cdot 10^3$	MS2[313]: 227 (100)
**B**<br>**Detected product**	**Molecular formula**	**Retention time [min]**	**Calculated *m/z* [M+H]$^+$**	**Measured *m/z* [M+H]$^+$**	**Peak intensity m/z [M+H]$^+$ [counts]**	**LC/ESI-MS/MS *m/z* (% base peak)**
p-coumaroyl-1-phenylethanol-ate	$C_{17}H_{16}O_3$	11.9	269.1172	269.1110	$1.2 \cdot 10^3$	no MS2 event
p-coumaroyl-tryptamine	$C_{19}H_{18}O_2N_2$	10.0	307.1441	307.1460	$1.2 \cdot 10^3$	no MS2 event

Table S10 Summary of identified products formed in NbHCT6 assays and resulting LC/ESI-MS and LC/ESI-MS/MS data (negative (**A**) or positive (**B**) ionization mode), *m/z* calculated with Compass DataAnalysis, products with less than $1.0 \cdot 10^3$ counts are considered traces and not listed, only fragments >10 % base peak are listed.

**A**<br>**Detected product**	**Molecular formula**	**Retention time [min]**	**Calculated *m/z* [M-H]$^-$**	**Measured *m/z* [M-H]$^-$**	**Peak intensity m/z [M-H]$^-$ [counts]**	**LC/ESI-MS/MS *m/z* (% base peak)**
p-coumaroyl-2-*O*-homogentisic acid *or p*-coumaroyl-5-*O*-homogentisic acid	$C_{17}H_{14}O_6$	8.5	313.0707	313.1106	$7.8 \cdot 10^3$	MS2[313]: 227 (100)
**B**<br>**Detected product**	**Molecular formula**	**Retention time [min]**	**Calculated *m/z* [M+H]$^+$**	**Measured *m/z* [M+H]$^+$**	**Peak intensity m/z [M+H]$^+$ [counts]**	**LC/ESI-MS/MS *m/z* (% base peak)**
p-coumaroyl-1-phenylethanolate	$C_{17}H_{16}O_3$	11.9	269.1172	269.1145	$4.4 \cdot 10^3$	MS2[269]: 165 (26), 147 (100), 119 (13)
p-coumaroyl-tryptamine	$C_{19}H_{18}O_2N_2$	10.0	307.1441	307.1394	$1.0 \cdot 10^3$	no MS2 event

Table S11 Summary of identified products formed in NbHCT7 assays and resulting LC/ESI-MS and LC/ESI-MS/MS data (negative (**A**) or positive (**B**) ionization mode), *m/z* calculated with Compass DataAnalysis, products with less than $1.0 \cdot 10^3$ counts are considered traces and not listed, only fragments >10 % base peak are listed.

**A**<br>**Detected product**	**Molecular formula**	**Retention time [min]**	**Calculated *m/z* [M-H]$^-$**	**Measured *m/z* [M-H]$^-$**	**Peak intensity m/z [M-H]$^-$ [counts]**	**LC/ESI-MS/MS *m/z* (% base peak)**
p-coumaroyl-2-*O*-homogentisic acid *or p*-coumaroyl-5-*O*-homogentisic acid	$C_{17}H_{14}O_6$	8.5	313.0707	313.1097	$3.8 \cdot 10^3$	MS2[313]: 227 (100)
**B**<br>**Detected product**	**Molecular formula**	**Retention time [min]**	**Calculated *m/z* [M+H]$^+$**	**Measured *m/z* [M+H]$^+$**	**Peak intensity m/z [M+H]$^+$ [counts]**	**LC/ESI-MS/MS *m/z* (% base peak)**
p-coumaroyl-1-phenylethanolate	$C_{17}H_{16}O_3$	11.9	269.1172	269.1146	$2.3 \cdot 10^3$	no MS2 event
p-coumaroyl-tryptamine	$C_{19}H_{18}O_2N_2$	10.0	307.1441	307.1357	$1.1 \cdot 10^3$	no MS2 event

Table S12 Summary of identified products formed in NbHCT9 assays and resulting LC/ESI-MS and LC/ESI-MS/MS data (negative (**A**) or positive (**B**) ionization mode), *m/z* calculated with Compass DataAnalysis, products with less than $1.0 \cdot 10^3$ counts are considered traces and not listed, only fragments >10 % base peak are listed.

**A**<br>**Detected product**	**Molecular formula**	**Retention time [min]**	**Calculated *m/z* [M-H]$^-$**	**Measured *m/z* [M-H]$^-$**	**Peak intensity m/z [M-H]$^-$ [counts]**	**LC/ESI-MS/MS *m/z* (% base peak)**
p-coumaroyl-2-*O*-homogentisic acid *or p*-coumaroyl-5-*O*-homogentisic acid	$C_{17}H_{14}O_6$	8.5	313.0707	313.1102	$7.0 \cdot 10^3$	MS2[313]: 227 (100)
**B**<br>**Detected product**	**Molecular formula**	**Retention time [min]**	**Calculated *m/z* [M+H]$^+$**	**Measured *m/z* [M+H]$^+$**	**Peak intensity m/z [M+H]$^+$ [counts]**	**LC/ESI-MS/MS *m/z* (% base peak)**
p-coumaroyl-1-phenylethanolate	$C_{17}H_{16}O_3$	11.9	269.1172	269.1122	$1.8 \cdot 10^3$	no MS2 event
p-coumaroyl-tryptamine	$C_{19}H_{18}O_2N_2$	10.0	307.1441	307.1481	$1.2 \cdot 10^3$	no MS2 event

Table S13 Sequences used in the phylogenetic tree (Figure S8). (**A**) sequences found in this study, (**B**) further selected sequences, (**C**) sequences taken from the phylogenetic analysis of Kruse *et al.* (2022).

**Abbreviation**	**Genbank accession or source**	**Species**
(**A**) **sequences found in this study**		
NbHCT1	PV344582	*Neoblechnum brasiliense*
NbHCT2	PV344583	*Neoblechnum brasiliense*
NbHCT3	PV344584	*Neoblechnum brasiliense*
NbHCT4	PV344585	*Neoblechnum brasiliense*
NbHCT5	PV344586	*Neoblechnum brasiliense*
NbHCT6	PV344587	*Neoblechnum brasiliense*
NbHCT7	PV344588	*Neoblechnum brasiliense*
NbHCT8	PV344589	*Neoblechnum brasiliense*

NbHCT9	PV344590	*Neoblechnum brasiliense*
(**B**) **further selected sequences**		
Ecol_CAT3	CAT3_ECOLX	*Escherichia coli*
Ecol_CAT2	CAT2_ECOLX	*Escherichia coli*
Ecol_CAT1	CAT_ECOLX	*Escherichia coli*
GhRAS1	Sander, 2010	*Glechoma hederacea*
GhRAS2	CDG56251.1	*Glechoma hederacea*
LaRAS	DQ886904	*Lavandula angustifolia*
MoRAS	G0LD36.1	*Melissa officinalis*
MlAT1	KAH6819558.1	*Mentha longifolia*
MlAT6	Zhou *et al.*, 2024	*Mentha longifolia*
OtRAS	QGN67259.1	*Ocimum tenuiflorum*
PfRAS	KAH6771934.1	*Perilla frutescens* var. *frutescens*
PcRAS	QDF44407.1	*Phacelia campanularia*
CbRAS	CAK55166	*Plectranthus scutellarioides*
SmRAS	ADA60182.1	*Salvia miltiorrhiza*
SgRAS	PP449351	*Sarcandra glabra*
PP449349_SarglaHST	PP449349	*Sarcandra glabra*
PP449350_SarglaHQT1	PP449350	*Sarcandra glabra*
PP449352_SarglaHQT2	PP449352	*Sarcandra glabra*
(**C**) **sequences taken from the phylogenetic analysis of Kruse *et al.* (2022)**		
ArHPT1_QAA12830.1	QAA12830.1	*Actaea racemosa*
AsFMT_JA758320.1	JA758320.1	*Angelica sinensis*
G10_Aagr_000038l.49.1	Kruse *et al.*, 2022	*Anthoceros agrestis*
At3AT1_NM_100275.3	NM_100275.3	*Arabidopsis thaliana*
At3AT2_NM_100232.5	NM_100232.5	*Arabidopsis thaliana*
AT3G47170_NP_190301.2	NP_190301.2	*Arabidopsis thaliana*
AT5G07080_OAO95042.1	OAO95042.1	*Arabidopsis thaliana*
At5Mat_NM_113880.2	NM_113880.2	*Arabidopsis thaliana*
AtACT_NM_125509.3	NM_125509.3	*Arabidopsis thaliana*
AtASFT_HHT1_AY062996.1	AY062996.1	*Arabidopsis thaliana*
AtBIA1_NP_193275.1_gen	NP_193275.1	*Arabidopsis thaliana*
AtCER2_AY087262_gen	AY087262	*Arabidopsis thaliana*
AtDCF_BT010448.1	BT010448.1	*Arabidopsis thaliana*
AtDCR_BT000770.1_gen	BT000770.1	*Arabidopsis thaliana*
AtEPS1_NM_126116.2	NM_126116.2	*Arabidopsis thaliana*

AtFACT_AY142537.1	AY142537.1	*Arabidopsis thaliana*
AtHCT_NM_124270.4	NM_124270.4	*Arabidopsis thaliana*
AtSCT_BT009686.1	BT009686.1	*Arabidopsis thaliana*
AtSDT_NM_127915.2	NM_127915.2	*Arabidopsis thaliana*
AtSHT_AT2G19070.1	AT2G19070.1	*Arabidopsis thaliana*
AtTHAA1_At5g47980	At5g47980	*Arabidopsis thaliana*
AtTHAA2_At5g47950	At5g47950	*Arabidopsis thaliana*
AsaHHT1_AB076980	AB076980	*Avena sativa*
BdPMT_HG421450.1_gen	HG421450.1	*Brachypodium distachyon*
CaPun1_NM_001324769_gen	NM_001324769	*Capsicum annuum*
CrDAT_AF053307	AF053307	*Catharanthus roseus*
CrMAT_AF253415	AF253415	*Catharanthus roseus*
CaAT20_MT277444	MT277444	*Celastrus angulatus*
Cbra_aGBG71897.1	GBG71897.1	*Chara braunii*
Cbra_bGBG71901.1	GBG71901.1	*Chara braunii*
Cbra_cGBG71902.1	GBG71902.1	*Chara braunii*
Cbra_dGBG76279.1	GBG76279.1	*Chara braunii*
G9_GBG76279.1_mod	GBG76279.1, Kruse *et al.*, 2022	*Chara braunii*
CiHCT1_KT222891.1	KT222891.1	*Cichorium intybus*
CiHCT2_KT222892.1	KT222892.1	*Cichorium intybus*
CiHQT1_KT222893.1	KT222893.1	*Cichorium intybus*
CiHQT2_KT222894.1	KT222894.1	*Cichorium intybus*
CiHQT3_KT222895.1	KT222895.1	*Cichorium intybus*
CiSHT1_MG457243.1	MG457243.1	*Cichorium intybus*
CiSHT2_MG457244.1	MG457244.1	*Cichorium intybus*
CbBEAT_AF043464	AF043464	*Clarkia breweri*
CbBEBT_AF500200	AF500200	*Clarkia breweri*
CcaHCT_EF137954.1	EF137954.1	*Coffea canephora*
CcaHQT_EF153931.1	EF153931.1	*Coffea canephora*
CcAT1_AXB26761.1	AXB26761.1	*Crocosmia x crocosmiiflora*
CcAT2_AXB26762.1	AXB26761.1	*Crocosmia x crocosmiiflora*
CmAAT1_CAA94432.1	CAA94432.1	*Cucumis melo*
CmAAT3_AY859053	AY859053	*Cucumis melo*
CmAAT4_AY859054	AY859054	*Cucumis melo*
CsHCT_JN005932.1_gen	JN005932.1	*Cucumis sativus*
CcHQT1_AM690438.2	AM690438.2	*Cynara cardunculus*

CcHQT2_EU839580.1	EU839580.1	*Cynara cardunculus*
Dp3MAT_AF489108	AF489108	*Dahlia pinnata*
Dm3MAT1_AY298809	AY298809	*Dendranthema x morifolium*
Dm3MAT2_AY298810	AY298810	*Dendranthema x morifolium*
Dm3MAT3_BAF50706.1	BAF50706.1	*Dendranthema x morifolium*
DcHCBT2_Z84386	Z84386	*Dianthus caryophyllus*
EpHCT_MT936805	MT936805	*Echinacea purpurea*
EpHHT_MT936803	MT936803	*Echinacea purpurea*
EpHQT_MT936804	MT936804	*Echinacea purpurea*
EcBAHD8_KC140150.1	KC140150.1	*Erythroxylum coca*
EcCS_KC140149.1	KC140149.1	*Erythroxylum coca*
EcHQT_JQ413187.1	JQ413187.1	*Erythroxylum coca*
FaAAT_AF193789	AF193789	*Fragaria ananassa*
FvVAAT_AX025504	AX025504	*Fragaria vesca*
Gt5AT_AB010708	AB010708	*Gentiana triflora*
GmIF7MaT_NM_001250831.1	NM_001250831.1	*Glycine max*
GmIMaT1_KY399789.1	KY399789.1	*Glycine max*
GmIMaT3_KY399790.1	KY399790.1	*Glycine max*
GmMT7_EU192928.1	EU192928.1	*Glycine max*
HmACT_LC596950	LC596950	*Hordeum murinum*
HvACT1-1_BAF97626	BAF97626	*Hordeum vulgare*
HvACT1-2_BAF97627	BAF97627	*Hordeum vulgare*
HvACT2_BAK00935	BAK00935	*Hordeum vulgare*
HvACT_AY228552	AY228552	*Hordeum vulgare*
Ih3AT1_AB217625.1	AB217625.1	*Iris hollandica*
Ih3AT2_AB242298.1	AB242298.1	*Iris hollandica*
Kni_kfl00070_0280_v1.1	GAQ81091.1	*Klebsormidium nitens*
Kni_kfl00302_0150_v1.1	GAQ86655.1	*Klebsormidium nitens*
Kni_kfl00513_0110_v1.1	GAQ89351.1	*Klebsormidium nitens*
Lp3MAT1_AY500352	AY500352	*Lamium purpureum*
LanAAT1_DQ886904.1	DQ886904.1	*Lavandula angustifolia*
LanAAT2_DQ886905.1	DQ886905.1	*Lavandula angustifolia*
LeAAT1_LC520138	LC520138	*Lithospermum erythrorhizon*
LeSAT1_LC520137	LC520137	*Lithospermum erythrorhizon*
LaHMT_HLT_AB181292	HLT_AB181292	*Lupinus albus*
MdAAT1_AY707098	AY707098	*Malus domestica*
MdAAT2_AY517491.1_gen	AY517491.1	*Malus domestica*

MeHFT_MeHFT	MeHFT	*Marchantia emarginata*
G1_Mpol_Mapoly0005s0261.1.p	Mapoly0005s0261	*Marchantia polymorpha*
G8_MpolHCT_Mapoly0003s0277.1.p	Mapoly0003s0277, Kruse *et al.*, 2022	*Marchantia polymorpha*
MpolFHT_Mapoly0157s0023.1.p	Mapoly0003s0277	*Marchantia polymorpha*
MpolHCT_Mapoly0003s0277.1.p	Mapoly0003s0277	*Marchantia polymorpha*
MtMaT1_EU272030.1	EU272030.1	*Medicago truncatula*
MtMaT2_EU272032.1	EU272032.1	*Medicago truncatula*
MtMaT3_EU272031.1	EU272031.1	*Medicago truncatula*
MoRAS_FR670523.1	FR670523.1	*Melissa officinales*
Mpus_123940	Kruse *et al.*, 2022	*Micromonas pusilla*
MsAAT_AX025506	AX025506	*Musa sapientum*
NaAT1_JN390826.1	JN390826.1	*Nicotiana attenuata*
NaCV86_JN390825.1_gen	JN390825.1	*Nicotiana attenuata*
NaDH29_JN390824.1	JN390824.1	*Nicotiana attenuata*
NtBEBT_AF500202	AF500202	*Nicotiana tabacum*
NtHCT_AJ507825	AJ507825	*Nicotiana tabacum*
NtHQT_AJ582651	AJ582651	*Nicotiana tabacum*
NtMAT1_AB176525	AB176525	*Nicotiana tabacum*
NtSHT_MN787045	MN787045	*Nicotiana tabacum*
ObCAAT1_MN031888	MN031888	*Ocimum basilicum*
ObCAAT2_MN031889	MN031889	*Ocimum basilicum*
Os09g0544000_XP_015651357	XP_015651357	*Oryza sativa*
OsAHT1_ANQ47369	ANQ47369	*Oryza sativa*
OsHCT_XM_015786263.1	XM_015786263.1	*Oryza sativa*
OsPHT3_Os09g37180.1	Os09g37180.1	*Oryza sativa*
OsPHT4_Os09g37200	Os09g37200	*Oryza sativa*
OsPMT_XM_015765814.1	XM_015765814.1	*Oryza sativa*
OsSAT1_gen	Q2QRK9.1	*Oryza sativa*
OsSAT2_XM_015763244.1_gen	XM_015763244.1	*Oryza sativa*
OsTBT1_Os11g42290	Os11g42290	*Oryza sativa*
OsTBT2_Os11g42370	Os11g42370	*Oryza sativa*
OsTHT1_AK287765.1	AK287765.1	*Oryza sativa*
OsTHT2_XM_015757482.1	XM_015757482.1	*Oryza sativa*
Oluc_28702	Kruse *et al.*, 2022	*Ostreococcus lucimarinus*
PvHCT1a_AB723827.1	AB723827.1	*Panicum virgatum*
PvHCT2a_KC696573.1	KC696573.1	*Panicum virgatum*

PvHCTLike1_AFY17066.1	AFY17066.1	*Panicum virgatum*
PsSALAT_AF339913	AF339913	*Papaver somniferum*
Pc3MAT_AY190121	AY190121	*Pericallis cruenta*
Pf3AT_AB029340	AB029340	*Perilla frutescens*
PaxASAT1_KT716258.1	KT716258.1	*Petunia axillaris*
PaxASAT2_KT716259.1	KT716259.1	*Petunia axillaris*
PaxASAT3_KT716260.1	KT716260.1	*Petunia axillaris*
PaxASAT4_KT716261.1	KT716261.1	*Petunia axillaris*
PaxCFAT_DQ767969.1	DQ767969.1	*Petunia axillaris*
PhBPBT_AY611496	AY611496	*Petunia x hybrida*
PvHHHT_KX443573.1	KX443573.1	*Phaseolus vulgaris*
G2_Ppat_Pp3c3_250V3.1.p	Kruse *et al.*, 2022	*Physcomitrium patens*
G3_Ppat_Pp3c14_6190V3.1.p	Kruse *et al.*, 2022	*Physcomitrium patens*
PpHCT1_KU513965.1	KU513965.1	*Physcomitrium patens*
PrHCT_EF121452.1	EF121452.1	*Pinus radiata*
PnPAS_MW354957	MW354956	*Piper nigrum*
PnPS_MW354956	MW354957	*Piper nigrum*
PsHCT2_FN647681.1	FN647681.1	*Plectranthus scutellarioides*
PsRAS_AM283092.1	AM283092.1	*Plectranthus scutellarioides*
PtHCT6_XM_006368430.2	XM_006368430.2	*Populus tremuloides*
PtBEBT_KP228019.1	KP228019.1	*Populus trichocarpa*
PtFHT1_JX515962.1	JX515962.1	*Populus trichocarpa*
PtHCT1_EU603313.1	EU603313.1	*Populus trichocarpa*
PtSABT_KP228018.1	KP228018.1	*Populus trichocarpa*
PpAAT1_MH700174	MH700174	*Prunus persica*
RsACT_AJ556780	AJ556780	*Rauvolfia serpentina*
RicSHT_MN787043	MN787043	*Ricinus communis*
RocSHT_MN787046	MN787046	*Rosa canina*
RhAAT1_AY850287	AY850287	*Rosa hybrida*
SsiASAT1_KY978746.1	KY978746.1	*Salpiglossis sinuata*
SsiASAT2_KY978747.1	KY978747.1	*Salpiglossis sinuata*
SsiASAT3_KY978748.1	KY978748.1	*Salpiglossis sinuata*
SsiASAT5_KY978749.1	KY978749.1	*Salpiglossis sinuata*
Ss5MAT1_AF405707	AF405707	*Salvia splendens*
Ss5MAT2_AY383734	AY383734	*Salvia splendens*
SmHCT1a_XM_002979015.2	XM_002979015.2	*Selaginella moellendorffii*
ShASAT2_KT359561.1	KT359561.1	*Solanum habrochaites*

SlAAT1_KM975322.1	KM975322.1	*Solanum lycopersicum*
SlACT1_Solyc11g071470.1	Solyc11g071470	*Solanum lycopersicum*
SlACT2_Solyc11g071480.1.1	Solyc11g071480	*Solanum lycopersicum*
SlASAT1_NM_001329403.1	NM_001329403.1	*Solanum lycopersicum*
SlASAT2_NM_001329392.1	NM_001329392.1	*Solanum lycopersicum*
SlASAT3	Solyc11g067270	*Solanum lycopersicum*
SlASAT4	Solyc01g105580	*Solanum lycopersicum*
SlHCT_Solyc03g117600.2.1	Solyc03g117600	*Solanum lycopersicum*
SlHQT_AJ582652.1	AJ582652.1	*Solanum lycopersicum*
SlHQT_Solyc07g005760.2.1	Solyc07g005760	*Solanum lycopersicum*
SlSHT_MN787044	MN787044	*Solanum lycopersicum*
SmSpmHT_AKP24035.1	AKP24035.1	*Solanum melongena*
SpAAT1_KM975321.1	KM975321.1	*Solanum pennellii*
SqTAITAT_MT024677	MT024677	*Solanum quitoense*
SrSpmHT_AMB21755.1	AMB21755.1	*Solanum richardii*
StFHT_NM_001288261.1	NM_001288261.1	*Solanum tuberosum*
SbHCT_XM_002452390.2	XM_002452390.2	*Sorghum bicolor*
G4_Sfal_Sphfalx02G197500.1.p	Sphfalx02G197500, Kruse *et al.*, 2022	*Sphagnum fallax*
G5_Sfal_Sphfalx08G078400.1.p	Sphfalx08G078400, Kruse *et al.*, 2022	*Sphagnum fallax*
G6_Sfal_Sphfalx09G052100.1.p	Sphfalx09G052100, Kruse *et al.*, 2022	*Sphagnum fallax*
G7_Sfal_Sphfalx12G001300.1.p	Sphfalx12G001300, Kruse *et al.*, 2022	*Sphagnum fallax*
SoSOAP10_KNA12459.1	KNA12459.1	*Spinacia oleracea*
TcBAPT_AY082804	AY082804	*Taxus cuspidata*
TcDBAT_AF193765	AF193765	*Taxus cuspidata*
TcDBBT_AF297618	AF297618	*Taxus cuspidata*
TcDBNTBT_AF466397	AF466397	*Taxus cuspidata*
TcTAT_AF190130	AF190130	*Taxus cuspidata*
TfHCT1A_EU861218.1	EU861218.1	*Trifolium pratense*
TfHCT1B_FJ151489.1	FJ151489.1	*Trifolium pratense*
TfHCT2_EU861219.1	EU861219.1	*Trifolium pratense*
TaACT1-1_LC596951	LC596951	*Triticum aestivum*
TaACT1-2_LC596952	LC596952	*Triticum aestivum*
Vh3MAT1_AY500350	AY500350	*Verbena hybrida*
VlAMAT_AY705388	AY705388	*Vitis labrusca*

Vv3AT_KM267559.1	KM267559.1	*Vitis vinifera*
VvSHT_MN787047	MN787047	*Vitis vinifera*
ZmAat1_NP_001148286.2	NP_001148286.2	*Zea mays*
ZmGlossy2_Q41809	Q41809	*Zea mays*
ZmpCAT_BT042717.1	BT042717.1	*Zea mays*

Table S14 PCR conditions and primers for amplification of *NbHCT1-9*; restriction sites are underlined; fw - forward, rv – reverse, UPMA – 10x Universal primer Mix A, UPS – 10 µM Universal Primer short.

Sequence	Primer (5’ -> 3’)	Composition	PCR
NbHCT1	Full-length PCR<br>fw: ATCTCGAGATGAAGGTGAAAATGAGATGTGCTAAG<br>rv: ATCTCGAGCTAGAATTGATAGAAAAGGTGTCTGAAG	1 µL 5’-RACE cDNA, 5 µL 5x GoTaq® buffer, 0.5 µL 10 mM dNTPs, 3 µL 25 mM $MgCl_2$, 0.5 µL of each primer (10 µM), 0.1 µL GoTaq® polymerase, 14.4 µL H_2O	94 °C 90 s<br>[94 °C 30 s, 58/61/63 °C 60 s, 72 °C 90 s] x39<br>94 °C 60 s, 58/61/63 °C 60 s, 72 °C 600s
NbHCT2	5’-RACE PCR<br>fw: UPMA<br>rv: CTTCTAGGGAGTCCCCATTTAGGGAGTCCCCATTTTG	7.75 µL H_2O, 12.5 µL 2x SeqAmp™ buffer, 0.5 µL SeqAmp DNA Polymerase, 1.25 µL 5’-RACE cDNA, 2.5 µL UPMA, 0.5 µL 100 µM 5’-RACE rv-primer	[94 °C 30 s, 72 °C 120 s] x5<br>[94 °C 30 s, 70 °C 30 s, 72 °C 120 s] x5<br>[94 °C 30 s, 69 °C 30 s, 72 °C 120 s] x40
	5’-RACE PCRnested<br>fw: UPS<br>rv: CTTCTAGGGAGTCCCCATTTAGGGAGTCCCCATTTTG	1 µL 1:50-diluted *NbHCT2* 5’-RACE-PCR NucleoSpin clean-up, 5 µL 5x GoTaq® buffer, 0.5 µL 10 mM dNTPs, 3 µL 25 mM $MgCl_2$, 0.5 µL 100 µM 5’-RACE rv-primer, 5 µL UPS, 0.1 µL GoTaq® DNA Polymerase (5 U/µL), 9.9 µL H_2O	[98 °C 60 s, 72 °C (-1 °C/repeat) 60 s, 72 °C 120 s] x4<br>[98 °C 60 s, 68 °C 60 s, 72 °C 120 s] x40<br>72 °C 180 s
	3’-RACE PCR<br>fw: CCAACTCCCTCCTGCGTTGCTGCATGAG<br>rv: UPMA	7.75 µL H_2O, 12.5 µL 2x SeqAmp™ buffer, 0.5 µL SeqAmp DNA Polymerase, 1.25 µL 3’-RACE cDNA, 2.5 µL UPMA, 0.5 µL 100 µM 3’-RACE fw-primer	[94 °C 30 s, 72 °C 120 s] x5<br>[94 °C 30 s, 70 °C 30 s, 72 °C 120 s] x5<br>[94 °C 30 s, 69 °C 30 s, 72 °C 120 s] x40

	3'-RACE PCRnested<br>fw: CCAACTCCCTCCTGCGTTGCTGCATGAG<br>rv: UPS	1 µL 1:50-diluted *NbHCT2* 3'-RACE-PCR NucleoSpin clean-up, 5 µL 5x GoTaq® buffer, 0.5 µL 10 mM dNTPs, 3 µL 25 mM $MgCl_2$, 0.5 µL 100 µM 3'-RACE fw-primer, 2.5 µL UPS, 0.1 µL GoTaq® DNA Polymerase (5 U/µL), 14.4 µL H_2O	[98 °C 60 s, 72 °C (-1 °C/repeat) 60 s, 72 °C 120 s] x4<br>[98 °C 60 s, 68 °C 60 s, 72 °C 120 s] x40<br>72 °C 180 s
	Full-length PCR<br>fw: GCCGCGCGGCAGCCATATGAAGGTGTTGATACGGCGATCAAAATTGGTTACG<br>rv: TAGCAGCCGGATCCTCGAGTCAAAACTTGAAGAAGAGTCGTTCAAAGGCAGGC	1 µL 5'-RACE cDNA, 2.5 µL 10x AccuPrime™ PCR buffer I, 0.5 µL of each primer (10 µM), 0.1 µL AccuPrime™ Taq DNA Polymerase High Fidelity (5 U/µL), 20.4 µL H_2O	[95 °C 60 s, 76 °C (-1 °C/repeat) 60 s, 68 °C 120 s] x8<br>[95 °C 60 s, 68 °C 180 s] x40<br>68 °C 180 s
NbHCT3	5'-RACE PCR<br>fw: UPMA<br>rv: ACATGGGTGGCATTSCCCCAYCCAAARTC	7.75 µL H_2O, 12.5 µL 2x SeqAmp™ buffer, 0.5 µL SeqAmp DNA Polymerase, 1.25 µL 5'-RACE cDNA, 2.5 µL UPMA, 0.5 µL 100 µM 5'-RACE rv-primer	[94 °C 30 s, 72 °C 120 s] x5<br>[94 °C 30 s, 70 °C 30 s, 72 °C 120 s] x5<br>[94 °C 30 s, 68 °C 30 s, 72 °C 120 s] x29
	5'-RACE PCRnested<br>fw: UPS<br>rv: ACATGGGTGGCATTSCCCCAYCCAAARTC	7.75 µL H_2O, 12.5 µL 2x SeqAmp™ Buffer, 0.5 µL SeqAmp DNA Polymerase, 2.5 µL 1:50-diluted *NbHCT3* 5'-RACE-PCR NucleoSpin clean-up, 0.5 µL UPS, 0.5 µL 10 µM 5'-RACE rv-primer	[94 °C 30 s, 68 °C 30 s, 72 °C 120 s] x29
	3'-RACE PCR<br>fw: CCTTCTACGAGATCGAYTTTGGRTGGGGS<br>rv: UPMA	7.25 µL H_2O, 12.5 µL 2x SeqAmp™ buffer, 0.5 µL SeqAmp DNA Polymerase, 1.25 µL 3'-RACE cDNA, 2.5 µL UPMA, 1 µL 100 µM 3'-RACE fw-primer	[94 °C 30 s, 72 °C 120 s] x5<br>[94 °C 30 s, 70 °C 30 s, 72 °C 120 s] x5<br>[94 °C 30 s, 69 °C 30 s, 72 °C 120 s] x40
	Full-length PCR<br>fw: CGACATATGGTTTTGTCTGTAACTCCTTGCG<br>rv: GACTCGAGTTATGAAGGTAGAAGGATTTGGGAAAATTTG	1 µL 5'-RACE cDNA, 2.5 µL 10x AccuPrime™ PCR buffer I, 0.5 µL of each primer (10 µM), 0.1 µL AccuPrime™ Taq DNA Polymerase High Fidelity (5 U/µL), 20.4 µL H_2O	95 °C 90 s<br>[95 °C 30 s, 70 °C 60 s, 68 °C 90 s] x40<br>68 °C 210 s
NbHCT4	5'-RACE PCR<br>fw: UPMA<br>rv: CTACATGACCTGCCCCCATTGGAGAGGG	7.75 µL H_2O, 12.5 µL 2x SeqAmp™ Buffer, 0.5 µL SeqAmp DNA Polymerase, 1.25 µL 5'-RACE cDNA, 2.5 µL 10x UPMA, 0.5 µL 10 µM 5'-RACE rv-primer	[94 °C 30 s, 72 °C 120 s] x5<br>[94 °C 30 s, 70 °C 30 s, 72 °C 120 s] x5<br>[94 °C 30 s, 68 °C 30 s, 72 °C 120 s] x30

	3'-RACE PCR<br>fw: GACGCCCTAAGGGCAAAGGCAAGCCTC<br>rv: UPMA	7.75 µL H_2O, 12.5 µL 2x SeqAmp™ Buffer, 0.5 µL SeqAmp DNA Polymerase, 1.25 µL 3'-RACE cDNA, 2.5 µL UPMA, 0.5 µL 100 µM 3'-RACE fw-primer	[94 °C 30 s, 72 °C 120 s] x5<br>[94 °C 30 s, 70 °C 30 s, 72 °C 120 s] x5<br>[94 °C 30 s, 69 °C 30 s, 72 °C 120 s] x40
	3'-RACE PCRnested<br>fw: GACGCCCTAAGGGCAAAGGCAAGCCTC<br>rv: UPS	1 µL 1:50-diluted *NbHCT4* 3'-RACE-PCR NucleoSpin clean-up, 5 µL 5x GoTaq® buffer, 0.5 µL 10 mM dNTPs, 3 µL 25 mM $MgCl_2$, 0.5 µL 100 µM 3'-RACE fw-primer, 5 µL UPS, 0.1 µL GoTaq® DNA Polymerase (5 U/µL), 9.9 µL H_2O	[98 °C 60 s, 72 °C (-1 °C/repeat) 60 s, 72 °C 120 s] x4<br>[98 °C 60 s, 68 °C 60 s, 72 °C 120 s] x40<br>72 °C 180 s
	Full-length PCR<br>fw: GCCGCGCGGCAGCCATATGCAGGTGAAGAAGACGACCCAAGAGAC<br>rv: TAGCAGCCGGATCCTCGAGTTACAAATCTGCAATGTATTCTTCAAAATTGCGCATGTGCTC	1 µL 5'-RACE cDNA, 2.5 µL 10x AccuPrime™ PCR buffer I, 0.5 µL of each primer (10 µM), 0.1 µL AccuPrime™ Taq DNA Polymerase High Fidelity (5 U/µL), 20.4 µL H_2O	[95 °C 60 s, 76 °C (-1 °C/repeat) 60 s, 68 °C 120 s] x8<br>[95 °C 60 s, 68 °C 180 s] x40<br>68 °C 180 s
NbHCT5	5'-RACE PCR<br>fw: UPMA<br>rv: CTACATGACCTGCCCCCATTGGAGAGGG	7.75 µL H_2O, 12.5 µL 2x SeqAmp™ buffer, 0.5 µL SeqAmp DNA Polymerase, 1.25 µL 5'-RACE cDNA, 2.5 µL UPMA, 0.5 µL 10 µM 5'-RACE rv-primer	[94 °C 30 s, 72 °C 120 s] x5<br>[94 °C 30 s, 70 °C 30 s, 72 °C 120 s] x5<br>[94 °C 30 s, 68 °C 30 s, 72 °C 120 s] x30
	3'-RACE PCR<br>fw: GACGCCCTAAGGGCAAAGGCAAGCCTC<br>rv: UPMA	7.75 µL H_2O, 12.5 µL 2x SeqAmp™ buffer, 0.5 µL SeqAmp DNA Polymerase, 1.25 µL 3'-RACE cDNA, 2.5 µL UPMA, 0.5 µL 100 µM 3'-RACE fw-primer	[94 °C 30 s, 72 °C 120 s] x5<br>[94 °C 30 s, 70 °C 30 s, 72 °C 120 s] x5<br>[94 °C 30 s, 69 °C 30 s, 72 °C 120 s] x40
	3'-RACE PCRnested<br>fw: GACGCCCTAAGGGCAAAGGCAAGCCTC<br>rv: UPS	1 µL 1:50-diluted *NbHCT5* 3'-RACE-PCR NucleoSpin clean-up, 5 µL 5x GoTaq® buffer, 0.5 µL 10 mM dNTPs, 3 µL 25 mM $MgCl_2$, 0.5 µL100 µM 3'-RACE fw-primer, 5 µL UPS, 0.1 µL GoTaq® DNA Polymerase (5 U/µL), 9.9 µL H_2O	[98 °C 60 s, 72 °C (-1 °C/repeat) 60 s, 72 °C 120 s] x4<br>[98 °C 60 s, 68 °C 60 s, 72 °C 120 s] x40<br>72 °C 180 s

	Full-length PCR<br>fw: GCCGCGCGGCAGCCATATGCAGGTGAAGAAGACGACCCAAGAGAC<br>rv: TAGCAGCCGGATCCTCGAGTTACAAATCTGCAATGTATTCTTCAAAATTGCGCATGTGCTC	1 µL 5’-RACE cDNA, 2.5 µL 10x AccuPrime™ PCR buffer I, 0.5 µL of each primer (10 µM), 0.1 µL AccuPrime™ Taq DNA Polymerase High Fidelity (5 U/µL), 20.4 µL H_2O	[95 °C 60 s, 76 °C (-1 °C/repeat) 60 s, 68 °C 120 s] x8<br>[95 °C 60 s, 68 °C 180 s] x40<br>68 °C 180 s
NbHCT6	5’-RACE PCR<br>fw. UPMA<br>rv: GAAGATGGGGCGSCCCCAYCCAAARTC	7.75 µL H_2O, 12.5 µL 2x SeqAmp™ buffer, 0.5 µL SeqAmp DNA Polymerase, 1.25 µL 5’-RACE cDNA, 2.5 µL UPMA, 0.5 µL 10 µM 5’-RACE primer	[94 °C 30 s, 72 °C 120 s] x5<br>[94 °C 30 s, 70 °C 30 s, 72 °C 120 s] x5<br>[94 °C 30 s, 68 °C 30 s, 72 °C 120 s] x29
	3’-RACE PCR<br>fw: ACAGAGAAGGATCGGGAGCTTCAGCCAGCTT<br>rv: UPMA	7.75 µL H_2O, 12.5 µL 2x SeqAmp™ buffer, 0.5 µL SeqAmp DNA Polymerase, 1.25 µL 3’-RACE cDNA, 2.5 µL UPMA, 0.5 µL 10 µM 3’-RACE primer	[94 °C 30 s, 72 °C 120 s] x5<br>[94 °C 30 s, 70 °C 30 s, 72 °C 120 s] x5<br>[94 °C 30 s, 69 °C 30 s, 72 °C 120 s] x40
	Full-length PCR<br>fw: GAGGATCCAATGGTCAATATGAAGCAAACAGAGAAGG<br>rv: GAGGATCCTCACGAAGAAGTGGAGCTGCTAATG	1 µL 5’-RACE cDNA, 2.5 µL 10x AccuPrime™ PCR buffer I, 0.5 µL of each primer (10 µM), 0.1 µL AccuPrime™ Taq DNA Polymerase High Fidelity (5 U/µL), 20.4 µL H_2O	[95 °C 60 s, 65 °C (-1 °C/repeat) 60 s, 68 °C 90 s] x8<br>[95 °C 60 s, 58 °C 60 s, 68 °C 90 s] x30<br>68 °C 150 s
	Full-length PCRnested<br>fw: GCCATATGCTCGAGGATCCAATGGTCAATATGAAGCAAACAGAGAAGG<br>rv: CTTTGTTAGCAGCCGGATCCTCACGAAGAAGTGGAGCTGCTAA	1 µL 1:50-diluted *NbHCT6* full-length-PCR NucleoSpin clean-up, 2.5 µL 10x AccuPrime™ PCR buffer I, 0.5 µL of each primer (10 µM), 0.1 µL AccuPrime™ Taq DNA Polymerase High Fidelity (5 U/µL), 20.4 µL H_2O	[95 °C 60 s, 65 °C (-1 °C/repeat) 60 s, 68 °C 90 s] x8<br>[95 °C 60 s, 58 °C 60 s, 68 °C 90 s] x30<br>68 °C 150 s
NbHCT7	5’-RACE PCR<br>fw: UPMA<br>rv: GAAGATGGGGCGSCCCCAYCCAAARTC	7.75 µL H_2O, 12.5 µL 2x SeqAmp™ buffer, 0.5 µL SeqAmp DNA Polymerase, 1.25 µL 5’-RACE cDNA, 2.5 µL UPMA, 0.5 µL 10 µM 5’-RACE primer	[94 °C 30 s, 72 °C 120 s] x5<br>[94 °C 30 s, 70 °C 30 s, 72 °C 120 s] x5<br>[94 °C 30 s, 68 °C 30 s, 72 °C 120 s] x29
	3’-RACE PCR<br>fw: CCTCATGAGATTAAGGATCAGGAGCTTCAGCCAG<br>rv: UPMA	7.75 µL H_2O, 12.5 µL 2x SeqAmp™ buffer, 0.5 µL SeqAmp DNA Polymerase, 1.25 µL 3’-RACE cDNA, 2.5 µL UPMA, 0.5 µL 10 µM 3’-RACE primer	[94 °C 30 s, 72 °C 120 s] x5<br>[94 °C 30 s, 70 °C 30 s, 72 °C 120 s] x5<br>[94 °C 30 s, 69 °C 30 s, 72 °C 120 s] x40

	Full-length PCR<br>fw: GAGGATCCAATGGTTAATATGAAGCAACCTCATGAGATTAA<br>rv: GAGGATCCTCATGTAATATACTCTGCAATTTCTTCTTGGAA	1 µL 5'-RACE cDNA, 2.5 µL 10x AccuPrime™ PCR buffer I, 0.5 µL of each primer (10 µM), 0.1 µL AccuPrime™ Taq DNA Polymerase High Fidelity (5 U/µL), 20.4 µL H_2O	[95 °C 60 s, 65 °C (-1 °C/repeat) 60 s, 68 °C 90 s] x8<br>[95 °C 60 s, 58 °C 60 s, 68 °C 90 s] x30<br>68 °C 150 s
NbHCT8	5'-RACE PCR<br>fw: UPMA<br>rv: GGGTTCCTGTCTTCTGATATAATCTCCATTGTAGACACCAT	7.75 µL H_2O, 12.5 µL 2x SeqAmp™ buffer, 0.5 µL SeqAmp DNA Polymerase, 1.25 µL 5'-RACE cDNA, 2.5 µL UPMA, 0.5 µL 100 µM 5'-RACE Primer	[94 °C 30 s, 72 °C 120 s] x5<br>[94 °C 30 s, 70 °C 30 s, 72 °C 120 s] x5<br>[94 °C 30 s, 68 °C 30 s, 72 °C 120 s] x29
	5'-RACE PCRnested<br>fw: UPS<br>rv: GGGTTCCTGTCTTCTGATATAATCTCCATTGTAGACACCAT	1 µL 1:50-diluted *NbHCT8* 5'-RACE-PCR NucleoSpin clean-up, 5 µL 5x GoTaq® buffer, 0.5 µL 10 mM dNTPs, 3 µL 25 mM $MgCl_2$, 0.5 µL10 µM 5'-RACE rv-primer, 2.5 µL UPS, 0.1 µL GoTaq® DNA Polymerase (5 U/µL), 13.2 µL H_2O	[98 °C 60 s, 72 °C (-1 °C/repeat) 60 s, 72 °C 120 s] x4<br>[98 °C 60 s, 68 °C 60 s, 72 °C 120 s] x40<br>72 °C 180 s
	3'-RACE PCR<br>fw: GGCACTGGGGAGGGCTTTAGTGCCCTTC<br>rv: UPMA	7.75 µL H_2O, 12.5 µL 2x SeqAmp™ buffer, 0.5 µL SeqAmp DNA Polymerase, 1.25 µL 3'-RACE cDNA, 2.5 µL UPMA, 0.5 µL 100 µM 3'-RACE primer	[94 °C 30 s, 72 °C 120 s] x5<br>[94 °C 30 s, 70 °C 30 s, 72 °C 120 s] x5<br>[94 °C 30 s, 68 °C 30 s, 72 °C 120 s] x29
	3'-RACE PCRnested<br>fw: GGCACTGGGGAGGGCTTTAGTGCCCTTC<br>rv: UPS	1 µL 1:50-diluted *NbHCT8* 3'-RACE-PCR NucleoSpin clean-up, 5 µL 5x GoTaq® buffer, 0.5 µL 10 mM dNTPs, 3 µL 25 mM $MgCl_2$, 0.5 µL10 µM 3'-RACE rv-primer, 2.5 µL UPS, 0.1 µL GoTaq® DNA Polymerase (5 U/µL), 13.2 µL H_2O	[98 °C 60 s, 72 °C (-1 °C/repeat) 60 s, 72 °C 120 s] x4<br>[98 °C 60 s, 68 °C 60 s, 72 °C 120 s] x40<br>72 °C 180 s
	Full-length PCR<br>fw: GCCGCGCGGCAGCCATATGAAGATCACCATTAAGAGCTCCACTATAGTGAAG<br>rv: TAGCAGCCGGATCCTCGAGTCAGAATTGGTAGATGAGCTCCCGGAAG	1 µL 5'-RACE cDNA, 2.5 µL 10x AccuPrime™ PCR Buffer I, 0.5 µL of each primer (10 µM), 0.1 µL AccuPrime™ Taq DNA Polymerase High Fidelity (5 U/µL), 20.4 µL H_2O	[95 °C 60 s, 76 °C (-1 °C/repeat) 60 s, 68 °C 120 s] x8<br>[95 °C 60 s, 68 °C 180 s] x40<br>68 °C 180 s
NbHCT9	5'-RACE PCR<br>fw: UPMA<br>rv: GACCCCTTGCTATGCTAGCGTGTTTCCATATGTGGGC	7.75 µL H_2O, 12.5 µL 2x SeqAmp™ buffer, 0.5 µL SeqAmp DNA Polymerase, 1.25 µL 5'-RACE cDNA, 2.5 µL UPMA, 0.5 µL 100 µM 5'-RACE primer	[94 °C 30 s, 72 °C 120 s] x5<br>[94 °C 30 s, 70 °C 30 s, 72 °C 120 s] x5<br>[94 °C 30 s, 68 °C 30 s, 72 °C 120 s] x29

	3'-RACE PCR<br>fw: CTGCGGTGGCCACCTCAGGAGGAGG<br>rv: UPMA	7.75 µL H_2O, 12.5 µL 2x SeqAmp™ buffer, 0.5 µL SeqAmp DNA Polymerase, 1.25 µL 3'-RACE cDNA, 2.5 µL UPMA, 0.5 µL 10 µM 3'-RACE primer	[94 °C 30 s, 72 °C 120 s] x5<br>[94 °C 30 s, 70 °C 30 s, 72 °C 120 s] x5<br>[94 °C 30 s, 68 °C 30 s, 72 °C 120 s] x29
	Full-length PCR<br>fw: GCCGCGCGGCAGCCATATGGCAGCAGTAAGGATTGTAGAGAGGCG<br>rv: TAGCAGCCGGATCCTCGAGCTAAGGGTTAATAAAAGCATGGTCATTTGCAAGCC	1 µL 3'-RACE cDNA, 2.5 µL 10x AccuPrime™ PCR buffer I, 0.5 µL of each primer (10 µM), 0.1 µL AccuPrime™ Taq DNA Polymerase High Fidelity (5 U/µL), 20.4 µL H_2O	[95 °C 60 s, 76 °C (-1 °C/repeat) 60 s, 68 °C 120 s] x8<br>[95 °C 60 s, 68 °C 180 s] x40<br>68 °C 180 s

Table S15 Conditions, composition and detection parameters for the determination of pH and temperature optima of NbHCT1, NbHCT3 and NbHCT8.

Parameter	Condition	Composition	Detection parameter
**NbHCT1**			
pH-optimum	ϑ = 30 °C<br>t = 15 min,<br>negative control:<br>t = 0 min *n* = 3	400 mM KP_i-buffer, pH 6.13-8.13<br>100 µM caffeoyl-CoA<br>800 µM shikimic acid<br>2.8 µg NbHCT1	injection volume: 20 µL<br>UV-detector: λ = 333 nm<br>run time: t = 30 min<br>mobile phase: 35 % methanol, 0.01 % *o*-phosphoric acid<br>flow rate: 1 mL/min<br>stationary phase: Equisil ODS C18 5 µm, 250 x 4 mm
temperature optimum	ϑ = 1.3-43.8 °C<br>t = 10 min,<br>negative control:<br>t = 0 min *n* = 5	88 mM KP_i-buffer, pH 7.0<br>100 µM caffeoyl-CoA<br>80 µM shikimic acid<br>7.0 µg NbHCT1	
**NbHCT3**			
pH-optimum	ϑ = 30 °C<br>t = 20 min,<br>negative control:<br>t = 0 min *n* = 3	400 mM KP_i-buffer, pH 6.09-8.22<br>100 µM *p*-coumaroyl-CoA<br>80 mM quinic acid<br>0.15 µg NbHCT3	injection volume: 20 µL<br>DAD-detector: λ = 312 nm<br>run time: t = 30 min<br>mobile phase: 20 % methanol, 0.01 % *o*-phosphoric acid<br>flow rate: 1 mL/min<br>stationary phase: Hypersil ODS C18 5 µm, 250 x 4 mm<br>column temperature: ϑ = 35 °C
temperature optimum	ϑ = 0.1-50.2 °C<br>t = 20 min,<br>negative control:<br>t = 0 min *n* = 3	88 mM KP_i-buffer, pH 7.0<br>100 µM *p*-coumaroyl-CoA<br>80 mM quinic acid<br>0.15 µg NbHCT3	
**NbHCT8**			
pH-optimum	ϑ = 40 °C<br>t = 30 min,<br>negative control:<br>t = 0 min *n* = 3	400 mM KP_i-buffer, pH 6.08-8.22<br>58 µM *p*-coumaroyl-CoA<br>4 mM shikimic acid<br>0.1 µg NbHCT8	injection volume: 50 µL<br>DAD-detector: λ = 312 nm<br>run time: t = 15 min<br>mobile phase: 35 % methanol, 0.01 % *o*-phosphoric acid

temperature optimum	ϑ = 1.1-45.4 °C<br>t = 30 min,<br>negative control:<br>t = 0 min *n* = 3	88 mM KP_i-buffer, pH 7.0<br>58 µM *p*-coumaroyl-CoA<br>8 mM shikimic acid<br>0.25 µg NbHCT8	flow rate: 1 mL/min<br>stationary phase: Hypersil ODS C18 5 µm, 250 x 4 mm<br>column temperature: ϑ = 35 °C

Table S16 Conditions, composition and detection parameters for the determination of kinetic parameters for NbHCT1, NbHCT3 and NbHCT8.

**K_m for**	**with** [corresponds to x times K_m-value]	**Condition**	**Composition**	**Detection parameters**
**NbHCT1**				
caffeoyl-CoA	shikimic acid [8.8]	ϑ = 30 °C<br>t = 15 min,<br>negative control:<br>t = 0 min<br>*n* = 3 x 3	360 mM KP_i, pH 7.0<br>1-200 µM caffeoyl-CoA<br>10 mM shikimic acid<br>2.0 µg NbHCT1	injection volume: 50 µL<br>DAD-detector: λ = 333 nm<br>run time: t = 15 min<br>mobile phase: 35 % methanol, 0.01 % *o*-phosphoric acid<br>flow rate: 1 mL/min<br>stationary phase: Hypersil ODS C18 5 µm, 250 x 4 mm<br>column temperature: ϑ = 35 °C
shikimic acid	caffeoyl-CoA [7.2]	ϑ = 30 °C<br>t = 5 min,<br>negative control:<br>t = 0 min<br>*n* = 3 x 3	360 mM KP_i, pH 7.0<br>200 µM caffeoyl-CoA<br>0.5-16 mM shikimic acid<br>2.0 µg NbHCT1	
p-cou-maroyl-CoA	shikimic acid [6.4]	ϑ = 30 °C<br>t = 20 min,<br>negative control:<br>t = 0 min<br>*n* = 3 x 3	360 mM KP_i, pH 7.0<br>10-400 µM *p*-coumaroyl-CoA<br>10 mM shikimic acid<br>0.26 µg NbHCT1	injection volume: 50 µL<br>UV-detector: λ = 312 nm<br>run time: t = 20 min<br>mobile phase: 35 % methanol, 0.01 % *o*-phosphoric acid<br>flow rate: 1 mL/min<br>stationary phase: Equisil ODS C18 5 µm, 250 x 4 mm
shikimic acid	*p*-coumaroyl-CoA [3.3]		360 mM KP_i, pH 7.0<br>200 µM *p*-coumaroyl-CoA<br>0.5-16 mM shikimic acid<br>0.26 µg NbHCT1	
p-cou-maroyl-CoA	quinic acid [15]	ϑ = 30 °C<br>t = 10 min,<br>negative control:<br>t = 0 min<br>*n* = 3 x 3	130 mM KP_i, pH 7.0<br>10-400 µM *p*-coumaroyl-CoA<br>450 mM quinic acid<br>2.0 µg NbHCT1	injection volume: 50 µL<br>DAD-detector: λ = 312 nm<br>run time: t = 20 min<br>mobile phase: 20 % methanol, 0.01 % *o*-phosphoric acid<br>flow rate: 1 mL/min
quinic acid	*p*-coumaroyl-CoA [6.7]		130 mM KP_i, pH 7.0	

<table>
<tr><td></td><td></td><td></td><td>200 µM p-coumaroyl-CoA
10-450 mM quinic acid
2 µg NbHCT1</td><td>stationary phase: Hypersil ODS C18 5 µm, 250 x 4 mm
column temperature: ϑ = 35 °C</td></tr>
<tr><td>p-coumaroyl-CoA</td><td>3-hydroxy-antranilate [53]</td><td rowspan="2">ϑ = 30 °C
t = 20 min,
negative control: t = 0 min
n = 3 x 3</td><td>32 mM KP_i, pH 7.0
5-200 µM p-coumaroyl-CoA
12 mM 3-hydroxy-antranilate
6 µg NbHCT1</td><td>injection volume: 50 µL
DAD-detector: λ = 312 nm
run time: t = 15 min
mobile phase: 45 % methanol, 0.01 % o-phosphoric acid
flow rate: 1 mL/min
stationary phase: Hypersil ODS C18 5 µm, 250 x 4 mm
column temperature: ϑ = 35 °C</td></tr>
<tr><td>3-hydroxy-antranilate</td><td>p-coumaroyl-CoA [41]</td><td>32 mM KP_i, pH 7.0
200 µM p-coumaroyl-CoA
0.3-12 mM 3-hydroxy-antranilate
6 µg NbHCT1</td><td>injection volume: 30 µL
DAD-detector: λ = 312 nm
run time: t = 15 min
mobile phase: 45 % methanol, 0.01 % o-phosphoric acid
flow rate: 1 mL/min
stationary phase: Hypersil ODS C18 5 µm, 250 x 4 mm
column temperature: ϑ = 35 °C</td></tr>
<tr><td colspan="5">NbHCT3</td></tr>
<tr><td>caffeoyl-CoA</td><td>shikimic acid [2.6]</td><td rowspan="3">ϑ = 35 °C
t = 30 min,
negative control: t = 0 min
n = 3</td><td>32 mM KP_i, pH 7.0
5-200 µM caffeoyl-CoA
600 mM shikimic acid
2.5 µg NbHCT3</td><td rowspan="2">injection volume: 30 µL
DAD-detector: λ = 333 nm
run time: t = 20 min
mobile phase: 30 % methanol, 0.01 % o-phosphoric acid
flow rate: 1 mL/min
stationary phase: Hypersil ODS C18 5 µm, 250 x 4 mm
column temperature: ϑ = 35 °C</td></tr>
<tr><td>shikimic acid</td><td>caffeoyl-CoA [12]</td><td>32 mM KP_i, pH 7.0
200 µM caffeoyl-CoA
20-600 mM shikimic acid
2.5 µg NbHCT3</td></tr>
<tr><td>p-coumaroyl-CoA</td><td>shikimic acid [4.1]</td><td>32 mM KP_i, pH 7.0
5-200 µM p-coumaroyl-CoA
600 mM shikimic acid
2.5 µg NbHCT3</td><td rowspan="2">injection volume: 30 µL
DAD-detector: λ = 312 nm
run time: t = 20 min
mobile phase: 30 % methanol, 0.01 % o-phosphoric acid
flow rate: 1 mL/min</td></tr>
<tr><td>shikimic acid</td><td>p-coumaroyl-CoA [11]</td><td>ϑ = 35 °C
t = 60 min,
negative control:</td><td>32 mM KP_i, pH 7.0</td></tr>
</table>

		t = 0 min<br>*n* = 3	200 µM *p*-coumaroyl-CoA 20-600 mM shikimic acid<br>2.5 µg NbHCT3	stationary phase: Hypersil ODS C18 5 µm, 250 x 4 mm<br>column temperature: ϑ = 35 °
caffeoyl-CoA	quinic acid [57]	ϑ = 35 °C<br>t = 5 min,<br>negative control: t = 0 min<br>*n* = 3 x 3	32 mM KP_i, pH 7.0<br>1-100 µM caffeoyl-CoA<br>100 mM quinic acid<br>0.2 µg NbHCT3	injection volume: 20 µL<br>DAD-detector: λ = 333 nm<br>run time: t = 10 min<br>mobile phase: 20 % methanol, 0.01 % *o*-phosphoric acid<br>flow rate: 1 mL/min<br>stationary phase: Hypersil ODS C18 5 µm, 250 x 4 mm<br>column temperature: ϑ = 35 °
quinic acid	caffeoyl-CoA [8.7]		32 mM KP_i, pH 7.0<br>100 µM caffeoyl-CoA<br>0.1-100 mM quinic acid<br>0.2 µg NbHCT3	
p-coumaroyl-CoA	quinic acid [44]		32 mM KP_i, pH 7.0<br>1-200 µM *p*-coumaroyl-CoA<br>200 mM quinic acid<br>0.2 µg NbHCT3	injection volume: 20 µL<br>DAD-detector: λ = 312 nm<br>run time: t = 10 min<br>mobile phase: 20 % methanol, 0.01 % *o*-phosphoric acid<br>flow rate: 1 mL/min<br>stationary phase: Hypersil ODS C18 5 µm, 250 x 4 mm<br>column temperature: ϑ = 35 °
quinic acid	*p*-coumaroyl-CoA [6.0]		32 mM KP_i, pH 7.0<br>200 µM *p*-coumaroyl-CoA<br>1-200 mM quinic acid<br>0.2 µg NbHCT3	
**NbHCT8**				
caffeoyl-CoA	shikimic acid [8.6]	ϑ = 40 °C<br>t = 10 min,<br>negative control: t = 0 min<br>*n* = 3 x 3	32 mM KP_i, pH 7.0<br>1-100 µM caffeoyl-CoA<br>20 mM shikimic acid<br>1.0 µg NbHCT8	injection volume: 50 µL<br>DAD-detector: λ = 333 nm<br>run time: t = 30 min<br>mobile phase: 20 % methanol, 0.01 % *o*-phosphoric acid<br>flow rate: 1 mL/min<br>stationary phase: Hypersil ODS C18 5 µm, 250 x 4 mm<br>column temperature: ϑ = 35 °
shikimic acid	caffeoyl-CoA [18]		32 mM KP_i, pH 7.0<br>200 µM caffeoyl-CoA<br>0.5-30 mM shikimic acid<br>1.0 µg NbHCT8	
p-coumaroyl-CoA	shikimic acid [8.0]	ϑ = 40 °C<br>t = 20 min,<br>negative control: t = 0 min<br>*n* = 3 x 3	32 mM KP_i, pH 7.0<br>2-200 µM *p*-coumaroyl-CoA 8 mM shikimic acid<br>0.3 µg NbHCT8	injection volume: 50 µL<br>DAD-detector: λ = 312 nm<br>run time: t = 20 min<br>mobile phase: 30 % methanol, 0.01 % *o*-phosphoric acid<br>flow rate: 1 mL/min

				stationary phase: Hypersil ODS C18 5 µm, 250 x 4 mm<br>column temperature: ϑ = 35 °
shikimic acid	*p*-coumaroyl-CoA [10]	ϑ = 40 °C<br>t = 10 min,<br>negative control: t = 0 min<br>*n* = 3 x 3	32 mM KP_i, pH 7.0<br>200 µM *p*-coumaroyl-CoA 0.2-20 mM shikimic acid<br>0.3 µg NbHCT8	injection volume: 50 µL<br>DAD-detector: λ = 312 nm<br>run time: t = 15 min<br>mobile phase: 30 % methanol, 0.01 % *o*-phosphoric acid<br>flow rate: 1 mL/min<br>stationary phase: Hypersil ODS C18 5 µm, 250 x 4 mm<br>column temperature: ϑ = 35 °
p-coumaroyl-CoA	quinic acid [63]	ϑ = 40 °C<br>t = 8 min,<br>negative control: t = 0 min<br>*n* = 3 x 3	32 mM KP_i, pH 7.0<br>10-300 µM *p*-coumaroyl-CoA 300 mM quinic acid<br>1 µg NbHCT8	injection volume: 50 µL<br>DAD-detector: λ = 312 nm<br>run time: t = 15 min<br>mobile phase: 20 % methanol, 0.01 % *o*-phosphoric acid<br>flow rate: 1 mL/min<br>stationary phase: Hypersil ODS C18 5 µm, 250 x 4 mm<br>column temperature: ϑ = 35 °
quinic acid	*p*-coumaroyl-CoA [19]		32 mM KP_i, pH 7.0<br>200 µM *p*-coumaroyl-CoA<br>5-200 mM quinic acid<br>1 µg NbHCT8	
p-coumaroyl-CoA	3-hydroxy-anthranilate [9.8]	ϑ = 40 °C<br>t = 10 min,<br>negative control: t = 0 min<br>*n* = 3 x 3	32 mM KP_i, pH 7.0<br>2-100 µM *p*-coumaroyl-CoA<br>3000 µM 3-hydroxyanthranilate<br>2 µg NbHCT8	injection volume: 50 µL<br>DAD-detector: λ = 312 nm<br>run time: t = 20 min<br>mobile phase: 45 % methanol, 0.01 % *o*-phosphoric acid<br>flow rate: 1 mL/min<br>stationary phase: Hypersil ODS C18 5 µm, 250 x 4 mm<br>column temperature: ϑ = 35 °
3-hydroxy-anthranilate	*p*-coumaroyl-CoA [29]		32 mM KP_i, pH 7.0<br>100 µM *p*-coumaroyl-CoA<br>75-3000 µM 3-hydroxyanthranilate<br>2 µg NbHCT8	

References

Abrankó, L. and Clifford, M.N. (2017) An Unambiguous Nomenclature for the Acyl-quinic Acids Commonly Known as Chlorogenic Acids. *J. Agric. Food Chem.*, 65, 3602–3608. https://doi.org/10.1021/acs.jafc.7b00729

Berger, A., Meinhard, J. and Petersen, M. (2006) Rosmarinic acid synthase is a new member of the superfamily of BAHD acyltransferases. *Planta*, 224, 1503–1510. https://doi.org/10.1007/s00425-006-0393-y

Cornish-Bowden, A. and Eisenthal, R. (1978) Estimation of Michaelis constant and maximum velocity from the direct linear plot. *Biochim. Biophys. Acta*, 523, 268–272. https://doi.org/10.1016/0005-2744(78)90030-X

Kruse, L.H., Weigle, A.T., Irfan, M., Martínez-Gómez, J., Chobirko, J.D., Schaffer, J.E., Bennett, A.A., Specht, C.D., Jez, J.M., Shukla, D. and Moghe, G.D. (2022) Orthology-based analysis helps map evolutionary diversification and predict substrate class use of BAHD acyltransferases. *Plant J.*, 111, 1453–1468. https://doi.org/10.1111/tpj.15902

Levsh, O., Pluskal, T., Carballo, V., Mitchell, A.J. and Weng, J.K. (2019) Independent evolution of rosmarinic acid biosynthesis in two sister families under the Lamiids clade of flowering plants. *J. Biol. Chem.*, 294, 15193–15205. https://doi.org/10.1074/jbc.RA119.010454

Sander, M. (2010) Molekularbiologische und biochemische Untersuchungen von Hydroxycinnamoyltransferasen aus *Coleus blumei* und *Glechoma hederacea*. Dissertation, Philipps-Universität Marburg. https://doi.org/10.17192/z2010.0168

Zhou, J., Zou, X., Deng, Z. and Duan, L. (2024) Analysing a Group of Homologous BAHD Enzymes Provides Insights into the Evolutionary Transition of Rosmarinic Acid Synthases from Hydroxycinnamoyl-CoA:Shikimate/Quinate Hydroxycinnamoyl Transferases. *Plants*, 13, 512. https://doi.org/10.3390/plants13040512
